# Supplementary material for: Slow Chromatin Dynamics Allow Polycomb Target Genes to Filter Fluctuations in Transcription Factor Activity
Source: Cell Syst. 2017 Apr 26;4(4):445–457.e8. doi: 10.1016/j.cels.2017.02.013 (PMC5409831; doi:10.1016/j.cels.2017.02.013)
Supplement: Document S1. Figures S1–S8 and Tables S1–S5 [file mmc1.pdf]

**Cell Systems, Volume *4***

## Supplemental Information

# Slow Chromatin Dynamics Allow Polycomb Target Genes to Filter Fluctuations in Transcription Factor Activity

**Scott Berry, Caroline Dean, and Martin Howard**

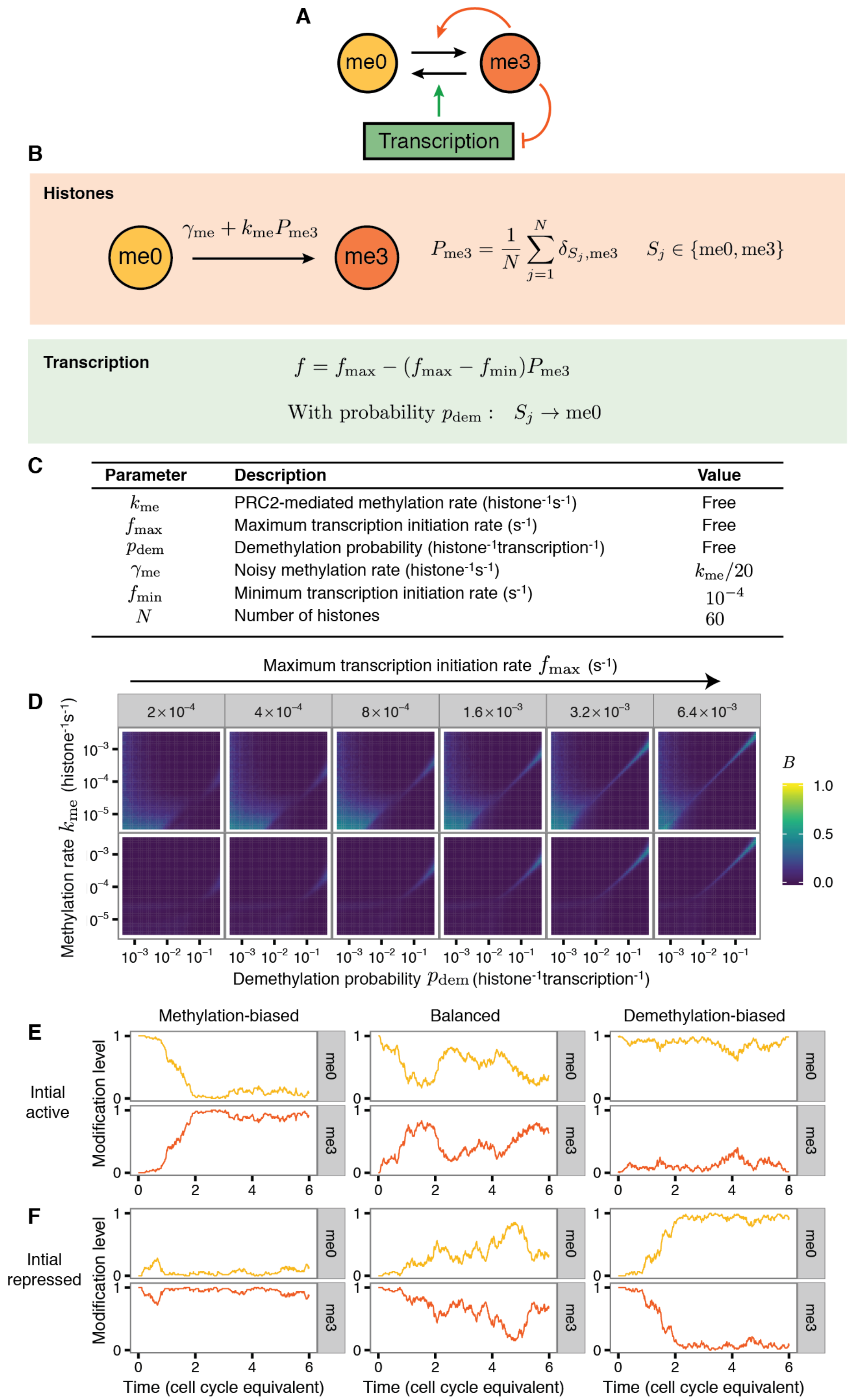


**Figure S1, related to Figure 1: Two-state model.** (A) Diagrammatic representation of feedbacks in the two-state mathematical model. States me0 and me3 refer to the methylation state of H3K27. Black arrows represent state transitions, while coloured arrows represent feedback interactions. (B) Mathematical

description of the model. (C) Two-state model parameters. (D) Heat-map indicating the bistability measure, $B$, for simulations performed with $k_{\mathrm{me}}$ (histone$^{-1}$s$^{-1}$), $p_{\mathrm{dem}}$ (histone$^{-1}$ transcription$^{-1}$), and $f_{\mathrm{max}}$ (s$^{-1}$) values indicated on axes and panel labels. For each parameter set, 100 simulations were initialized in each of the uniform me0 or me3 states and simulated for 50 cell cycles. Bistability measure $B$ calculated as described in STAR Methods. DNA replication is not included in the top panels but is included in the bottom panels, with a timescale of 22 hours (Posakony et al., 1977). (E) Example simulations of a single genomic locus initialized in the active (me0) state, for methylation-biased ($p_{\mathrm{dem}} = 6.3\times10^{-3}$ histone$^{-1}$ transcription$^{-1}$), balanced ($p_{\mathrm{dem}} = 10^{-2}$ histone$^{-1}$ transcription$^{-1}$), or demethylation-biased ($p_{\mathrm{dem}} = 1.5\times10^{-2}$ histone$^{-1}$ transcription$^{-1}$) parameter sets. (F) As in E, except with initially repressed (me3) state. In all examples, $k_{me} = 1.25\times10^{-4}$ histone$^{-1}$s$^{-1}$ and $f_{\mathrm{max}} = 0.0128$ s$^{-1}$.

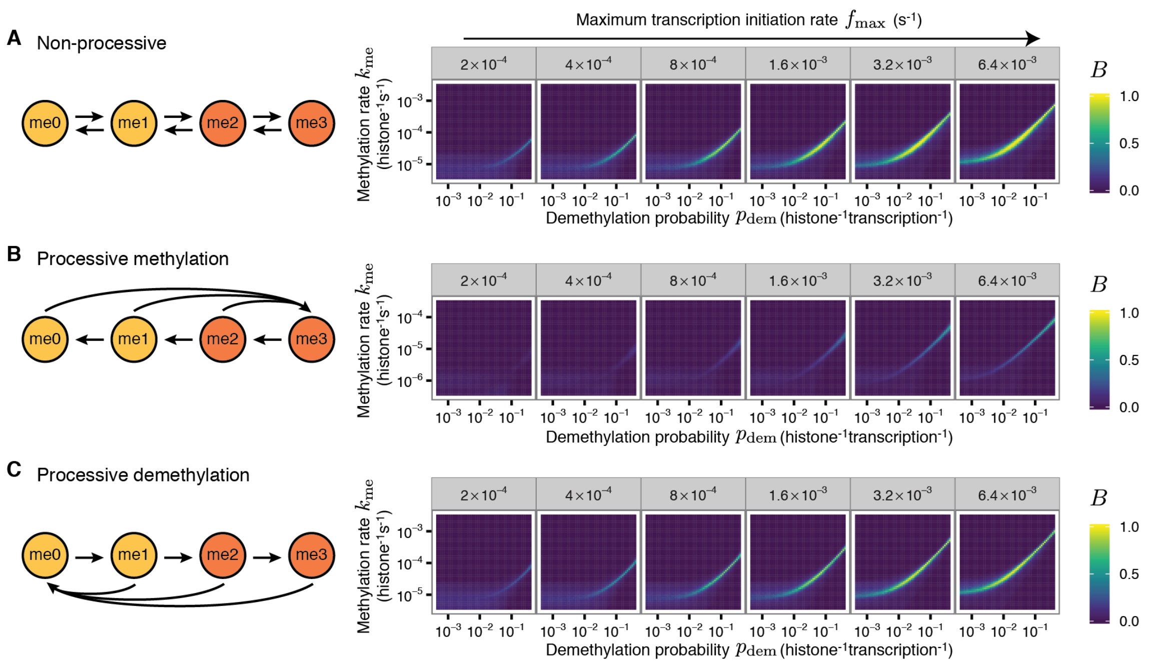


**Figure S2, related to Figure 1: Effect of processivity in enzyme activity on bistability.** Left panels show model schematics with black lines indicating possible state transitions. Right panels show heat maps of the bistability measure, $B$, calculated from simulations performed over a range of values for the parameters $k_{\mathrm{me}}$ (histone$^{-1}$s$^{-1}$), $p_{\mathrm{dem}}$ (histone$^{-1}$ transcription$^{-1}$) and $f_{\max}$ (s$^{-1}$). Each panel shows $B$ as a function of $k_{\mathrm{me}}$ and $p_{\mathrm{dem}}$, for the value of $f_{\max}$ shown in the panel label. For each parameter set, 100 simulations were initialized in each of the uniform me0 or me3 states and simulated for 50 cell cycles. Results averaged over all simulations. All simulations have $p_{\mathrm{ex}} = 10^{-3}$ (histone$^{-1}$ transcription$^{-1}$). Except for changes to processivity, model and other parameters as in Figure 1C and D. (A) Non-processive methylation and demethylation, reproduced from Figure 1G for comparison. (B) Model with only processive methylation, and non-processive demethylation. (C) Model with only processive demethylation, and non-processive methylation.

**A**

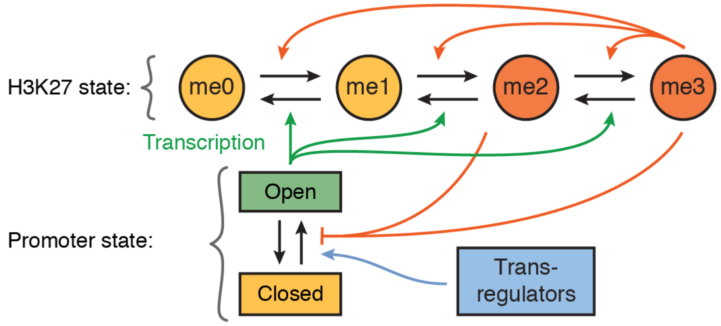


**B**

**Histones**

$$\text{me0} \underset{\gamma_{\text{dem}}}{\overset{\gamma_{\text{me0-1}} + k_{\text{me0-1}} E_i}{\rightleftharpoons}} \text{me1} \underset{\gamma_{\text{dem}}}{\overset{\gamma_{\text{me1-2}} + k_{\text{me1-2}} E_i}{\rightleftharpoons}} \text{me2} \underset{\gamma_{\text{dem}}}{\overset{\gamma_{\text{me2-3}} + k_{\text{me}} E_i}{\rightleftharpoons}} \text{me3}$$

$$E_i = \sum_{j \in \text{neighbors of } i} \left(\rho_{\text{me2}} \delta_{S_j,\text{me2}} + \delta_{S_j,\text{me3}}\right) \qquad S_j \in \{\text{me0}, \text{me1}, \text{me2}, \text{me3}\}$$

**Promoter switching**

$$\text{Closed} \underset{k_{\text{off}}}{\overset{k_{\text{on}}}{\rightleftharpoons}} \text{Open}$$

$$P_{\text{me2/me3}} = \frac{1}{N} \sum_{j=1}^{N} \left(\delta_{S_j,me2} + \delta_{S_j,me3}\right)$$

$$k_{\text{on}} = \begin{cases} \alpha \left(k_{\text{on(max)}} - \frac{P_{\text{me2/me3}}}{P_T} \left(k_{\text{on(max)}} - k_{\text{on(min)}}\right)\right) & , P_{\text{me2/me3}} < P_T \\ \alpha k_{\text{on(min)}} & , P_{\text{me2/me3}} \geq P_T \end{cases}$$

**Transcription**

$$f = \begin{cases} f_0 & \text{, if promoter is "Open"} \\ 0 & \text{, if promoter is "Closed"} \end{cases}$$

$$\text{With probability } p_{\text{dem}} \text{ per histone } j: \quad S_j \rightarrow \begin{cases} \text{me0} & \text{if } S_j = \text{me1} \\ \text{me1} & \text{if } S_j = \text{me2} \\ \text{me2} & \text{if } S_j = \text{me3} \end{cases}$$

$$\text{With probability } p_{\text{ex}} \text{ per histone } j: \quad \begin{cases} S_j S_{j+1} \rightarrow \text{me0}\,\text{me0}, & \text{if } j \in \{1, 3, 5, \ldots, N-1\} \\ S_{j-1} S_j \rightarrow \text{me0}\,\text{me0}, & \text{if } j \in \{2, 4, 6, \ldots, N\} \end{cases}$$

**DNA replication**

$$\text{With probability } \frac{1}{2}: \quad S_j S_{j+1} \rightarrow \text{me0}\,\text{me0}, \text{ for } j = 1, 3, 5, \ldots, N-1$$

**C**

| Parameter | Description | Value |
|---|---|---|
| $k_{\text{on(max)}}$ | Maximum transition rate between 'closed' and 'open' promoter state ($s^{-1}$) | Free |
| $k_{\text{off}}$ | Transition rate between 'open' and 'closed' promoter state ($s^{-1}$) | Free |
| $k_{\text{on(min)}}$ | Minimum transition rate between 'closed' and 'open' promoter state ($s^{-1}$) | $k_{\text{on(max)}} k_{\text{off}} / \left(39 k_{\text{on(max)}} + 40 k_{\text{off}}\right)$ |
| $f_0$ | Transcription initiation rate ('open' promoter) ($s^{-1}$) | $10^{-4} \left(k_{\text{off}} + k_{\text{on(min)}}\right) / k_{\text{on(min)}}$ |

**Figure S3 related to Figure 1: Promoter-switching model.** (A) Diagrammatic representation of feedbacks in mathematical model. States me0 to me3 refer to methylation state of H3K27. Neutral marks me0/me1 indicated in yellow, repressive marks me2/me3 in orange. Black arrows represent state transitions; coloured arrows represent feedback interactions. For clarity, histone exchange and H3K27me2-mediated recruitment of PRC2 are omitted. Promoter states represented as 'open' or 'closed'. Transcription is possible only in the open state. (B) Mathematical description of model. Sum over 'neighbours' in $E_i$

includes the other histone on same nucleosome, and four histones on neighbouring nucleosomes. $\delta_{i,j}$: Kronecker delta, equal to 1 if $i = j$ and 0 otherwise. $P_{\mathrm{me2/me3}}$ is the fraction of H3 histones carrying K27me2 or K27me3. (C) Extra parameters added to the minimal model to incorporate promoter switching. Other parameters defined in Figures 1D and S6M.

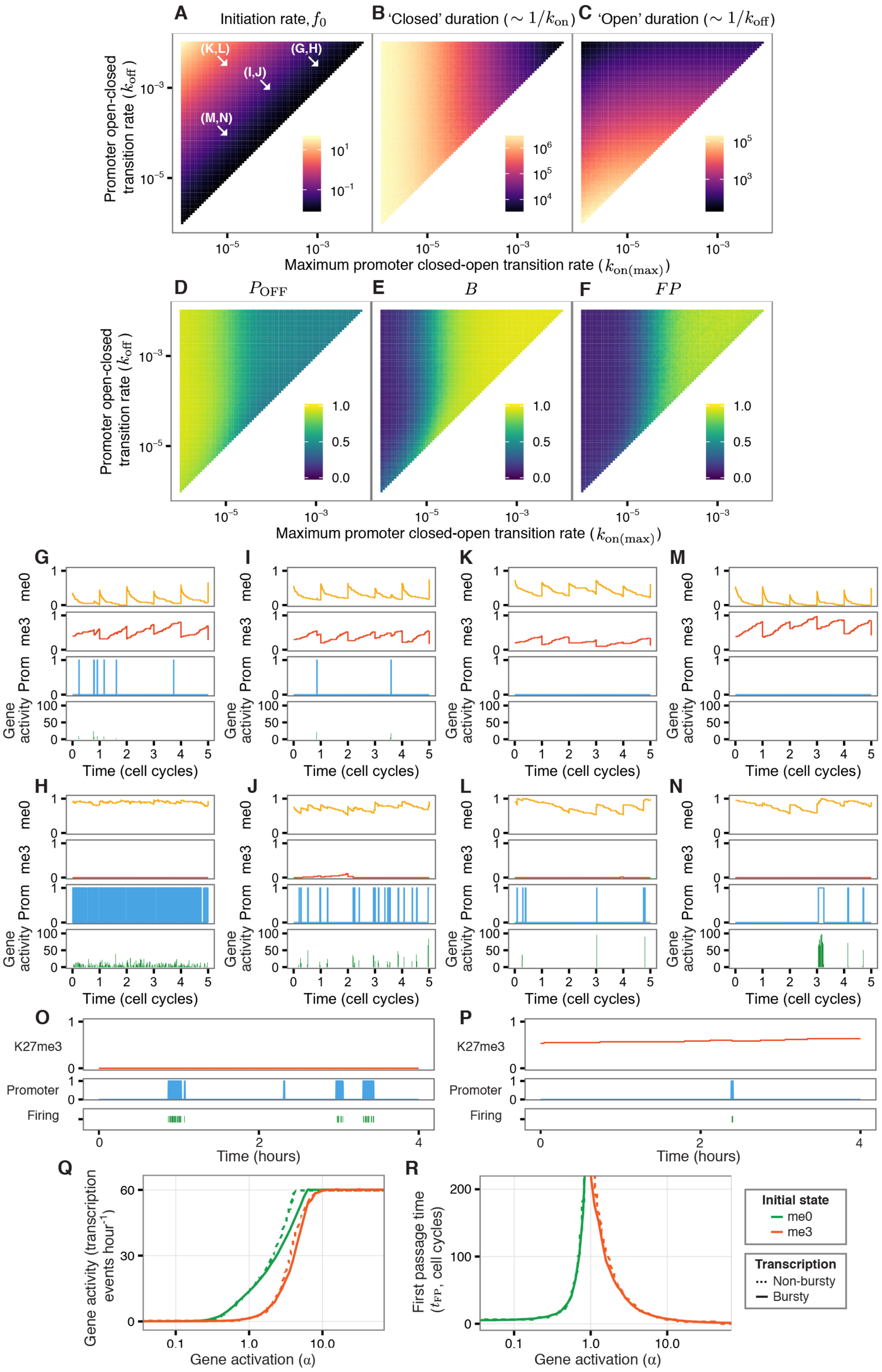


**Figure S4 related to Figures 1 and 2: Promoter-switching model results.** Heat maps showing (A) transcription initiation rate, $f_0$, (B) average duration of a 'closed' state, (C) average duration of an 'open'

state, (D) probability of being in the repressed transcriptional state, (E) bistability measure, $B$ and (F) combined first passage time measure, $FP$. In A, $f_0$ specified as an input parameter while B-F show average results over 500 simulations from each of the uniform me0 and uniform me3 states. Each panel shows results plotted as a function of $k_{\text{on(max)}}$ and $k_{\text{off}}$ ($10^{-2}$ $s^{-1}$ to $10^{-6}$ $s^{-1}$) with simulations restricted to $k_{\text{on(max)}} \leq k_{\text{off}}$. Model defined in Figure S3 with additional parameters in Figures 1D and S6M, ($\alpha = \beta = 1$). (G-N) Example simulations of promoter switching model with variable transcriptional bursting kinetics. Upper row simulations initialised in the repressed (uniform me3) chromatin state, while lower row simulations initialised in the active (uniform me0) chromatin state. Simulations equilibrated for 5 cell cycles before plotting a further 5 cell cycles. Prom (promoter) state represented as 1 for 'open' and 0 for 'closed'. Gene activity represented as the number of transcription events per 30-minute interval. As indicated in A, parameter values are (G, H) $k_{\text{on(max)}} = 10^{-3}\text{s}^{-1}$ , $k_{\text{off}} = 3 \times 10^{-3}$ $\text{s}^{-1}$. (I, J) $k_{\text{on(max)}} = 10^{-4}\text{s}^{-1}$ , $k_{\text{off}} = 10^{-3}$ $\text{s}^{-1}$. (K, L) $k_{\text{on(max)}} = 10^{-5}\text{s}^{-1}$ , $k_{\text{off}} = 3 \times 10^{-3}$ $\text{s}^{-1}$. (M, N) $k_{\text{on(max)}} = 10^{-5}\text{s}^{-1}$ , $k_{\text{off}} = 10^{-4}$ $\text{s}^{-1}$. Other parameters in Figures 1D and S6M. (O) Example time-course simulation over a short time-scale showing H3K27me3 levels, promoter state and transcription initiation events for the promoter-switching model with $k_{\text{on(max)}} = 5 \times 10^{-4}\text{s}^{-1}$ , $k_{\text{off}} = 5 \times 10^{-3}$ $\text{s}^{-1}$. Other parameters in Figures 1D and S6M. Simulation first equilibrated for 5.5 cell cycles from active (uniform me0) initial chromatin state ($\alpha = \beta = 1$). (P) As in O, except initialised in repressed (uniform me3) state. (Q) Gene activity in the promoter-switching model ('Bursty', solid lines) measured as average number of transcription events ($\text{gene}^{-1}\text{hour}^{-1}$) in 20th cell cycle after activation or repression, averaged over 2000 simulations for each value of $\alpha$. Green lines indicate initially active gene; orange lines indicate initially repressed gene. $\alpha = 1$ during 5 cell-cycle equilibration starting from uniform me0 or me3 state, then $\alpha$ as indicated on *x*-axis for further 20 cell cycles. (R) Mean first passage time in the promoter-switching model ('Bursty', solid lines), $t_{\text{FP}}$ (STAR methods) as function of $\alpha$, averaged over 1000 simulations each of 1500 cell cycles, from initially active or repressed state. $k_{\text{on(max)}} = 5 \times 10^{-4}\text{s}^{-1}$ , $k_{\text{off}} = 5 \times 10^{-3}$ $\text{s}^{-1}$ and $\beta = 1$ throughout Q and R, with other parameters in Figures 1D and S6M. Results from the main model with non-bursty transcription are shown with dashed lines for comparison (replotted from Figure 2C,D –upper panels).

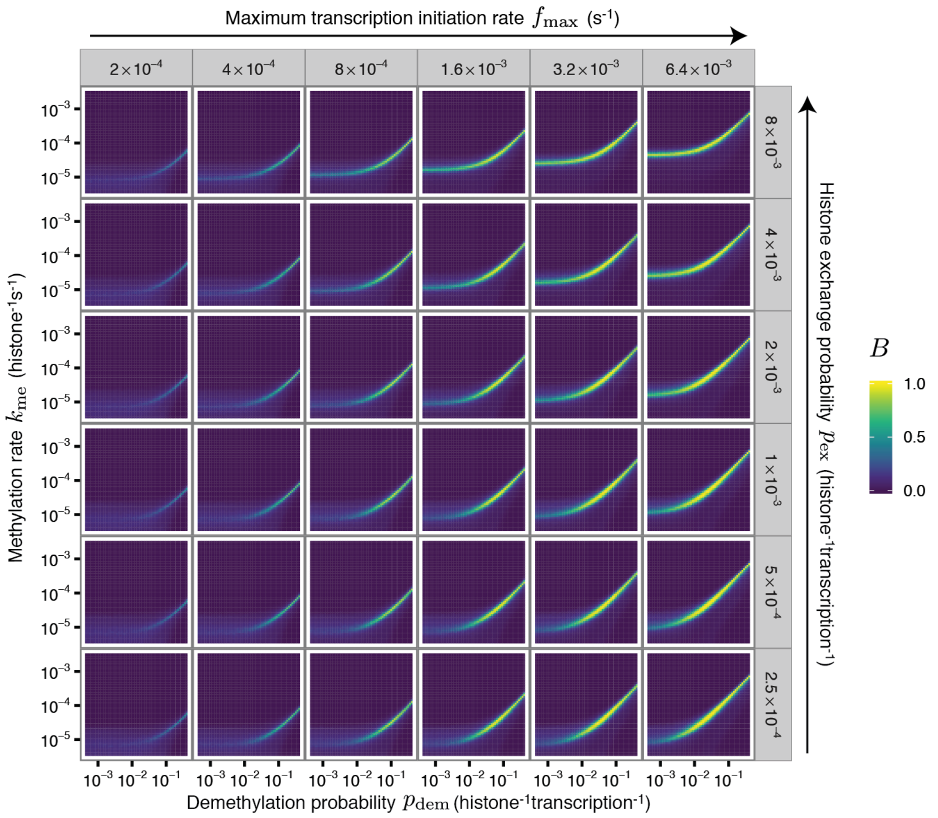


**Figure S5, related to Figure 1: Model bistability for various histone exchange probabilities.** Heat map showing bistability measure $B$, calculated from simulations as described in Figure 1 legend. Each panel shows $B$ as function of $k_{\mathrm{me}}$ and $p_{\mathrm{dem}}$, for $f_{\max}$ and $p_{\mathrm{ex}}$ values shown in panel labels (top and right, respectively).

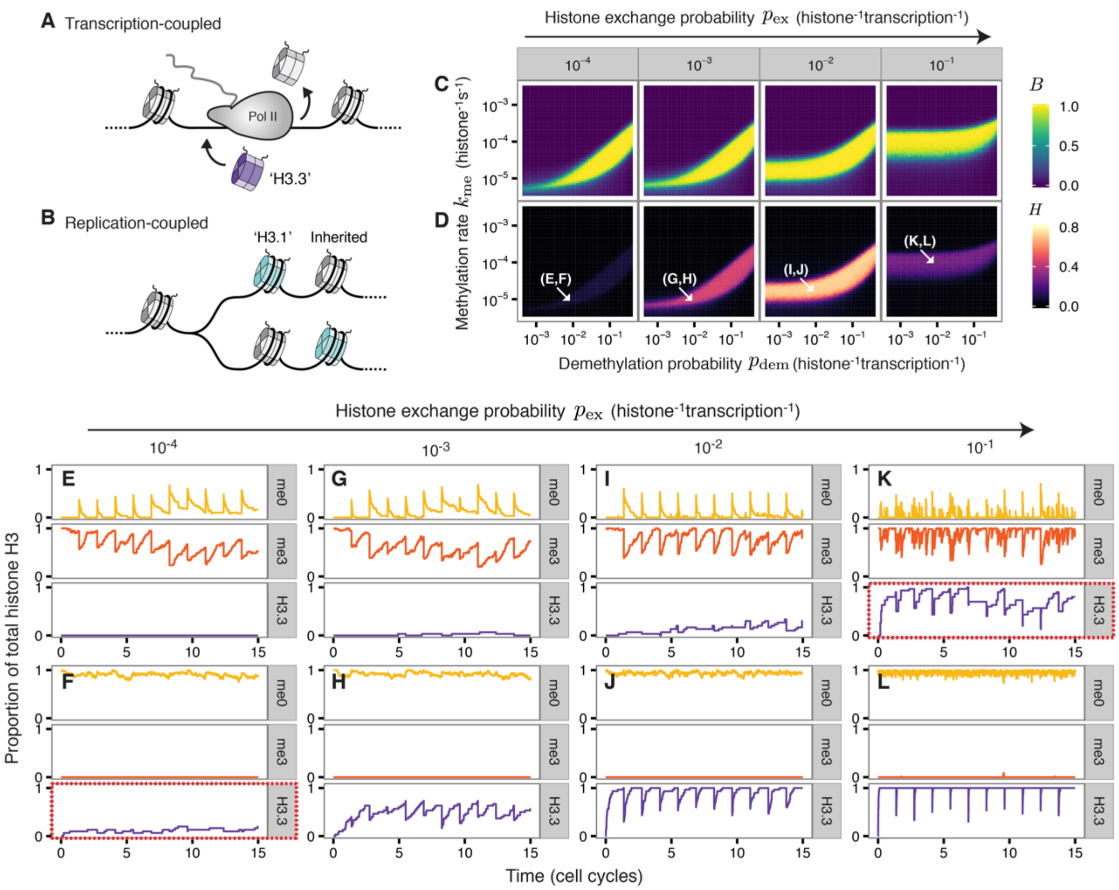


**M**

| Parameter | Description | Value |
|---|---|---|
| $k_{me}$ | PRC2-mediated methylation rate (me2 to me3) (histone$^{-1}$s$^{-1}$) | $8 \times 10^{-6}$ |
| $f_{max}$ | Maximum transcription initiation rate (s$^{-1}$) | $4 \times 10^{-3} (40 f_{min})$ |
| $p_{dem}$ | Demethylation probability (histone$^{-1}$transcription$^{-1}$) | $4 \times 10^{-3}$ |
| $p_{ex}$ | Histone exchange probability (histone$^{-1}$transcription$^{-1}$) | $10^{-3}$ |
| $P_T$ | Threshold proportion of me2/me3 marks for maximal repression | $1/3$ |

**Figure S6, related to Figure B1: Fitting the histone exchange rate to reproduce transcription-dependent H3.3 accumulation.** (A) Schematic of transcription-coupled histone exchange, resulting in H3.3 incorporation (purple nucleosome). (B) Schematic of replication-coupled deposition of H3.1 (cyan nucleosomes). (C) Bistability measure, $B$, and (D) difference in average H3.3 levels between simulations initialized in the active state and those initialized in the repressed state ($H = |\langle \text{H3.3} \rangle_{\text{ON}} - \langle \text{H3.3} \rangle_{\text{OFF}}|$, where $\langle\ \rangle$ indicates an average over time). In C,D, 100 simulations were initialized in each of the uniform me0/H3.1 or me3/H3.1 states and simulated for 50 cell cycles for each parameter set. Each panel shows $B$ or $H$ as function of $k_{\text{me}}$ and $p_{\text{dem}}$, for $p_{\text{ex}}$ shown in panel label. Model as in Figure 1 (as modified by Equation 3). $P_T = 1/3$, $f_{\text{max}} = 40 f_{\text{min}}$, with other parameters in Figure 1D. (E-L) Example simulations for parameters indicated in D. Top row initialized in the uniform me3/H3.1 state. Bottom row initialized in the uniform me0/H3.1 state. (E,F) $k_{\text{me}} = 10^{-5}$ histone$^{-1}$s$^{-1}$, $p_{\text{ex}} = 10^{-4}$ histone$^{-1}$ transcription$^{-1}$. (G,H) $k_{\text{me}} = 10^{-5}$ histone$^{-1}$s$^{-1}$, $p_{\text{ex}} = 10^{-3}$ histone$^{-1}$ transcription$^{-1}$. (I,J) $k_{\text{me}} = 2 \times 10^{-5}$ histone$^{-1}$s$^{-1}$, $p_{\text{ex}} = 10^{-2}$ histone$^{-1}$ transcription$^{-1}$. (K,L) $k_{\text{me}} = 10^{-4}$ histone$^{-1}$s$^{-1}$, $p_{\text{ex}} = 10^{-1}$ histone$^{-1}$ transcription$^{-1}$. Red boxes in F

and K indicate lack of H3.3 in the active state, and H3.3 accumulation in the repressed state, respectively. For E-L, $p_{\mathrm{dem}} = 10^{-2}$ histone$^{-1}$ transcription$^{-1}$. (M) Values of parameters after fitting the model to SILAC data and H3.3 accumulation, and optimizing for bistability.

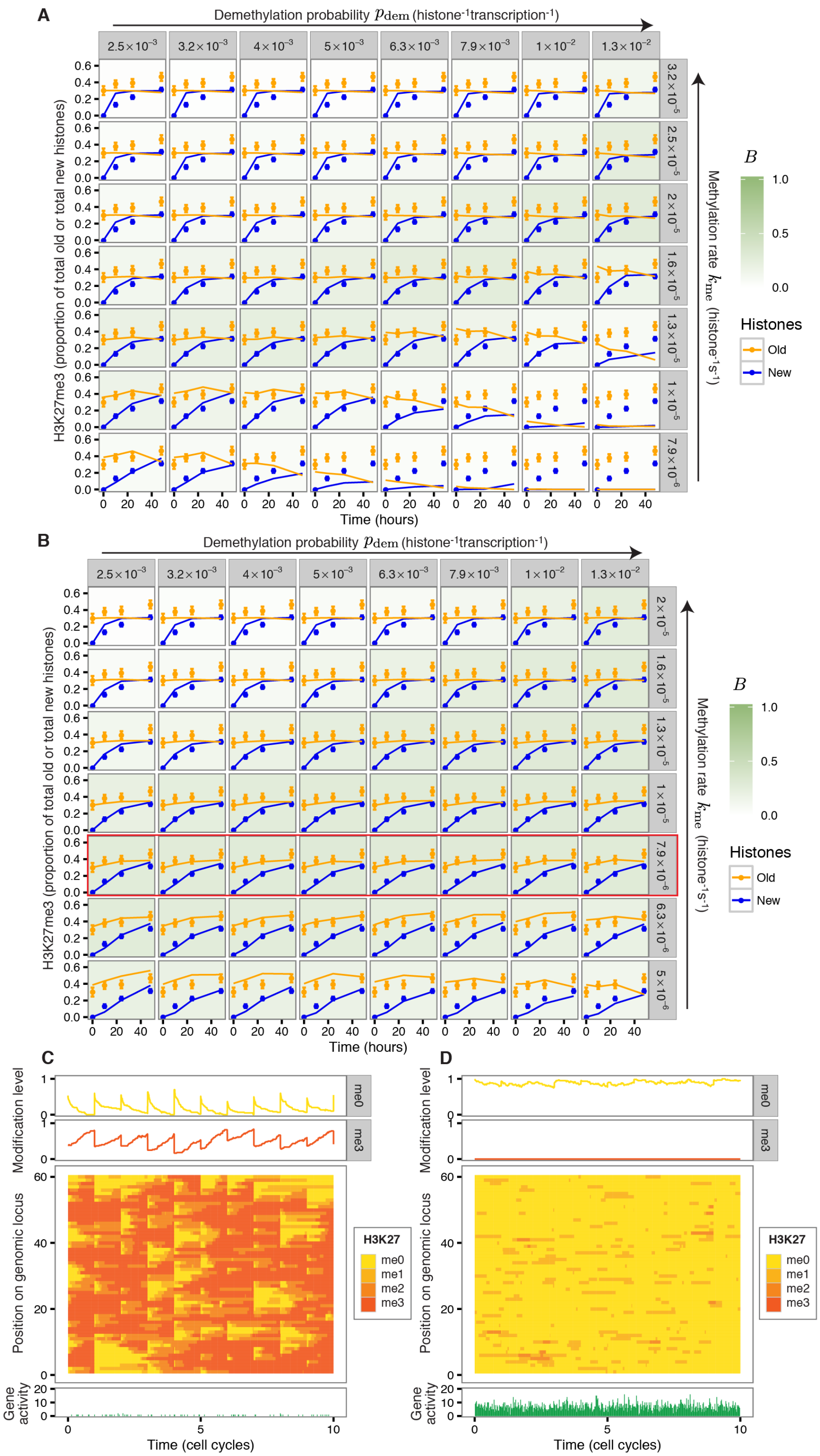


**Figure S7, related to Figure B1: Fitting quantitative SILAC data.** (A) Detailed fit to data over parameter space with $P_T = 1$. Each panel shows the experimentally determined H3K27me3 level on old

and new histones 0, 10, 24 and 48 hours after the first DNA replication in the SILAC experiment. Data are represented as the fraction of H3K27me3 on old and new histones, respectively. Solid lines indicate the model prediction, linearly interpolated between 0, 10, 24, and 48 hour time-points, which are each averages over 1000 SILAC simulations, normalised as described in Figure B1 legend (STAR methods). Each panel shows the results of simulations for a single pair of $k_{\mathrm{me}}$ and $p_{\mathrm{dem}}$ values. $p_{\mathrm{dem}}$ increases from left to right, while $k_{\mathrm{me}}$ increases from bottom to top. Model as in Figure 1 (as modified by Equation 3). $P_T = 1$, $f_{\mathrm{max}} = 40 f_{\mathrm{min}}$, and $p_{\mathrm{ex}} = 10^{-3}$ histone$^{-1}$ transcription$^{-1}$, with other parameters in Figure 1D. The background shading of each panel (green) represents the bistability parameter, $B$, calculated using the same parameters. (B) Same as A, except with $P_T = 1/3$. Red box indicates methylation rate that gave the best fit from values shown (quantitative fit over parameter space shown in Figure B1F). (C,D) Spatially resolved simulations with fitted methylation rate. Example stochastic simulations of the fitted model ($P_T = 1/3$ , $k_{\mathrm{me}} = 8 \times 10^{-6}$ histone$^{-1}$s$^{-1}$, $p_{\mathrm{dem}} = 4 \times 10^{-3}$ histone$^{-1}$ transcription$^{-1}$, $f_{\mathrm{max}} = 40 f_{\mathrm{min}}$, $p_{\mathrm{ex}} = 10^{-3}$ histone$^{-1}$ transcription$^{-1}$), from repressed and active initial states, respectively. Other parameters in Figure 1D. All panels show 10 cell cycles of simulation data, obtained after 5-cell cycles of equilibration. Upper panels show the levels of me0 and me3 over time, averaged over all histones in the locus. Middle panels show kymographs of H3K27 methylation status over time for each histone in the simulated region. Lower panels show gene activity measured as number of transcription events per 30 min interval.

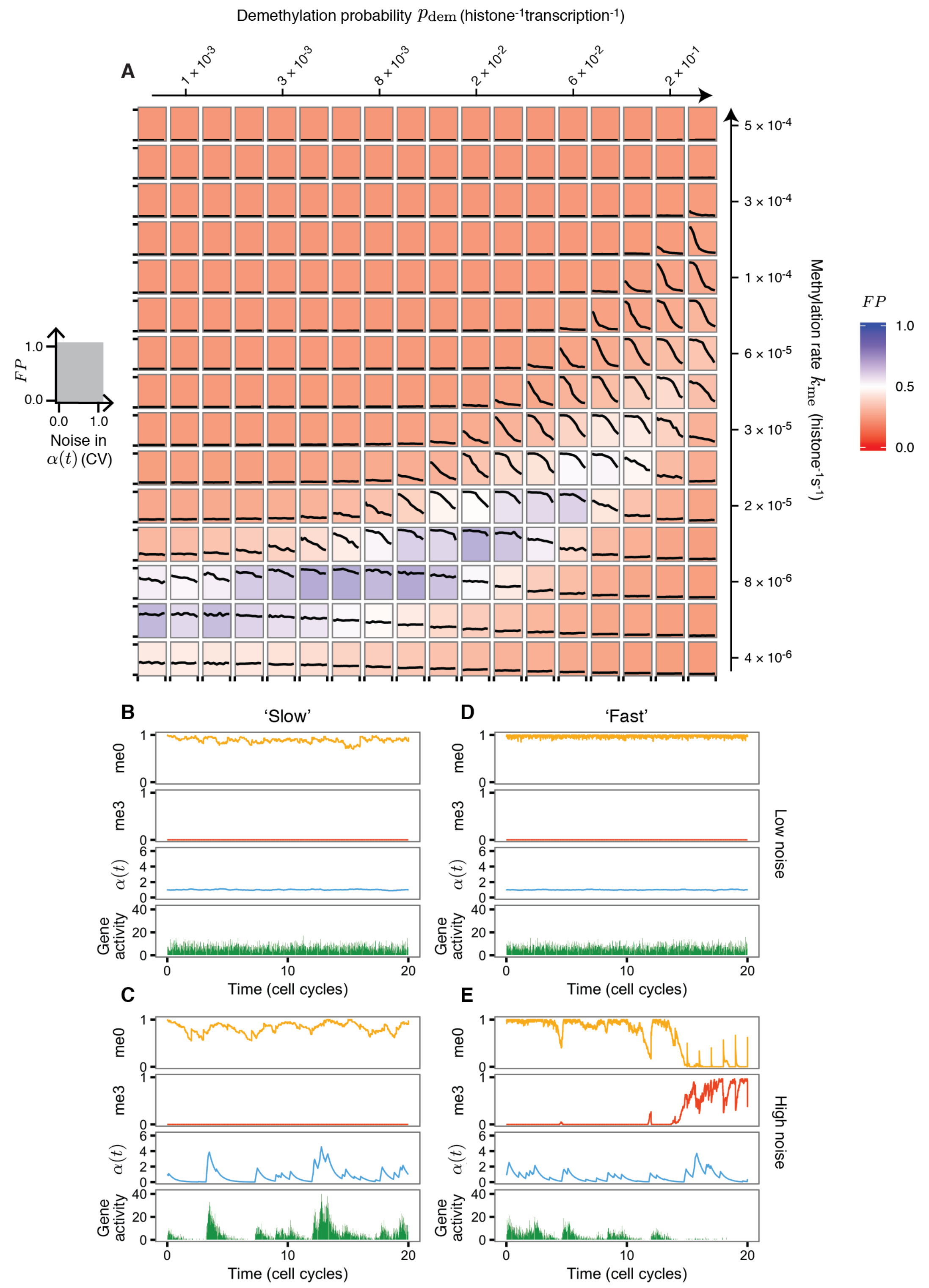


**Figure S8, related to Figure 3: Effect of noisy input signal on memory-storage capability.** (A) Each postage-stamp panel shows the combined first passage time measure, $FP$ as a function of the noise in the

gene activation input signal $\alpha(t)$, plotted as a black line. Noise is measured as the coefficient of variation (CV) in input signal $\alpha(t)$. In all cases, the time-average $\langle\alpha(t)\rangle = 1$. For each parameter set, 100 simulations were initialized in each of the uniform me0 or me3 states and simulated for 25 cell cycles. The schematic postage-stamp panel shown in grey to the left indicates the values of $FP$ and noise that are represented by the axis ticks in each panel. Each panel represents a model with different values of $k_{\text{me}}$ and $p_{\text{dem}}$. $k_{\text{me}}$ increases from bottom to top (log scale) while $p_{\text{dem}}$ increases from left to right (log scale) – as indicated right and above, respectively. The background colour of each panel represents $FP$ for a noisy input signal ($b = 1195$, CV $\approx 1$) for the $k_{\text{me}}$, $p_{\text{dem}}$ values of that panel, where $b$ is defined in STAR methods. Blue (red) represents stable (unstable) chromatin states at high noise levels. Model and other parameters defined in Figure 1 (as modified by Equation 3) and Figures 1D and S6M ($\beta = 1$). (B-E) Example simulations with active initial state. Same as Figure 3B-E, except with active (uniform me0) initial states.

**Table S1, related to Figure 1: Additional references for model formulation.** Evidence is preferentially provided for mammalian Polycomb systems. Further supporting evidence from other biological systems is provided where the mammalian evidence is missing or incomplete. SUZ12, EZH2, EED, and JARID2 are core subunits of mammalian PRC2.

**Model diagram with features labelled**

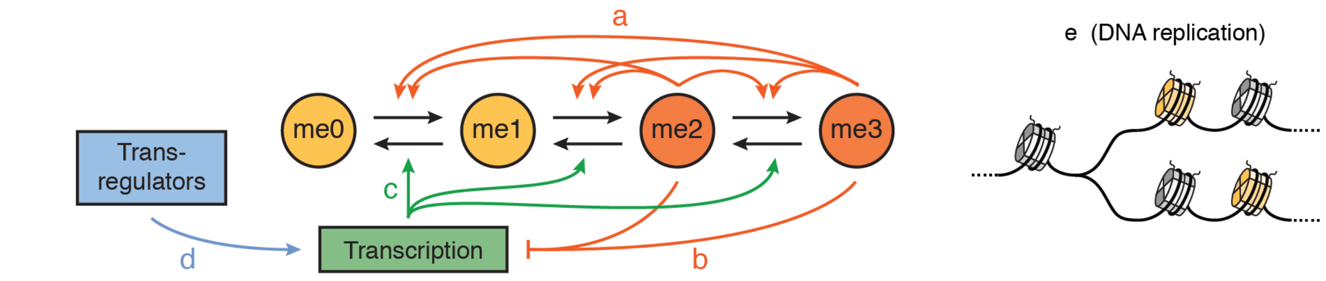


| **Model feature** | **Model assumption** | **Comments** | **Biological system** | **Evidence** |
|---|---|---|---|---|
| a | PRC2 methylates H3K27 | Firmly established | Mammalian | **Biochemical:**<br>• Purified human PRC2 methylates H3K27 *in vitro* (Cao et al., 2002; Kuzmichev et al., 2002).<br>• Catalytic efficiency of non-processive methylation of H3K27, H3K27me1, H3K27me2 substrates by human PRC2 determined *in vitro* (McCabe et al., 2012).<br>**Genetic:**<br>• PRC2 is required for all H3K27me2 and H3K27me3 *in vivo* (Ferrari et al., 2014; Jung et al., 2010; Pasini et al., 2007; Schoeftner et al., 2006), and intragenic H3K27me1 (Ferrari et al., 2014) in mouse embryonic stem (ES) cells. |

| | | | | |
|---|---|---|---|---|
| | | | | **Correlation:**<br>• SUZ12 and EED binding are correlated with H3K27me3 in human ES cells (Lee et al., 2006) and mouse embryonic fibroblasts (Boyer et al., 2006).<br>**Specific cases:**<br>• Tethering EZH2 (Hansen et al., 2008), EED (Hansen et al., 2008; van der Vlag and Otte, 1999) or JARID2 (Pasini et al., 2010a) to a reporter gene can initiate H3K27me3 accumulation and gene repression. |
| a | PRC2 is activated by binding H3K27me2 and H3K27me3 | Well characterized *in vitro*, also with genetic evidence. | Mammalian | **Biochemical:**<br>• Binding of EED to H3K27me2 and H3K27me3 increases catalytic activity of human PRC2 *in vitro* (Margueron et al., 2009).<br>**Genetic:**<br>• Disruption of H3K27me3-binding by EED decreases H3K27me3 levels in human and mouse cells, and leads to embryonic lethality in mice (Ueda et al., 2016). |
| | | | Drosophila | **Genetic:**<br>• H3K27me2/me3 recognition by ESC (EED homologue) is required for PRC2 function *in vivo* (Margueron et al., 2009). |

**Table S2, related to Figure 1: Additional references for model formulation.** Labeled model diagram provided in Table S1.

| **Model feature** | **Model assumption** | **Comments** | **Biological system** | **Evidence** |
|---|---|---|---|---|
| b | H3K27me2/me3 and PRC2 repress transcription | Functionally well established yet poorly understood mechanistically. | Mammalian | **Biochemical:**<br>• Mouse PRC1 components (Grau et al., 2011) and human PRC2 (Margueron et al., 2008) can compact chromatin *in vitro*.<br>• Human PRC2 can repress transcription of chromatinized templates *in vitro* (Margueron et al., 2008).<br>**Genetic:**<br>• Mutation of SUZ12 or EED results in increased acetylation (Pasini et al., 2010b) and expression (Boyer et al., 2006; Pasini et al., 2007; Shen et al., 2008) of PRC2 target genes in mouse ES cells. However, it should also be noted that a more recent study has reported that PRC2 is dispensable for repression of PRC2 targets in certain culture conditions (but is still required during differentiation) (Riising et al., 2014).<br>• Treatment of human cells with a small molecule inhibitor of Ezh2 leads to a loss of H3K27me2/me3 and activation of PRC2 targets (Qi et al., 2012).<br>• Mutation of PRC1 or PRC2 subunits results in loss of chromatin compaction and changes in chromatin topology at Hox loci in mouse ES cells (Eskeland et al., 2010; Williamson et al., 2014).<br>**Correlation:**<br>• H3K27me3 and PRC2 occupancy are inversely correlated with markers of productive transcription, such as accumulation of mRNA (Brookes et al., 2012; Lee et al., 2006), histone acetylation (Pasini et al., 2010b), and RNA polymerase II phosphorylated on Ser-2 of the C-terminal domain (CTD) (Brookes et al., 2012) in ES cells. |

| | | | | |
|---|---|---|---|---|
| | | | | • H3K27me3 is correlated with reduced chromatin accessibility, as measured by DNAse I mapping in diverse human cells (Roadmap Epigenomics Consortium et al., 2015), or MNase accessibility (MACC) in mouse ES cells (Deaton et al., 2016).<br><br>**Specific case:**<br><br>• Tethering EZH2 (Hansen et al., 2008), EED (Hansen et al., 2008; van der Vlag and Otte, 1999) or JARID2 (Pasini et al., 2010a) to a reporter gene can initiate H3K27me3 accumulation and gene repression. |
| | | | *Drosophila* | **Biochemical:**<br><br>• PRC1 components compact chromatin (Francis et al., 2004), and inhibit chromatin remodeling *in vitro* (Francis et al., 2001).<br>• PRC1 can repress transcription *in vitro* (King et al., 2002).<br><br>**Genetic:**<br><br>• Lys-27 of H3 is required for PRC2-mediated gene repression (Pengelly et al., 2013).<br>• Mutation of ESC (EED homologue) leads to increased occupancy of RNA polymerase II and decreases in H3K27me3 at PRC2 target-gene promoters (Chopra et al., 2011).<br><br>**Correlation:**<br><br>• Polycomb silencing is associated with chromatin compaction and the formation of “long-range” intra-chromosomal contacts (Boettiger et al., 2016; Sexton et al., 2012). |

**Table S3, related to Figure 1: Additional references for model formulation.** Labeled model diagram provided in Table S1.

| **Model feature** | **Model assumption** | **Comments** | **Biological system** | **Evidence** |
|---|---|---|---|---|
| c | Transcription antagonizes H3K27me3 accumulation | Core assumption of our model. We cite experimental results in support of our proposal that the mechanistic basis of this antagonism is through transcription-coupled H3K27-demethylation and histone exchange. | Mammalian | **Specific cases:**<br>• Global transcriptional inhibition using small molecules results in PRC2 recruitment to new targets genome-wide in mouse ES cells (Riising et al., 2014).<br>• Transgenic reporter gene studies indicate that the transcription start site of the c-Jun locus is required for displacement of PRC2 during differentiation (Riising et al., 2014).<br>• Transcriptional induction by retinoic acid (RA) of *CYP26a1* (mouse ES cells) or *CYP26a1*, *Hoxa1*, *RARβ2* (mouse P9 embryonic carcinoma cells) results in H3K27me3 reduction (Gillespie and Gudas, 2007; Yuan et al., 2012). Conversely, H3K27me3 accumulates slowly at these genes after removal of RA.<br>• In human NIH 3T3 cells, changes in expression of PRC2 targets induced by Ras signalling precede changes in gene-body H3K27me3 levels (Hosogane et al., 2013).<br><br>**<u>Histone demethylation</u>**<br><br>**Biochemical:**<br>• Human JMJD3 and UTX demethylate H3K27me3 non-processively *in vitro* (Agger et al., 2007; Hong et al., 2007; Lee et al., 2007). JMJD3 associates with transcription elongation factors in human cells (Chen et al., 2012).<br><br>**Genetic:**<br>• Over-expression of human UTX reduces H3K27me2/me3 *in vivo* (Hong et al., 2007). |

| | | | | |
|---|---|---|---|---|
| | | | | **Specific cases:**<br>• UTX is bound to several *HOX* promoters (Agger et al., 2007; Lan et al., 2007) and levels increase at the *Hoxb1* locus during gene induction, resulting in H3K27me3-demethyation and loss of PRC2 (Agger et al., 2007).<br>• UTX required to maintain expression and low H3K27me2/me3 levels at *Hoxa13* and *Hoxc4* (Lee et al., 2007).<br>**<u>Histone exchange</u>**<br>**Correlations:**<br>• H3.3 histones are incorporated independently of replication in human cells and relative H3.3 levels have been regarded as a marker of histone exchange (Ray-Gallet et al., 2011; Tagami et al., 2004). H3.3 accumulation is positively correlated with transcriptional activity in human cells (Pchelintsev et al., 2013; Ray-Gallet et al., 2011).<br>• Histone exchange and H3.3 accumulation is positively correlated with transcriptional activity in mouse ES cells and neural stem cells (Deaton et al., 2016), and mouse embryonic fibroblasts (MEFs) (Kraushaar et al., 2013).<br>• Both H3.3 levels and histone exchange are negatively correlated with H3K27me3 (Kraushaar et al., 2013) in MEFs and with Polycomb complex binding in mouse ES cells (Deaton et al., 2016). |
| | | | *Arabidopsis* | **Specific case:**<br>• Exogenously driven transcriptional induction can remove H3K27me3 at *FLC*. Conversely, transcriptional shutdown from a high-expression state results in accumulation of H3K27me3 (Buzas et al., 2011). |
| | | | *S cerevisiae* | **<u>Histone exchange</u>** |

| | | | | |
|---|---|---|---|---|
| | | | | **Correlation:**<br>• Histone exchange rates correlate with gene expression (Dion et al., 2007; Jamai et al., 2007). |
| | | | *Drosophila* | **<u>Histone demethylation</u>**<br>**Biochemical:**<br>• Drosophila UTX colocalises with elongating RNA polymerase II (Smith et al., 2008).<br>**<u>Histone exchange</u>**<br>**Correlation:**<br>• Histone exchange rates correlate with gene expression (Deal et al., 2010) |

**Table S4, related to Figure 1: Additional references for model formulation.** Labeled model diagram provided in Table S1.

| **Model feature** | **Model assumption** | **Comments** | **Biological system** | **Evidence** |
|---|---|---|---|---|
| d | Trans-regulators directly modulate transcription | Firmly established | Various | • In prokaryotes and eukaryotes, transcription factors can directly drive recruitment of pre-initiation complexes (reviewed in (Ptashne and Gann, 1997)) or, in eukaryotes, they can act through distal regulatory elements (reviewed in (Heintzman and Ren, 2009)).<br>• Trans-regulation can be graded in an 'analog' fashion according to dosage of a single regulator in both mammals (Giorgetti et al., 2010) and yeast (Stewart-Ornstein et al., 2013). |

**Table S5, related to Figure 1: Additional references for model formulation.** Labeled model diagram provided in Table S1.

| Model feature | Model assumption | Comments | Biological system | Evidence |
|---|---|---|---|---|
| e | H3/H4 tetramers are inherited at DNA replication, and are distributed with equal probability to the two daughter strands. New H3/H4 tetramers without pre-existing H3K27-methylation are inserted to fill the gaps. | Firmly established at the level of bulk chromatin. Local inheritance of H3/H4 tetramers (at individual genomic locations) is understudied. | Mammalian | • H3/H4 tetramers do not dissociate during DNA replication and segregate between DNA strands (Jackson, 1987; 1990; Jackson and Chalkley, 1985; Yamasu and Senshu, 1990) (reviewed in (Annunziato, 2005))<br>• H3K27me3 levels on parental histones are diluted by one-half immediately after DNA replication in HeLa cells, and accumulate slowly over the cell cycle (Alabert et al., 2015).<br>• H3K27-methylation is not detected before histones are incorporated into chromatin (Jasencakova et al., 2010; Loyola et al., 2006). |
| | | | *C elegans* | • H3K27-methylated histones are passed on and shared equally between daughter chromosomes during embryogenesis in the absence of PRC2 (Gaydos et al., 2014). |
| | | | *S cerevisiae* | • Parental histones are inherited relatively close to their original location (within around 400 base-pairs) (Radman-Livaja et al., 2011). |

## Supplemental References


Agger, K., Cloos, P.A.C., Christensen, J., Pasini, D., Rose, S., Rappsilber, J., Issaeva, I., Canaani, E., Salcini, A.E., and Helin, K. (2007). UTX and JMJD3 are histone H3K27 demethylases involved in HOX gene regulation and development. Nature *449*, 731–734.

Alabert, C., Barth, T.K., Reverón-Gómez, N., Sidoli, S., Schmidt, A., Jensen, O.N., Imhof, A., and Groth, A. (2015). Two distinct modes for propagation of histone PTMs across the cell cycle. Genes Dev. *29*, 585–590.

Annunziato, A.T. (2005). Split decision: what happens to nucleosomes during DNA replication? J. Biol. Chem. *280*, 12065–12068.

Boettiger, A.N., Bintu, B., Moffitt, J.R., Wang, S., Beliveau, B.J., Fudenberg, G., Imakaev, M., Mirny, L.A., Wu, C.-T., and Zhuang, X. (2016). Super-resolution imaging reveals distinct chromatin folding for different epigenetic states. Nature *529*, 418–422.

Boyer, L.A., Plath, K., Zeitlinger, J., Brambrink, T., Medeiros, L.A., Lee, T.I., Levine, S.S., Wernig, M., Tajonar, A., Ray, M.K., et al. (2006). Polycomb complexes repress developmental regulators in murine embryonic stem cells. Nature *441*, 349–353.

Brookes, E., de Santiago, I., Hebenstreit, D., Morris, K.J., Carroll, T., Xie, S.Q., Stock, J.K., Heidemann, M., Eick, D., Nozaki, N., et al. (2012). Polycomb associates genome-wide with a specific RNA polymerase II variant, and regulates metabolic genes in ESCs. Cell Stem Cell *10*, 157–170.

Buzas, D.M., Robertson, M., Finnegan, E.J., and Helliwell, C.A. (2011). Transcription-dependence of histone H3 lysine 27 trimethylation at the Arabidopsis polycomb target gene FLC. Plant J. *65*, 872–881.

Cao, R., Wang, L., Wang, H., Xia, L., Erdjument-Bromage, H., Tempst, P., Jones, R.S., and Zhang, Y. (2002). Role of histone H3 lysine 27 methylation in Polycomb-group silencing. Science *298*, 1039–1043.

Chen, S., Ma, J., Wu, F., Xiong, L.-J., Ma, H., Xu, W., Lv, R., Li, X., Villén, J., Gygi, S.P., et al. (2012). The histone H3 Lys 27 demethylase JMJD3 regulates gene expression by impacting transcriptional elongation. Genes Dev. *26*, 1364–1375.

Chopra, V.S., Hendrix, D.A., Core, L.J., Tsui, C., Lis, J.T., and Levine, M. (2011). The polycomb group mutant esc leads to augmented levels of paused Pol II in the Drosophila embryo. Mol. Cell *42*, 837–844.

Deal, R.B., Henikoff, J.G., and Henikoff, S. (2010). Genome-wide kinetics of nucleosome turnover determined by metabolic labeling of histones. Science *328*, 1161–1164.

Deaton, A.M., Gómez-Rodríguez, M., Mieczkowski, J., Tolstorukov, M.Y., Kundu, S., Sadreyev, R.I., Jansen, L.E., and Kingston, R.E. (2016). Enhancer regions show high histone H3.3 turnover that changes during differentiation. eLife *5*, e15316.

Dion, M.F., Kaplan, T., Kim, M., Buratowski, S., Friedman, N., and Rando, O.J. (2007). Dynamics of replication-independent histone turnover in budding yeast. Science *315*, 1405–1408.

Eskeland, R., Leeb, M., Grimes, G.R., Kress, C., Boyle, S., Sproul, D., Gilbert, N., Fan, Y., Skoultchi, A.I., Wutz, A., et al. (2010). Ring1B compacts chromatin structure and represses gene expression independent of histone ubiquitination. Mol. Cell *38*, 452–464.

Ferrari, K.J., Scelfo, A., Jammula, S., Cuomo, A., Barozzi, I., Stützer, A., Fischle, W., Bonaldi, T., and Pasini, D. (2014). Polycomb-dependent H3K27me1 and H3K27me2 regulate active transcription and

enhancer fidelity. Mol. Cell *53*, 49–62.

Francis, N.J., Saurin, A.J., Shao, Z., and Kingston, R.E. (2001). Reconstitution of a functional core polycomb repressive complex. Mol. Cell *8*, 545–556.

Francis, N.J., Kingston, R.E., and Woodcock, C.L. (2004). Chromatin compaction by a polycomb group protein complex. Science *306*, 1574–1577.

Gaydos, L.J., Wang, W., and Strome, S. (2014). H3K27me and PRC2 transmit a memory of repression across generations and during development. Science *345*, 1515–1518.

Gillespie, R.F., and Gudas, L.J. (2007). Retinoid regulated association of transcriptional co-regulators and the polycomb group protein SUZ12 with the retinoic acid response elements of Hoxa1, RARbeta(2), and Cyp26A1 in F9 embryonal carcinoma cells. J. Mol. Biol. *372*, 298–316.

Giorgetti, L., Siggers, T., Tiana, G., Caprara, G., Notarbartolo, S., Corona, T., Pasparakis, M., Milani, P., Bulyk, M.L., and Natoli, G. (2010). Noncooperative Interactions between Transcription Factors and Clustered DNA Binding Sites Enable Graded Transcriptional Responses to Environmental Inputs. Mol. Cell *37*, 418–428.

Grau, D.J., Chapman, B.A., Garlick, J.D., Borowsky, M., Francis, N.J., and Kingston, R.E. (2011). Compaction of chromatin by diverse Polycomb group proteins requires localized regions of high charge. Genes Dev. *25*, 2210–2221.

Hansen, K.H., Bracken, A.P., Pasini, D., Dietrich, N., Gehani, S.S., Monrad, A., Rappsilber, J., Lerdrup, M., and Helin, K. (2008). A model for transmission of the H3K27me3 epigenetic mark. Nat. Cell Biol. *10*, 1291–1300.

Heintzman, N.D., and Ren, B. (2009). Finding distal regulatory elements in the human genome. Curr. Opin. Genet. Dev. *19*, 541–549.

Hong, S., Cho, Y.-W., Yu, L.-R., Yu, H., Veenstra, T.D., and Ge, K. (2007). Identification of JmjC domain-containing UTX and JMJD3 as histone H3 lysine 27 demethylases. Proc. Natl. Acad. Sci. USA *104*, 18439–18444.

Hosogane, M., Funayama, R., Nishida, Y., Nagashima, T., and Nakayama, K. (2013). Ras-Induced Changes in H3K27me3 Occur after Those in Transcriptional Activity. PLoS Genet. *9*, e1003698–16.

Jackson, V. (1987). Deposition of newly synthesized histones: new histones H2A and H2B do not deposit in the same nucleosome with new histones H3 and H4. Biochemistry *26*, 2315–2325.

Jackson, V. (1990). In vivo studies on the dynamics of histone-DNA interaction: evidence for nucleosome dissolution during replication and transcription and a low level of dissolution independent of both. Biochemistry *29*, 719–731.

Jackson, V., and Chalkley, R. (1985). Histone segregation on replicating chromatin. Biochemistry *24*, 6930–6938.

Jamai, A., Imoberdorf, R.M., and Strubin, M. (2007). Continuous Histone H2B and Transcription-Dependent Histone H3 Exchange in Yeast Cells outside of Replication. Mol. Cell *25*, 345–355.

Jasencakova, Z., Scharf, A.N.D., Ask, K., Corpet, A., Imhof, A., Almouzni, G., and Groth, A. (2010). Replication stress interferes with histone recycling and predeposition marking of new histones. Mol. Cell *37*, 736–743.

Jung, H.R., Pasini, D., Helin, K., and Jensen, O.N. (2010). Quantitative mass spectrometry of histones H3.2 and H3.3 in Suz12-deficient mouse embryonic stem cells reveals distinct, dynamic post-translational modifications at Lys-27 and Lys-36. Mol. Cell. Proteomics *9*, 838–850.

King, I.F.G., Francis, N.J., and Kingston, R.E. (2002). Native and Recombinant Polycomb Group Complexes Establish a Selective Block to Template Accessibility To Repress Transcription In Vitro. Mol. Cell. Biol. *22*, 7919–7928.

Kraushaar, D.C., Jin, W., Maunakea, A., Abraham, B., Ha, M., and Zhao, K. (2013). Genome-wide incorporation dynamics reveal distinct categories of turnover for the histone variant H3.3. Genome Biol. *14*, R121.

Kuzmichev, A., Nishioka, K., Erdjument-Bromage, H., Tempst, P., and Reinberg, D. (2002). Histone methyltransferase activity associated with a human multiprotein complex containing the Enhancer of Zeste protein. Genes Dev. *16*, 2893–2905.

Lan, F., Bayliss, P.E., Rinn, J.L., Whetstine, J.R., Wang, J.K., Chen, S., Iwase, S., Alpatov, R., Issaeva, I., Canaani, E., et al. (2007). A histone H3 lysine 27 demethylase regulates animal posterior development. Nature *449*, 689–694.

Lee, M.G., Villa, R., Trojer, P., Norman, J., Yan, K.-P., Reinberg, D., Di Croce, L., and Shiekhattar, R. (2007). Demethylation of H3K27 regulates polycomb recruitment and H2A ubiquitination. Science *318*, 447–450.

Lee, T.I., Jenner, R.G., Boyer, L.A., Guenther, M.G., Levine, S.S., Kumar, R.M., Chevalier, B., Johnstone, S.E., Cole, M.F., Isono, K.-I., et al. (2006). Control of developmental regulators by Polycomb in human embryonic stem cells. Cell *125*, 301–313.

Loyola, A., Bonaldi, T., Roche, D., Imhof, A., and Almouzni, G. (2006). PTMs on H3 variants before chromatin assembly potentiate their final epigenetic state. Mol. Cell *24*, 309–316.

Margueron, R., Justin, N., Ohno, K., Sharpe, M.L., Son, J., Drury, W.J.I., Voigt, P., Martin, S.R., Taylor, W.R., De Marco, V., et al. (2009). Role of the polycomb protein EED in the propagation of repressive histone marks. Nature *461*, 762–767.

Margueron, R., Li, G., Sarma, K., Blais, A., Zavadil, J., Woodcock, C.L., Dynlacht, B.D., and Reinberg, D. (2008). Ezh1 and Ezh2 maintain repressive chromatin through different mechanisms. Mol. Cell *32*, 503–518.

McCabe, M.T., Graves, A.P., Ganji, G., Diaz, E., Halsey, W.S., Jiang, Y., Smitheman, K.N., Ott, H.M., Pappalardi, M.B., Allen, K.E., et al. (2012). Mutation of A677 in histone methyltransferase EZH2 in human B-cell lymphoma promotes hypertrimethylation of histone H3 on lysine 27 (H3K27). Proc. Natl. Acad. Sci. USA *109*, 2989–2994.

Pasini, D., Bracken, A.P., Hansen, J.B., Capillo, M., and Helin, K. (2007). The polycomb group protein Suz12 is required for embryonic stem cell differentiation. Mol. Cell. Biol. *27*, 3769–3779.

Pasini, D., Cloos, P.A.C., Walfridsson, J., Olsson, L., Bukowski, J.-P., Johansen, J.V., Bak, M., Tommerup, N., Rappsilber, J., and Helin, K. (2010a). JARID2 regulates binding of the Polycomb repressive complex 2 to target genes in ES cells. Nature *464*, 306–310.

Pasini, D., Malatesta, M., Jung, H.R., Walfridsson, J., Willer, A., Olsson, L., Skotte, J., Wutz, A., Porse, B., Jensen, O.N., et al. (2010b). Characterization of an antagonistic switch between histone H3 lysine 27 methylation and acetylation in the transcriptional regulation of Polycomb group target genes. Nucl. Acids Res. *38*, 4958–4969.

Pchelintsev, N.A., McBryan, T., Rai, T.S., van Tuyn, J., Ray-Gallet, D., Almouzni, G., and Adams, P.D. (2013). Placing the HIRA Histone Chaperone Complex in the Chromatin Landscape. Cell Rep. *3*, 1012–1019.

Pengelly, A.R., Copur, Ö., Jäckle, H., Herzig, A., and Müller, J. (2013). A histone mutant reproduces the phenotype caused by loss of histone-modifying factor Polycomb. Science *339*, 698–699.

Posakony, J.W., England, J.M., and Attardi, G. (1977). Mitochondrial growth and division during the cell cycle in HeLa cells. J. Cell Biol. *74*, 468–491.

Ptashne, M., and Gann, A. (1997). Transcriptional activation by recruitment. Nature *386*, 569–577.

Qi, W., Chan, H., Teng, L., Li, L., Chuai, S., Zhang, R., Zeng, J., Li, M., Fan, H., Lin, Y., et al. (2012). Selective inhibition of Ezh2 by a small molecule inhibitor blocks tumor cells proliferation. Proc. Natl. Acad. Sci. USA *109*, 21360–21365.

Radman-Livaja, M., Verzijlbergen, K.F., Weiner, A., van Welsem, T., Friedman, N., Rando, O.J., and van Leeuwen, F. (2011). Patterns and Mechanisms of Ancestral Histone Protein Inheritance in Budding Yeast. PLoS Biol. *9*, e1001075.

Ray-Gallet, D., Woolfe, A., Vassias, I., Pellentz, C., Lacoste, N., Puri, A., Schultz, D.C., Pchelintsev, N.A., Adams, P.D., Jansen, L.E.T., et al. (2011). Dynamics of histone H3 deposition in vivo reveal a nucleosome gap-filling mechanism for H3.3 to maintain chromatin integrity. Mol. Cell *44*, 928–941.

Riising, E.M., Comet, I., Leblanc, B., Wu, X., Johansen, J.V., and Helin, K. (2014). Gene silencing triggers polycomb repressive complex 2 recruitment to CpG islands genome wide. Mol. Cell *55*, 347–360.

Roadmap Epigenomics Consortium, Kundaje, A., Bilenky, M., Yen, A., Heravi-Moussavi, A., Zhang, Z., Wang, J., Ziller, M.J., Amin, V., Sarkar, A., et al. (2015). Integrative analysis of 111 reference human epigenomes. Nature *518*, 317–330.

Schoeftner, S., Sengupta, A.K., Kubicek, S., Mechtler, K., Spahn, L., Koseki, H., Jenuwein, T., and Wutz, A. (2006). Recruitment of PRC1 function at the initiation of X inactivation independent of PRC2 and silencing. Embo J. *25*, 3110–3122.

Sexton, T., Yaffe, E., Kenigsberg, E., Bantignies, F., Leblanc, B., Hoichman, M., Parrinello, H., Tanay, A., and Cavalli, G. (2012). Three-Dimensional Folding and Functional Organization Principles of the Drosophila Genome. Cell *148*, 458–472.

Shen, X., Liu, Y., Hsu, Y.-J., Fujiwara, Y., Kim, J., Mao, X., Yuan, G.-C., and Orkin, S.H. (2008). EZH1 mediates methylation on histone H3 lysine 27 and complements EZH2 in maintaining stem cell identity and executing pluripotency. Mol. Cell *32*, 491–502.

Smith, E.R., Lee, M.G., Winter, B., Droz, N.M., Eissenberg, J.C., Shiekhattar, R., and Shilatifard, A. (2008). Drosophila UTX is a histone H3 Lys27 demethylase that colocalizes with the elongating form of RNA polymerase II. Mol. Cell. Biol. *28*, 1041–1046.

Stewart-Ornstein, J., Nelson, C., DeRisi, J., Weissman, J.S., and El-Samad, H. (2013). Msn2 coordinates a stoichiometric gene expression program. Curr. Biol. *23*, 2336–2345.

Tagami, H., Ray-Gallet, D., Almouzni, G., and Nakatani, Y. (2004). Histone H3.1 and H3.3 complexes mediate nucleosome assembly pathways dependent or independent of DNA synthesis. Cell *116*, 51–61.

Ueda, T., Nakata, Y., Nagamachi, A., Yamasaki, N., Kanai, A., Sera, Y., Sasaki, M., Matsui, H., Honda, Z.-I., Oda, H., et al. (2016). Propagation of trimethylated H3K27 regulated by polycomb protein EED is

required for embryogenesis, hematopoietic maintenance, and tumor suppression. Proc. Natl. Acad. Sci. USA *113*, 10370–10375.

van der Vlag, J., and Otte, A.P. (1999). Transcriptional repression mediated by the human polycomb-group protein EED involves histone deacetylation. Nat. Genet. *23*, 474–478.

Williamson, I., Berlivet, S., Eskeland, R., Boyle, S., Illingworth, R.S., Paquette, D., Dostie, J., and Bickmore, W.A. (2014). Spatial genome organization: contrasting views from chromosome conformation capture and fluorescence in situ hybridization. Genes Dev. *28*, 2778–2791.

Yamasu, K., and Senshu, T. (1990). Conservative segregation of tetrameric units of H3 and H4 histones during nucleosome replication. J. Biochem. *107*, 15–20.

Yuan, W., Wu, T., Fu, H., Dai, C., Wu, H., Liu, N., Li, X., Xu, M., Zhang, Z., Niu, T., et al. (2012). Dense chromatin activates Polycomb repressive complex 2 to regulate H3 lysine 27 methylation. Science *337*, 971–975.
