# Supplementary material for: Slow Chromatin Dynamics Allow Polycomb Target Genes to Filter Fluctuations in Transcription Factor Activity
Source: Cell Syst. 2017 Apr 26;4(4):445–457.e8. doi: 10.1016/j.cels.2017.02.013 (PMC5409831; doi:10.1016/j.cels.2017.02.013)
Supplement: Document S2. Article plus Supplemental Information [file mmc2.pdf]

Cell Systems

Slow Chromatin Dynamics Allow Polycomb Target Genes to Filter Fluctuations in Transcription Factor Activity

Graphical Abstract

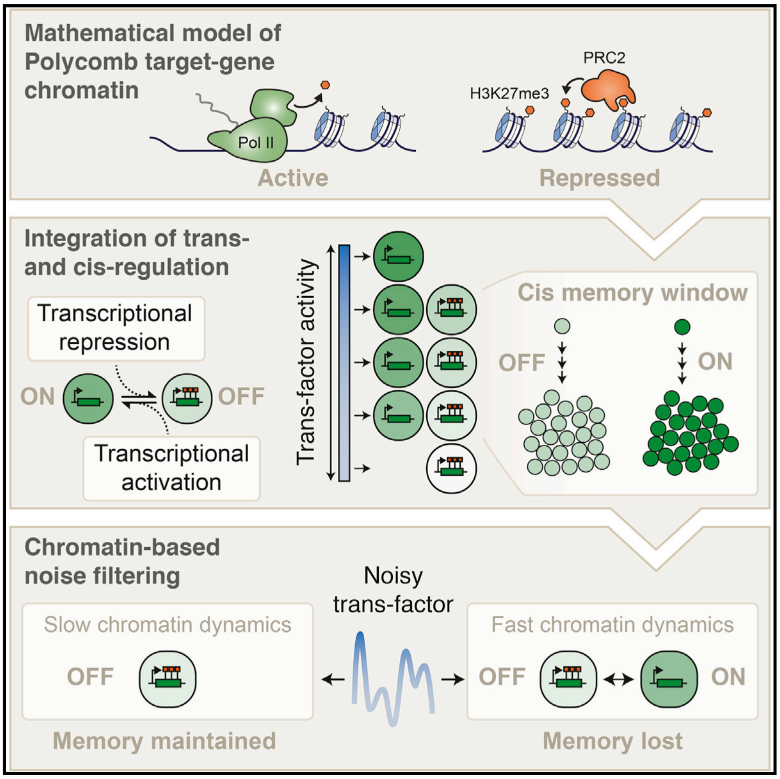

Highlights

- Robust bistable model of chromatin in which transcription antagonizes silencing
- Quantitative fit to experimental slow rate of H3K27me3 accumulation
- Chromatin states are switchable by persistent transcriptional activation or repression
- Slow H3K27 methylation dynamics enable PRC2 targets to filter *trans*-regulatory noise

Authors

Scott Berry, Caroline Dean, Martin Howard

Correspondence

martin.howard@jic.ac.uk

In Brief

We propose a theoretical model in which *trans*-acting and chromatin-based gene regulation are mechanistically integrated by the process of transcription. The model provides insight into how chromatin can respond to conventional *trans*-factor-mediated transcriptional regulation yet also underlie self-perpetuating *cis* epigenetic memory and suggests a role for chromatin in filtering fluctuations of *trans* regulators.

Berry et al., 2017, Cell Systems *4*, 445–457
April 26, 2017 © 2017 The Author(s). Published by Elsevier Inc.
http://dx.doi.org/10.1016/j.cels.2017.02.013

Cell Systems
Math | Bio

OPEN ACCESS
CellPress

Slow Chromatin Dynamics Allow Polycomb Target Genes to Filter Fluctuations in Transcription Factor Activity

Scott Berry,[1,2] Caroline Dean,[1] and Martin Howard[1,3,*]
[1]John Innes Centre, Norwich Research Park, Norwich NR4 7UH, UK
[2]Present address: Institute of Molecular Life Sciences, University of Zurich, 8057 Zurich, Switzerland
[3]Lead Contact
*Correspondence: martin.howard@jic.ac.uk
http://dx.doi.org/10.1016/j.cels.2017.02.013

SUMMARY

Genes targeted by Polycomb repressive complex 2 (PRC2) are regulated in *cis* by chromatin modifications and also in *trans* by diffusible regulators such as transcription factors. Here, we introduce a mathematical model in which transcription directly antagonizes Polycomb silencing, thereby linking these *cis*- and *trans*-regulatory inputs to gene expression. The model is parameterized by recent experimental data showing that PRC2-mediated repressive chromatin modifications accumulate extremely slowly. The model generates self-perpetuating, bistable active and repressed chromatin states that persist through DNA replication, thereby ensuring high-fidelity transmission of the current chromatin state. However, sufficiently strong, persistent activation or repression of transcription promotes switching between active and repressed chromatin states. We observe that when chromatin modification dynamics are slow, transient pulses of transcriptional activation or repression are effectively filtered, such that epigenetic memory is retained. Noise filtering thus depends on slow chromatin dynamics and may represent an important function of PRC2-based regulation.

INTRODUCTION

Models of chromatin-based epigenetic memory are based on the hypothesis that chromatin states determine gene expression (Moazed, 2011). Specific post-translational modifications of histones (histone modifications) that are associated with active and repressed chromatin states are proposed to act as heritable marks that drive re-establishment of the parental chromatin state on daughter chromosomes following DNA replication (Angel et al., 2011; Dodd et al., 2007). In this way, the chromatin state can be maintained through mitotic cell division and thereby maintain a particular expression state of the underlying gene.

There is considerable support for this model in the case of Polycomb repressive complex 2 (PRC2)-dependent gene repression. PRC2 is a multiprotein complex containing an enzymatic subunit that methylates histone H3 at Lys-27 (H3K27) (Kuzmichev et al., 2002), and also a non-catalytic subunit that recognizes H3K27me3 (Margueron et al., 2009). These two activities are proposed to underlie positive feedback between H3K27me3 and PRC2, which contributes to the maintenance of H3K27-methylated chromatin domains (Hansen et al., 2008; Margueron et al., 2009). It has also been shown that histone H3 Lys-27 is required for PRC2-mediated repression (Pengelly et al., 2013), that methylated H3K27 can be passed on to daughter chromosomes (Gaydos et al., 2014), and that tethering of PRC2 subunits to chromatin can initiate transcriptional repression (Hansen et al., 2008; Pasini et al., 2010a). Moreover, two copies of a PRC2 target gene can exist in alternative, heritable expression states in the same cell, indicating that the memory of gene expression can be stored in *cis*—in the local chromatin environment (Berry et al., 2015). Together, these findings suggest that methylation of H3K27 can establish a repressed chromatin state, which can then maintain itself, i.e., a local, *cis*-based epigenetic memory.

In contrast to this model of chromatin-based regulation, it is known that expression of PRC2 target genes can also be controlled by gene-specific regulators acting in *trans* (reviewed in Ringrose, 2007). However, since the process of transcription directly influences chromatin, these *cis*- and *trans*-regulatory modes are not independent. Specifically, studies in mammalian cells have shown that PRC2 and H3K27me3 can accumulate in response to transcriptional repression and can also be removed by transcriptional activation (Gillespie and Gudas, 2007; Hosogane et al., 2013; Riising et al., 2014; Yuan et al., 2012).

To investigate the interplay between *trans*-regulation and chromatin states, we have developed a mathematical model of PRC2-based epigenetic repression in which transcription acts antagonistically to Polycomb silencing. The model represents a generic PRC2 target gene in which the whole locus is enriched in H3K27me2/me3 when repressed (Brookes et al., 2012; Mikkelsen et al., 2007). We constrain the model by quantitatively fitting to time-resolved mass spectrometry data for H3K27me3 accumulation (Alabert et al., 2015). Overall, our analysis demonstrates how *trans*-regulatory signals can be integrated with bistable chromatin states to quantitatively regulate gene expression, yet also provide robust *cis* epigenetic memory.

This is an open access article under the CC BY license (http://creativecommons.org/licenses/by/4.0/).

OPEN ACCESS
CellPress

A

B

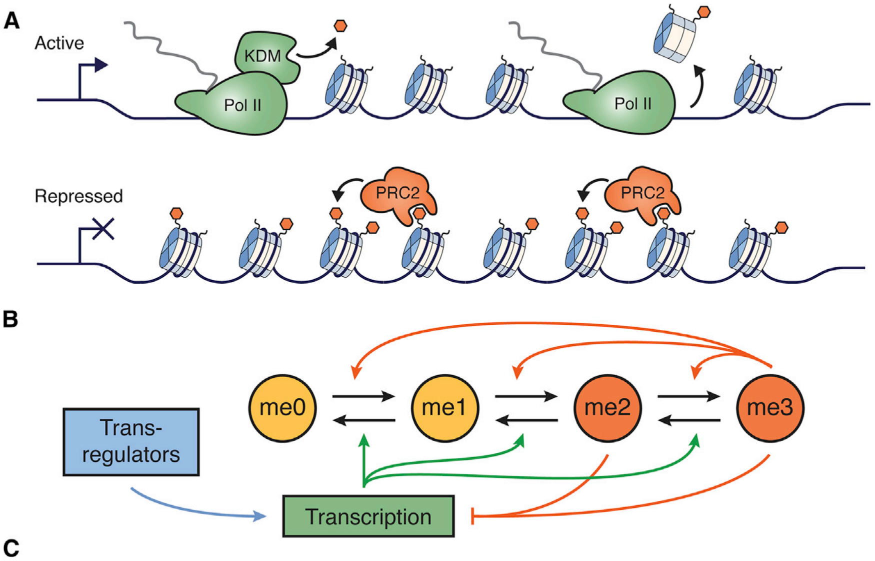

E

F

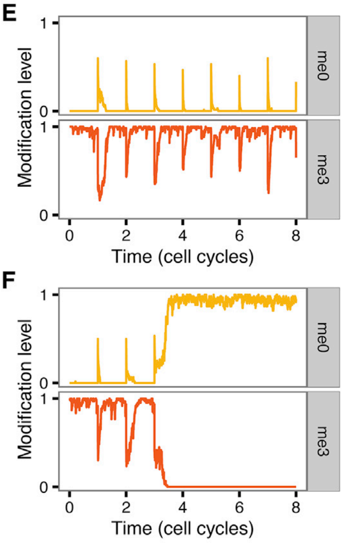

C

Histones

$$\text{me0} \underset{\gamma_{\text{dem}}}{\overset{\beta(\gamma_{\text{me0-1}} + k_{\text{me0-1}} E_i)}{\rightleftharpoons}} \text{me1} \underset{\gamma_{\text{dem}}}{\overset{\beta(\gamma_{\text{me1-2}} + k_{\text{me1-2}} E_i)}{\rightleftharpoons}} \text{me2} \underset{\gamma_{\text{dem}}}{\overset{\beta(\gamma_{\text{me2-3}} + k_{\text{me}} E_i)}{\rightleftharpoons}} \text{me3}$$

$$E_i = \sum_{j \in \text{neighbors of } i} \left(\rho_{\text{me2}} \delta_{S_j,\text{me2}} + \delta_{S_j,\text{me3}}\right) \qquad S_j \in \{\text{me0}, \text{me1}, \text{me2}, \text{me3}\}$$

Transcription

$$f = \alpha\left(f_{\max} - P_{\text{me2/me3}}\left(f_{\max} - f_{\min}\right)\right) \qquad P_{\text{me2/me3}} = \frac{1}{N}\sum_{j=1}^{N}\left(\delta_{S_j,me2} + \delta_{S_j,me3}\right)$$

$$\text{With probability } p_{\text{dem}} \text{ per histone } j: \quad S_j \rightarrow \begin{cases} \text{me0} & \text{if } S_j = \text{me1} \\ \text{me1} & \text{if } S_j = \text{me2} \\ \text{me2} & \text{if } S_j = \text{me3} \end{cases}$$

$$\text{With probability } p_{\text{ex}} \text{ per histone } j: \quad \begin{cases} S_j S_{j+1} \rightarrow \text{me0}\,\text{me0}, & \text{if } j \in \{1,3,5,\ldots,N-1\} \\ S_{j-1} S_j \rightarrow \text{me0}\,\text{me0}, & \text{if } j \in \{2,4,6,\ldots,N\} \end{cases}$$

DNA replication

$$\text{With probability } \frac{1}{2}: \quad S_j S_{j+1} \rightarrow \text{me0}\,\text{me0}, \text{ for } j = 1,3,5,\ldots,N-1$$

D

Parameter	Description	Value
k_{me}	PRC2-mediated methylation rate (me2 to me3) (histone^{-1}s^{-1})	Free
$f_{\max}$	Maximum transcription initiation rate (s^{-1})	Free
p_{dem}	Demethylation probability (histone^{-1}transcription^{-1})	Free
p_{ex}	Histone exchange probability (histone^{-1}transcription^{-1})	Free
$k_{\text{me0-1}}$	PRC2-mediated methylation rate (me0 to me1) (histone^{-1}s^{-1})	$9k_{\text{me}}$
$k_{\text{me1-2}}$	PRC2-mediated methylation rate (me1 to me2) (histone^{-1}s^{-1})	$6k_{\text{me}}$
$\gamma_{\text{me0-1}}$	Noisy methylation rate (me0 to me1) (histone^{-1}s^{-1})	$k_{\text{me0-1}}/20$
$\gamma_{\text{me1-2}}$	Noisy methylation rate (me1 to me2) (histone^{-1}s^{-1})	$k_{\text{me1-2}}/20$
$\gamma_{\text{me2-3}}$	Noisy methylation rate (me2 to me3) (histone^{-1}s^{-1})	$k_{\text{me}}/20$
ρ_{me2}	Relative activation of PRC2 by H3K27me2	$1/10$
γ_{dem}	Noisy demethylation rate (histone^{-1}s^{-1})	$f_{\min} p_{\text{dem}}$
$f_{\min}$	Minimum transcription initiation rate (s^{-1})	10^{-4}
N	Number of histones	60
	Cell cycle length (h)	22
α	Trans-acting gene activation	1
β	Relative local PRC2 activity	1

G

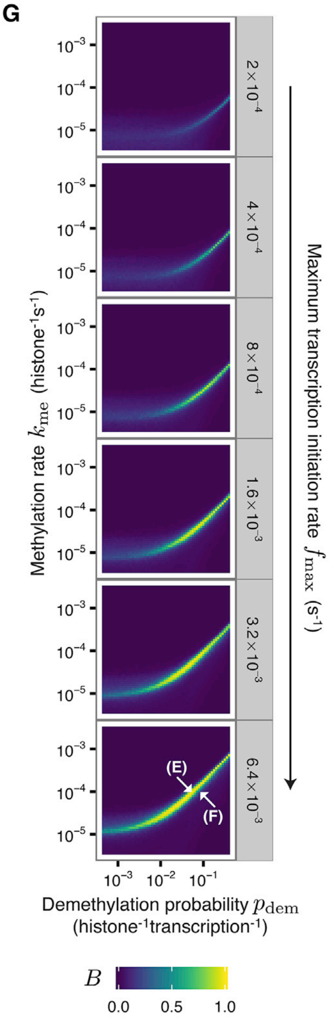

Figure 1. Model of PRC2 Target-Gene Chromatin

(A) Schematic of alternative chromatin states. Active state characterized by presence of Pol II, which can carry H3K27-demethylases (KDM), and drive nucleosome exchange. Repressed state characterized by H3K27me3 (orange hexagons), which can positively feedback to recruit PRC2.

(legend continued on next page)

RESULTS

Previous mathematical models of epigenetic memory based on local inheritance of histone modifications have not explicitly considered the effect of transcription. These models instead rely on mutually exclusive activating and repressive histone modifications (Angel et al., 2011; Dodd et al., 2007): each modification positively feeds back to recruit the enzymatic complexes necessary to place more of the same modification, and also remove the other. In this way, a region of chromatin can be set into one of two states, characterized by high levels of one of the histone modifications.

Here, we hypothesize that transcription itself antagonizes PRC2 activity, without the need for activating histone modifications. Potentially, this system could also generate bistable states: an actively transcribed state (with low H3K27me3) and a poorly transcribed state (with high H3K27me3) (Figure 1A). To investigate this, we formulated a mathematical model and performed stochastic simulations in which we tracked transcriptional initiation events and the H3K27 methylation status for each histone within a region of chromatin. In our model, PRC2 activity results in methylation of H3K27, and transcription results in H3K27 demethylation and histone exchange. H3K27me1/me2 act as intermediates between H3K27me0 and fully methylated H3K27me3 (Figure 1B). Previous theoretical studies have shown that bistability requires nonlinearity in histone modification conversions (Dodd et al., 2007). Incorporating intermediate methylation states naturally generates this nonlinearity because typically more than one feedback transition must occur to convert a given histone between the two extreme states (Dodd et al., 2007; Sneppen and Dodd, 2012). Indeed, we found that a model without these intermediate states was not bistable (STAR Methods, Figure S1). Below we introduce and justify the six main features of our model (Figure 1, Tables S1–S5, STAR Methods). Unless otherwise specified, all references refer to studies in mammalian systems.

Six Model Features

Feature 1: Positive Feedback in H3K27 Methylation Required for Self-Sustaining Repressive States

In addition to catalyzing methylation of H3K27 (Cao et al., 2002; Kuzmichev et al., 2002), PRC2 also binds to H3K27me3 via a non-catalytic subunit, resulting in allosteric activation (Margueron et al., 2009). This positive feedback was included in the model by allowing H3K27me3-modified histones to activate PRC2 to methylate any neighboring histone. Such *cis*-acting positive feedback is fundamental to the model; without it, self-sustaining repressive transcriptional states would not be possible. In agreement with in vitro studies, H3K27me2 is also able to activate PRC2 in the model, but with a 10-fold reduced efficacy (Margueron et al., 2009). H3K27me1 does not activate PRC2 in vitro or in the model (Margueron et al., 2009). The me0/me1 modification states can therefore be grouped as neutral marks and me2/me3 as repressive marks (Figure 1B).

The mechanism by which PRC2 is recruited to its targets is an active area of research and likely to be context-specific (Bauer et al., 2016). Here, we assume that the mechanisms driving PRC2 recruitment (e.g., DNA sequence-specific elements, CpG islands) allow PRC2 to be targeted to the modeled region. This is captured by the parameter β, which represents the relative rates of PRC2 activity between different loci (i.e., strength of recruitment and local enzymatic activity). We initially consider a PRC2 target gene with $\beta = 1$ (in contrast to non-PRC2 targets with $\beta \ll 1$). Putting this together, the rate for the stimulated addition of methylation in our model for the *i*th histone is (Figures 1C and 1D):

$$r^{\mathrm{me}}_{i,\mathrm{stimulated}} = \beta\left(\delta_{S_i,\mathrm{me0}}k_{\mathrm{me0-1}}E_i + \delta_{S_i,\mathrm{me1}}k_{\mathrm{me1-2}}E_i + \delta_{S_i,\mathrm{me2}}k_{\mathrm{me2-3}}E_i\right),$$
$$E_i = \sum_{j\,\in\,\text{neighbors of i}} \left(\rho_{\mathrm{me2}}\delta_{S_j,\mathrm{me2}} + \delta_{S_j,\mathrm{me3}}\right),\ S_j \in \{\mathrm{me0},\mathrm{me1},\mathrm{me2},\mathrm{me3}\}$$

(Equation 1)

where E_i incorporates the positive feedback from neighboring H3K27me2/me3, $\rho_{\mathrm{me2}} = 0.1$ accounts for the reduced efficiency of H3K27me2-activated PRC2, and where $\delta_{i,j}$ is the Kronecker delta, equal to 1 if $i = j$ and 0 otherwise. The transition rates between methylation states $k_{\mathrm{me0-1}}$, $k_{\mathrm{me1-2}}$, $k_{\mathrm{me2-3}}$ are discussed below.

Feature 2: Transcription-Mediated PRC2 Antagonism

For the process of transcription to directly antagonize PRC2 silencing, it must cause removal of H3K27me3. In the model, this occurs in two ways: via H3K27 demethylation and histone exchange, both of which are coupled to transcription. The first is motivated by the observation that H3K27 demethylases localize to promoters and coding regions of PRC2 target genes (Chen et al., 2012; Lee et al., 2007) and can associate with transcription elongation factors (Chen et al., 2012). The second reflects the observation that histone exchange correlates positively with transcriptional activity, and negatively with Polycomb silencing (Deaton et al., 2016; Kraushaar et al., 2013) (STAR Methods). We model each passage of Pol II through the gene as a single discrete event that causes H3K27 demethylation

(B) Diagrammatic representation of feedbacks in mathematical model. States me0 to me3 refer to methylation state of H3K27. Neutral marks me0/me1 indicated in yellow, repressive marks me2/me3 in orange. Black arrows represent state transitions; colored arrows represent feedback interactions. For clarity, histone exchange and H3K27me2-mediated recruitment of PRC2 are omitted.

(C) Mathematical description of model. Sum over neighbors in E_i includes the other histone on same nucleosome, and four histones on neighboring nucleosomes. $P_{me2/me3}$ is the fraction of H3 histones carrying K27me2 or K27me3.

(D) Model parameters.

(E) Example stochastic simulation of H3K27me0 and H3K27me3 levels over time for a bistable model (initial uniform me3). Parameters indicated in (D) ($k_{\mathrm{me}} = 10^{-4}$ histone^{-1} s^{-1}, $p_{\mathrm{dem}} = 0.056$ histone^{-1} transcription^{-1}).

(F) Same as (E), for a demethylation-biased model ($k_{\mathrm{me}} = 10^{-4}$ histone^{-1} s^{-1}, $p_{\mathrm{dem}} = 0.1$ histone^{-1} transcription^{-1}).

(G) Heatmap showing bistability measure B, calculated from simulations. Each panel shows B as function of k_{me} and p_{dem}, for f_{max} shown in panel label. For each parameter set, 100 simulations were initialized in each of the uniform me0 or me3 states and simulated for 50 cell cycles. Results averaged over all simulations. In (E)–(G), $p_{\mathrm{ex}} = 10^{-3}$ histone^{-1} transcription^{-1}. See also Figures S1–S5 and Tables S1–S5.

(one methyl group at a time) and nucleosome exchange (two neighboring H3 histones with me*x*/me*x* → me0/me0), with probability p_{dem} and p_{ex} per histone, respectively (Figures 1C and 1D).

Feature 3: H3K27-Methylation-Based Transcriptional Repression

The mechanistic basis of transcriptional repression by PRC2 and H3K27me2/me3 is poorly understood. In vitro, both mammalian (Margueron et al., 2008) and *Drosophila* (Francis et al., 2004) Polycomb complexes can compact chromatin and repress transcription. Moreover, in vivo, genes enriched for H3K27me2/me3 show reduced levels of productive transcription (Brookes et al., 2012), increased chromatin compaction (Deaton et al., 2016; Eskeland et al., 2010), and deacetylated histones (Pasini et al., 2010b). To incorporate the repressive effect of PRC2 we made RNA production dependent on H3K27me2/me3 levels. We allow H3K27me2/me3 marks anywhere in the modeled region to have an equally repressive effect on transcription, with the transcriptional initiation rate *f* a simple linear function of the proportion of H3K27me2/me3 marked histones at the gene. This is appropriate if, for example, repression is mediated through compaction of chromatin at the scale of many nucleosomes (Boettiger et al., 2016; Eskeland et al., 2010). Altogether this leads to

$$f = \alpha\left(f_{max} - P_{me2/me3}\left(f_{max} - f_{min}\right)\right) \qquad \text{(Equation 2)}$$

where $P_{me2/me3}$ is the proportion of me2/me3 marks, f_{max} (f_{min}) are the maximum (minimum) transcription initiation rates, and where α is discussed below (Figures 1C and 1D).

Feature 4: Non-processivity

Methylation of H3K27 by PRC2 could be accomplished in two ways: in a processive mechanism, PRC2 would remain bound to its substrate until all three methyl groups are added, whereas in a non-processive mechanism, PRC2 would dissociate after adding each methyl group. Experimentally, it has been shown that mammalian PRC2 can monomethylate H3K27me0, H3K27me1, and H3K27me2 peptides in vitro (McCabe et al., 2012), and that in vivo, PRC2 activity is required for all H3K27me2/me3 and intragenic H3K27me1 (Ferrari et al., 2014). Furthermore, mass spectrometry has revealed that H3K27me3 is mostly formed in vivo from monomethylation of existing H3K27me2 substrates, and that H3K27me2 can arise through monomethylation of H3K27me1 (Zee et al., 2012). Collectively, these data suggest that PRC2 acts non-processively, which we therefore assume in our model. We also simulated the model with processive methylation; however, this generated only limited bistability (STAR Methods, Figure S2B). Our model also takes into account the relative catalytic activity of PRC2 on H3K27me0, me1, and me2 substrates from in vitro experiments (McCabe et al., 2012) as being 9:6:1, respectively, which is captured by the parameters k_{me0-1}, k_{me1-2}, k_{me2-3} = k_{me} in Equation 1. Noisy methylation rates, which reflect background PRC2 activity γ_{me0-1}, γ_{me1-2}, γ_{me2-3} = γ_{me} are set at 5% of the rate of allosterically activated PRC2, k_{me} (Figures 1C and 1D).

In humans, H3K27 demethylation is catalyzed by jumonji-C domain-containing proteins UTX and JMJD3 (Agger et al., 2007). To our knowledge, the processivity of H3K27 demethylation has not been investigated in vivo. However, UTX can sequentially remove single methyl groups from H3K27me3 peptides in vitro (Agger et al., 2007). The model therefore assumes non-processive demethylation, although this is not essential for bistability (STAR Methods, Figure S2). The model also includes noisy H3K27 demethylation with rate γ_{dem} (STAR Methods).

Feature 5: DNA Replication

Experiments in eukaryotes indicate that H3/H4 tetramers do not dissociate during DNA replication and are normally shared evenly between daughter chromosomes (Annunziato, 2005), maintaining their pre-replication H3K27 methylation status (Alabert et al., 2015; Gaydos et al., 2014). DNA replication occurs once per cell cycle, at which time each nucleosome in the model is replaced with a new me0/me0 nucleosome with a probability of 0.5 (Figure 1C).

The model formulated above (Figure 1) contains an important difference from previous models that include opposing activating and repressive histone modifications (Angel et al., 2011; Dodd et al., 2007). Here, DNA replication results in deposition of histone modifications associated with the active expression state, rather than an intermediate state. Hence, DNA replication only perturbs the repressed state, and actually biases the system toward the active state.

Feature 6: trans Regulators

trans-factor-mediated regulation of gene expression is encoded in our model as a multiplicative factor α in the transcription initiation rate function *f* (Equation 2). This can be interpreted as a direct, externally driven gene-activation strength, where α = 1 is neutral, $\alpha < 1$ is repressive, and $\alpha > 1$ is activating. To restrict the average transcription rate to biologically reasonable values when $\alpha \gg 1$, we also introduce an upper limit on the transcription initiation rate ($f \leq 1/60\ s^{-1}$). In our model, transcription events occur with constant probability per unit time *f*, depending on the chromatin state and *trans*-activation level α. However, for many genes, transcription occurs in bursts (reviewed in Raj and van Oudenaarden, 2008). Nevertheless, we find that a modified bursty model generates similar results to our main model (Figures S3 and S4; STAR Methods).

Together, these six features form the mathematical foundation of our model. We now proceed to analyze the model using stochastic simulations.

Chromatin States Can Store Memory of Gene Expression

For the chromatin of a PRC2 target gene to act as a memory of gene expression, it must be able to maintain both the high H3K27me3 (low expression) and low H3K27me3 (high expression) states. To investigate the ability of our model to do this, we performed stochastic simulations using the Gillespie algorithm, tracking the transcription and chromatin status of a single locus over time. At DNA replication, simulations follow only one of the two daughter loci. Figure 1E shows a simulation with parameters that maintain high H3K27me3 levels for several cell cycles, while Figure 1F shows a simulation with parameters biased toward demethylation.

When a model is capable of maintaining both active and repressed states for the same parameter values, it is bistable. Balanced bistability can be quantified as $B = 4P_{OFF}P_{ON}$ (Sneppen and Dodd, 2012), where P_{ON} (P_{OFF}) is the probability over

time that the simulated gene is in the high/ON (or low/OFF) expression state (STAR Methods). B is close to 1 for bistable models. After specifying a minimum transcription initiation rate, $f_{\min} = 10^{-4}\ s^{-1}$, a system size of 60 histones (~5–6 kb of DNA) and 22 hr cell-cycle duration, four free parameters remain in our model: k_{me}, $f_{\max}$, p_{dem}, and p_{ex}. We calculated B from simulations performed over a range of values for these four parameters (Figures 1G and S5). We find that values of B can be close to 1 (indicating *cis* epigenetic memory) if two criteria are satisfied: methylation and demethylation processes are balanced, and the increase in transcription between the active and repressed states ($F = f_{\max}/f_{\min}$) is sufficiently large (in Figure 1G, bistability emerges for $f_{\max} \geq 16 f_{\min} = 1.6 \times 10^{-3}\ s^{-1}$). For the rest of this work we set $f_{max} = 4 \times 10^{-3}\ s^{-1}$ ($F = 40$). We also find that the minimum methylation rate for which bistability is observed increases as histones are exchanged more often (Figure S5). This is because, for low methylation rates, H3K27me2/me3 is not replaced quickly enough to counteract H3K27 demethylation, histone exchange, and dilution at DNA replication. In such cases, the repressed state becomes unstable.

In summary, when H3K27 addition and removal processes are balanced, the model can exhibit bistability, demonstrating that the modeled chromatin domain can store memory of both active and repressed gene expression states.

PRC2 Target-Gene Chromatin Can Also Respond to Transcriptional Changes

After fitting our model to experimental data (Box 1, STAR Methods), we next considered the effect of directly modifying transcription on chromatin states. α represents the external *trans*-activation level of the modeled gene, with $\alpha = 1$ neutral, $\alpha > 1$ activated, and $\alpha < 1$ repressed. After initialization in either the uniform me0 or me3 state and equilibration of the model for five cell cycles with $\alpha = 1$, we permanently modified α and studied the time-evolution of H3K27 methylation. This protocol simulates recruitment of an activator or repressor that directly modulates transcriptional activity (Figures 2A and 2B).

When transcription is upregulated from an initially repressed state, the increase in polymerase traffic leads to stochastic loss of the repressed chromatin state over hours (Figure 2A). Conversely, when transcription is downregulated from an active initial state (Figure 2B), stochastic switching to the silenced state and accumulation of H3K27me3 at the population level is slow, taking several cell cycles. This is due to the slow intrinsic timescale of H3K27me3 addition. These results are reminiscent of experiments showing that accumulation of H3K27me3 occurs slowly after transcriptional shutdown (Buzas et al., 2011; Hosogane et al., 2013; Riising et al., 2014; Yuan et al., 2012). Together, these results demonstrate that chromatin states in our model can respond to sufficiently strong externally driven changes in transcription.

Our model could be modified to allow shorter pulses of *trans* activation to drive switching of chromatin states: transcription events could be made to have a stronger effect on H3K27 methylation, either by increasing p_{dem} or p_{ex}, or alternatively transcription-independent H3K27 demethylation (γ_{dem}) could be transiently increased, perhaps through *trans*-factor-mediated recruitment of H3K27-demethylases.

A Robust Window of *cis* Memory

So far, we have shown that both active and repressed expression states can be epigenetically maintained by the internal chromatin/transcription dynamics of our model (Figure 1G). This instructive mode of PRC2 activity, also known as *cis* memory, is consistent with observations of heritable silencing induced by tethering PRC2 to reporter genes in mammalian systems (Bintu et al., 2016; Hansen et al., 2008) and has been observed experimentally in *Arabidopsis* (Berry et al., 2015). We have also shown that strong external modulation of transcription in our model can cause switching between chromatin states (Figures 2A and 2B). Such a responsive mode of PRC2 activity has also been observed experimentally in mammalian cells (Gillespie and Gudas, 2007; Hosogane et al., 2013; Riising et al., 2014; Yuan et al., 2012). Taken together, this suggests that chromatin states in our model can either respond to, or instruct gene expression, depending on the strength of *trans* activation.

To further understand this interplay, and to probe the robustness of the bistable chromatin states, we simulated the model for different values of transcriptional activation α, starting from either the repressed or active initial state (after equilibration for five cell cycles at $\alpha = 1$ starting from an either uniform me3 or me0 state). After 20 cell cycles, the transcriptional output was then measured as the average number of transcription events in the final cell cycle. This is plotted as a function of α in Figure 2C (upper panel). For extreme values of α, transcriptional output is independent of the initial chromatin state, with the H3K27 methylation status being dictated entirely by *trans*-acting regulators. For a wide range of intermediate values of α (around 1), however, the transcriptional output can depend strongly on the initial state. In this regime, chromatin has a tendency to be maintained in its initial state by the internal chromatin/transcription dynamics, which therefore partly determine the transcriptional output of the gene. This intermediate range of α can be thought of as a window of *cis* memory, within which chromatin states play an instructive role in their own maintenance. However, even within this *cis* memory window, the transcriptional output of each of the bistable states can still be fine-tuned by *trans*-acting regulators. To determine how the timescale of *cis* epigenetic memory storage depends on the *trans*-activation strength, we also calculated the mean first passage time t_{FP} as a function of α for the repressed or active initial states (STAR Methods). Close to $\alpha = 1$ (within the *cis* memory window), it takes over 200 cell cycles (on average) to change from the me0 to me3 state or vice versa (Figure 2D, upper panel), again demonstrating the robustness of the bistable states. Increasing or decreasing α (simulating *trans*-activation/repression) favors the active or repressed state, respectively, leading to a reduction in the first passage time. Similar results were also obtained with a more complex model of bursty transcriptional regulation in which *trans* factors regulate the probability of a promoter switching between transcriptionally silent and active states (STAR Methods; Figures S4Q and S4R).

The ability of a gene to recruit PRC2 will depend on both its DNA sequence and also the cellular and developmental context. In our model, the local enzymatic activity and the context-specific strength of PRC2 recruitment are represented by the parameter β. To determine how changes in β affect the *cis* memory window, we performed simulations as described above, except with a 2-fold increase in the local PRC2 activity: $\beta = 2$.

OPEN
ACCESS
CellPress

Box 1. Fitting Quantitative Experimental Data Indicates that Sub-saturating H3K27me2/me3 Is Sufficient for Full Gene Repression

Nascent chromatin capture together with time-resolved stable isotope labeling by amino acids in cell culture (SILAC) was recently used to experimentally measure the dynamics of histone modification accumulation after DNA replication (Alabert et al., 2015). These data demonstrate that H3K27me3 accumulates very slowly on newly incorporated histones in dividing human somatic cells. In fact, within one cell cycle, H3K27me3 levels on newly incorporated histones do not reach the pre-replication level on parentally inherited histones. In contrast, previous mathematical models of histone-modification-based epigenetic memory have employed histone modification rates significantly faster than this, with each histone tail typically undergoing many modification reactions per cell cycle (Angel et al., 2011; Dodd et al., 2007; Sneppen and Dodd, 2012). Here we use these quantitative experimental data to constrain our model, in particular the methylation rate k_{me}. Throughout this box, we set the histone exchange rate as $p_{ex} = 10^{-3}$ histone^{-1} transcription^{-1}, a value that is justified in STAR Methods (Figure S6).

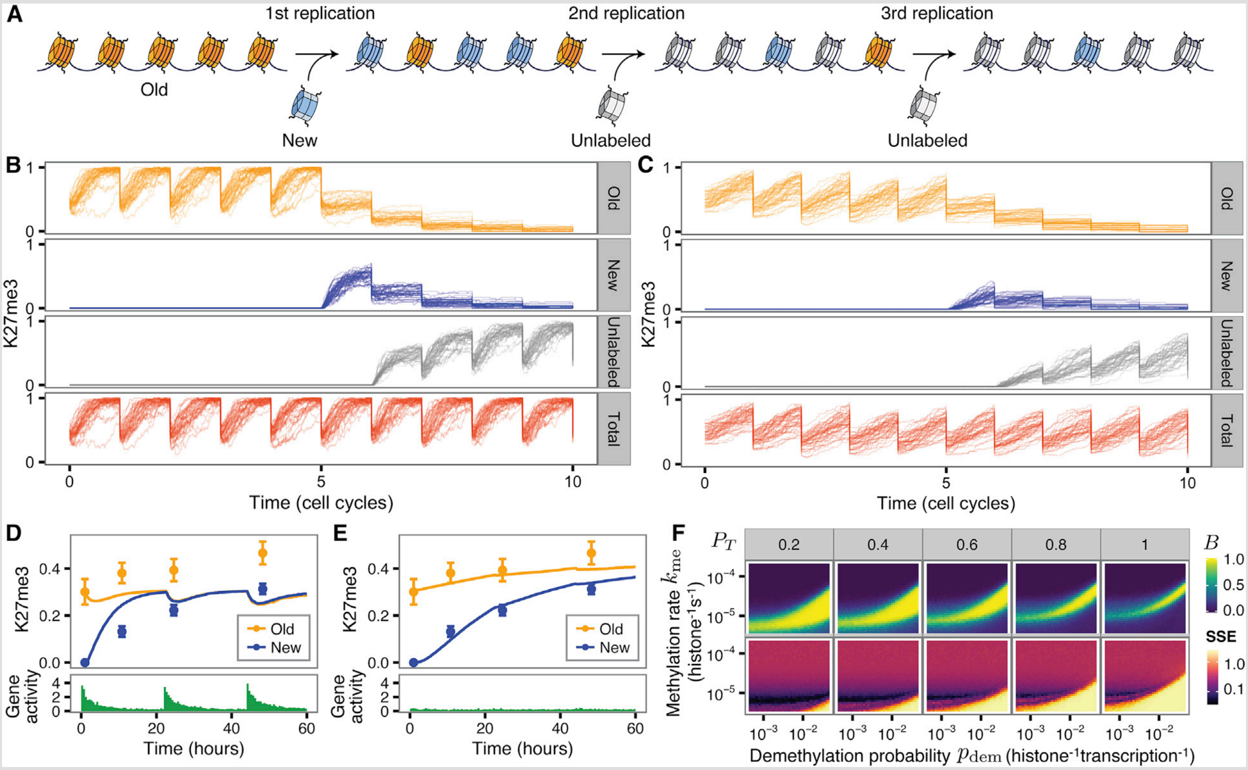

Figure B1. Fitting the Model to Experiments

(A) Schematic of SILAC experiment: old histones (yellow) diluted by incorporation of new histones (blue) at first DNA replication. Chromatin from this replication is followed through two subsequent replications, during which both old and new histones are diluted by incorporation of unlabeled histones.

(B and C) Forty over-plotted trajectories from simulated SILAC experiment. Plots show levels of K27me3-marked old, new, unlabeled, and total H3. Simulations initialized in the uniform me3 state were equilibrated for six cell cycles (five shown) before introducing new histones. (B) The slowest bistable model with $P_T = 1$ ($k_{me} = 3 \times 10^{-5}$ histone^{-1} s^{-1}, $p_{dem} = 0.02$ histone^{-1} transcription^{-1}); (C) the best-fit model with $P_T = 1/3$ ($k_{me} = 8 \times 10^{-6}$ histone^{-1} s^{-1}, $p_{dem} = 0.004$ histone^{-1} transcription^{-1}). Best-fit k_{me} obtained by minimizing the sum of squared errors (SSE) between simulated and experimental SILAC data.

(D and E). K27me3 levels on old and new H3 as a proportion of total old and new H3 incorporated, respectively. Points show experimental data from (Alabert et al., 2015), error bars: SEM (n = 3). Solid lines in (D) and (E) correspond to model simulations shown in (B) and (C), respectively. Results averaged over 1,000 simulations and normalized so that simulated mean cell-cycle end value of H3K27me3 is equal to the experimental mean initial level on old H3 (STAR Methods). Gene activity measured as number of transcription events per 30 min interval.

(F) Heatmap showing bistability measure, B (top panel) and SSE (bottom panel). Each panel shows B and SSE as a function of k_{me} and p_{dem}, for P_T shown in panel labels. B calculated from 150 simulations initialized in each of the uniform me0 or me3 states, simulated for 20 cell cycles for each parameter set. SSE calculated from 300 SILAC simulations for each parameter set. Simulations in (A)–(F) with $f_{max} = 40 f_{min}$ and $p_{ex} = 10^{-3}$ histone^{-1} transcription^{-1}, with other parameters as in Figure 1D.

The triple-SILAC experiment used to fit the model is illustrated in Figure B1A. Old histones (yellow) are distinguishable from new histones (blue) and unlabeled histones (gray). New histones are incorporated during the first DNA replication, at which time newly synthesized DNA is also labeled to allow specific isolation of this nascent chromatin at different times after the first DNA replication

(Continued on next page)

Box 1. *Continued*

(Alabert et al., 2015). Cells underwent a further two DNA replications in the 48 hr after incorporation of new histones, consistent with a 22 hr cell cycle. Accordingly, levels of both new and old histones incorporated in chromatin were diluted approximately 4-fold by incorporation of unlabeled histones. To determine whether our model could reproduce this data, we simulated this experiment (Figures B1B and B1C). Relative levels of H3K27me3 on old and new histones were extracted from model simulations 0, 10, 24, and 48 hr after new histones were first incorporated, and were quantitatively compared with corresponding experimental data (STAR Methods). Strikingly, we observed that even the model with the slowest dynamics that retained robust bistability ($k_{me} = 3 \times 10^{-5}$ histone^{-1} s^{-1} $\cong$ 2 histone^{-1} cell cycle^{-1} and p_{dem} = 0.02 histone^{-1} transcription^{-1}) was not slow enough to fit the experimental rate of H3K27me3 accumulation (Figures B1D and S7A). The problem lies in the assumption that saturating H3K27me2/me3 levels are necessary to achieve maximal gene repression (Equation 2). On such slow time scales, H3K27me2/me3 saturation is not achieved in the model within a single cell cycle. Consequently, transcription is never maximally repressed, causing an increased rate of loss of H3K27me2/me3 through transcription-coupled processes, which destabilizes the repressed state.

Experimentally, H3K27me3 levels were reduced by approximately one-half upon DNA replication, and then increased slowly with a characteristic timescale longer than a cell cycle (Alabert et al., 2015). This suggests that repressed PRC2 target genes carry K27me3 on only a fraction of their H3 histones at all stages of the cell cycle. If these H3K27me3 marks are also responsible for gene repression, then maximal repression must be achieved at sub-saturating H3K27me3 levels. We therefore introduced into the model a threshold proportion of me2/me3 marks, $P_T \leq 1$, with maximum repression above this level,

$$f = \begin{cases} \alpha\left(f_{max} - \dfrac{P_{me2/me3}}{P_T}(f_{max} - f_{min})\right), & P_{me2/me3} < P_T \\ \alpha(f_{min}), & P_{me2/me3} \geq P_T \end{cases} \qquad \text{(Equation 3)}$$

where $P_{me2/me3}$ is the proportion of me2/me3 marks (Figure 1C). Using the fixed parameter values shown in Figure 1D, simulations were performed for a range of values of P_T, k_{me}, and p_{dem} (Figures B1C, B1E, and B1F). As anticipated, including this threshold caused the region of bistability to extend to lower values of k_{me}, and encompass a larger region of parameter space (Figure B1F). For parameter values around P_T = 1/3, the model was robustly bistable at the low methylation rate required to fit the data (Figures B1F and S7B). Figures B1C and B1E show simulation results for the best-fit methylation rate for P_T = 1/3, $k_{me} = 8 \times 10^{-6}$ histone^{-1} s^{-1} (~0.6 histone^{-1} cell cycle^{-1}), with $p_{dem} = 4 \times 10^{-3}$ histone^{-1} transcription^{-1} optimized for maximum bistability. Clearly, when the threshold P_T is included, the quantitative fit to the data can be greatly improved (Figure B1E).

For all further simulations in this work, we incorporate the transcription initiation function as specified in Equation 3 with P_T = 1/3. Fitted parameters are listed in Figure S6M, and spatially resolved example simulations are provided in Figures S7C and S7D.

In summary, slow increases in H3K27me3 levels within an H3K27me3-enriched domain imply that H3K27me3 levels are not saturated throughout the cell cycle. By allowing a non-saturated H3K27me2/me3 domain to fully repress transcription, the model can maintain both active and repressed states through many cell divisions and simultaneously fit the observed slow accumulation of H3K27me3 over several cell cycles.

In this case, transcriptional output shows dependence on the initial chromatin state over an even greater range of α, and the difference in transcriptional output between the two initial states occurs at higher α values (Figure 2C). This indicates that chromatin can instruct gene expression over a wider range of transcriptional activation levels (i.e., a wider *cis* memory window). Furthermore, mean first passage times are greater within the *cis* memory window for β = 2 than for β = 1, for both initial states (Figure 2D). Therefore, the ability of chromatin to instruct gene expression can itself be quantitatively modulated through the local activity of PRC2. Other factors affecting the width of the *cis* memory window are the same as those that influence bistability, such as the number of histones in the gene, and the strength of model feedbacks (Dodd et al., 2007). In some cases, the *cis* memory window may be so narrow that chromatin is effectively always responsive to *trans* regulators.

Overall, over a wide range of external transcriptional inputs, bistable chromatin states persist, instructing their own inheritance. However, when transcription is increased or decreased beyond certain limits, beyond the *cis* memory window, bistability is abolished and the chromatin state becomes purely responsive (Figure 2E). The level of transcriptional activation or repression required to abolish bistability depends on properties such as the local PRC2 activity that may differ between PRC2 target genes and cellular contexts.

Slow Dynamics Underlies Chromatin-Based Noise Filtering

Our integrated model generates both chromatin-based epigenetic memory and *trans*-factor-mediated control of gene expression. After fitting the model to experimental SILAC data (Box 1), we found that large, persistent perturbations to external transcriptional activation are necessary to change the chromatin state (Figure 2). This suggests that chromatin may resist state changes driven by transcription and thereby buffer fluctuations in the concentration of regulatory *trans* factors.

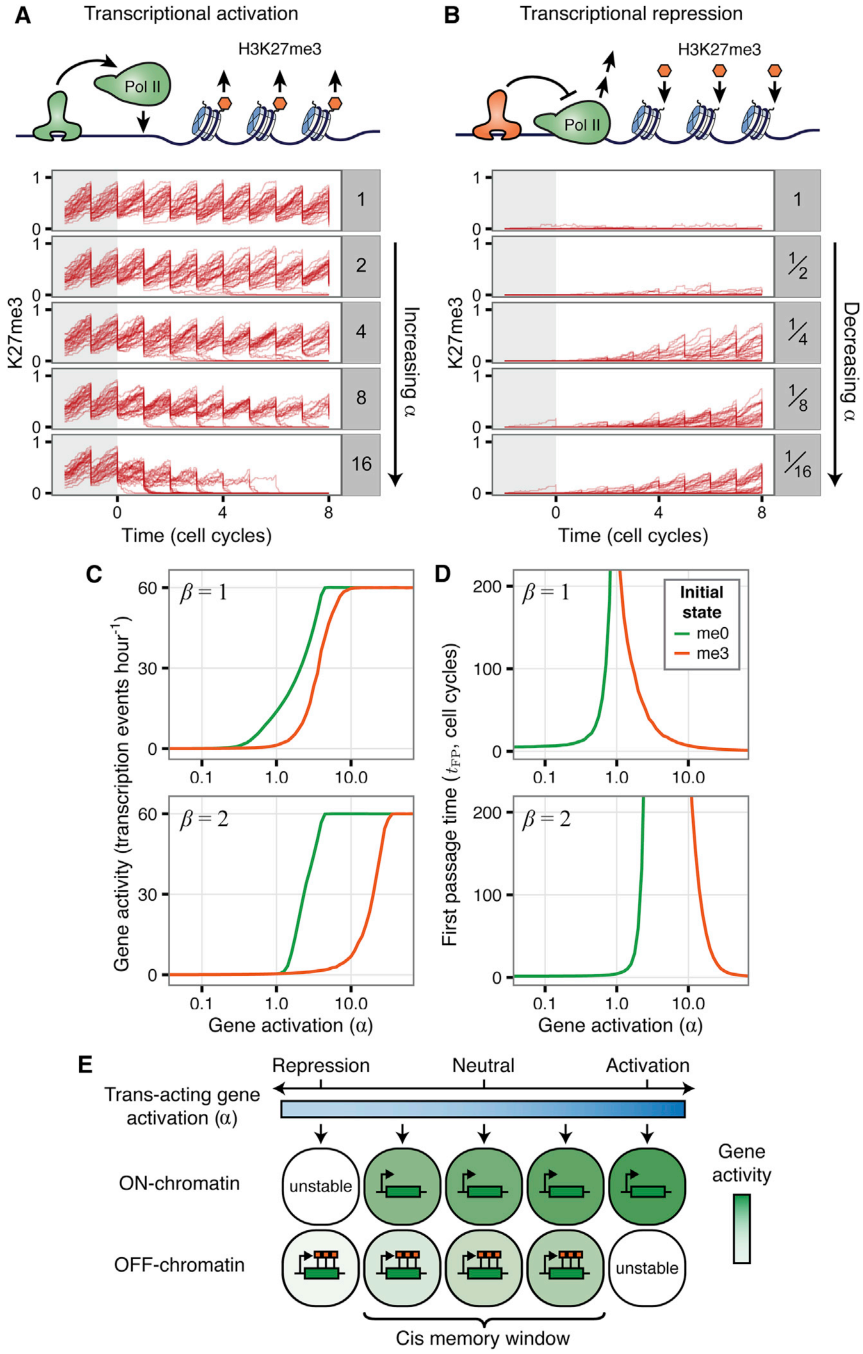

Figure 2. Integration of *cis* and *trans* Regulation

(A) Top: schematic of transcriptional activation. Below: 30 over-plotted simulated H3K27me3 time courses. After initialization in the uniform me3 state and equilibration for five cell cycles at $\alpha = 1$, α then changed to the value shown in panel label at $t = 0$. Simulations show a further eight cell cycles.

(B) Same as (A) for transcriptional repression from initial uniform me0 state.

(C) Gene activity measured as average number of transcription events (gene^{-1} hr^{-1}) in the 20th cell cycle after activation or repression, averaged over 2,000 simulations for each value of α. Green lines indicate initially active gene, orange lines indicate initially repressed gene. Upper panel: $\beta = 1$ throughout, $\alpha = 1$ during five cell-cycle equilibration, then α as indicated on x axis for further 20 cell cycles. Lower panel: $\beta = 2$ throughout, $\alpha = 5$ during five cell-cycle equilibration, then α as indicated on x axis for further 20 cell cycles.

(D) Mean first passage time, t_{FP} (STAR Methods) as function of α, averaged over 1,000 simulations each of 1,500 cell cycles, from initially active or repressed state. Model and parameters in Figure 1 (as modified by Equation 3) and Figure S6M.

(E) Schematic of the *cis* memory window. Blue shade indicates level of *trans*-acting gene activation; green shade indicates expression of PRC2 target gene. Within the window, alternative chromatin states are both stably maintained, yet gene expression levels can also be fine-tuned by *trans* regulators. Outside the window, only one chromatin state is stable. See also Figure S4.

To investigate this hypothesis, we used a stochastic model of gene expression (Ozbudak et al., 2002) to simulate a fluctuating gene-activation function, $\alpha(t)$ (STAR Methods). The noisiness of this input signal is measured from simulations as the coefficient of variation of $\alpha(t)$. In simulations, the size of fluctuations can be modulated without affecting the mean (i.e., $\langle \alpha(t) \rangle = 1$, where $\langle\rangle$ indicates a time average). Although the methylation rate k_{me} was constrained using experimental data (Box 1, Figure B1E), we now allow this parameter to vary in order to understand how its value influences the noise-filtering capability of this system. With input functions of various noise strengths, we performed simulations over a range of k_{me} and p_{dem}. From these simulations, we calculated the combined first passage time, *FP*, which quantifies the ability of the model to maintain both active and repressed states (STAR Methods). *FP* ranges from 0 to 1, with larger values indicating greater average state lifetimes.

Strikingly, we observed that systems with fast dynamics (high k_{me}, high p_{dem}) that were bistable ($FP \approx 1$) when noise was low showed a marked decrease in *FP*, indicating weakened bistability as noise was increased (Figures 3A and S8A). Conversely, bistable models with slower dynamics were better able to maintain long chromatin state lifetimes (high *FP*) as noise in the input signal was increased. Example simulations are shown in Figures 3B–3E and S8B–S8E. We observed that the model with the methylation rate obtained from fitting the SILAC data ($k_{me} = 8 \times 10^{-6}$ histone^{-1} s^{-1}, ~0.6 histone^{-1} cell cycle^{-1}) also showed greater bistability than systems with even slower dynamics (Figure 3A) regardless of the noise strength. This is due to an inability of the slower models to counteract the loss of H3K27me2/me3 that occurs at DNA replication.

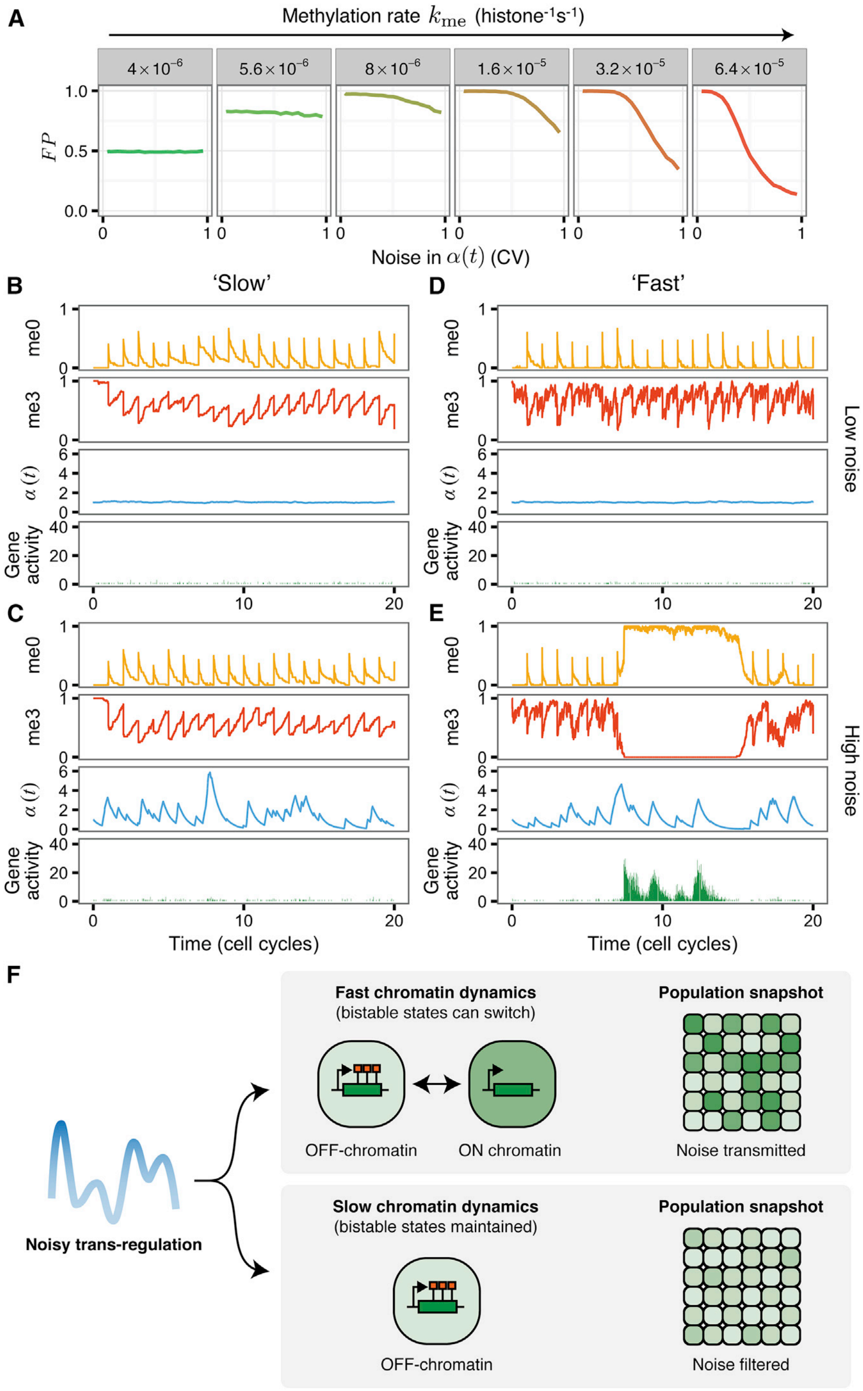

Figure 3. Slow H3K27 Methylation Dynamics Generate Robustness to Noise

(A) First passage time measure, *FP*, as a function of noise in the gene-activation input signal $\alpha(t)$. Noise measured as coefficient of variation (CV) in $\alpha(t)$. For each parameter set, 3,000 simulations were initialized in each of the uniform me0 or me3 states and simulated for 20 cell cycles. *FP* calculated as described in STAR Methods. Each panel shows results for k_{me} value in panel label. For each k_{me}, p_{dem} was chosen to maximize *FP* for constant $\alpha(t) = 1$. From left to right, p_{dem} values: 0.001, 0.001, 0.004, 0.03, 0.07, and 0.1 histone^{-1} transcription^{-1}. Results over larger parameter space shown in Figure S8A.

(B–E) Example simulations initialized in repressed (uniform me3) state with variable transcriptional activation signals $\alpha(t)$. $\alpha(t)$ has low noise (CV $\approx$ 0) in (B) and (D), and high noise (CV $\approx$ 1) in (C) and (E). (B) and (C) show slow dynamics (k_{me} = 8 $\times$ 10^{-6} histone^{-1} s^{-1}, p_{dem} = 4 $\times$ 10^{-3} histone^{-1} transcription^{-1}). (D) and (E) show fast dynamics (k_{me} = 4 $\times$ 10^{-5} histone^{-1} s^{-1}, p_{dem} = 2 $\times$ 10^{-1} histone^{-1} transcription^{-1}). Model and other parameters in Figure 1 (as modified by Equation 3) and Figure S6M (β = 1). Gene activity measured as number of transcription events per 30 min interval. Similar plots with active initial states shown in Figures S8B–S8E.

(F) Schematic illustrating filtering of noise in gene-activation signals. Blue shading indicates level of *trans*-acting gene activation; green shading indicates expression of PRC2 target gene. Fast chromatin dynamics: chromatin rapidly responds to transient pulses of activation or repression causing switching between alternative chromatin states over time, and heterogeneous expression levels in a population. Slow chromatin dynamics: transient pulses of activation are not sufficient to activate the chromatin state, resulting in lower uniform expression of the PRC2 target gene. See also Figure S8.

The model therefore suggests a rationale for why experimental H3K27me3 accumulation is slow: genes that change H3K27me3 levels slowly in response to varying *trans*-factor inputs offer more stable memory storage than genes with faster chromatin dynamics because neither prolonged absences nor pulses of transcriptional regulators are sufficient to change chromatin states. Interestingly, a previous study of mammalian heterochromatin also used modeling to suggest that fluctuations of chromatin regulators on shorter timescales (minutes) would not perturb H3K9 methylation status (Muller-Ott et al., 2014). In contrast to our model, however, the heterochromatin model was monostable.

DISCUSSION

In this work, we have introduced a mathematical model which mechanistically integrates transcription and chromatin-based epigenetic regulation. The model exhibits bistable *cis* epigenetic memory over a wide range of parameter values and is able to quantitatively reproduce the slow H3K27me3 accumulation rates observed in vivo (Box 1). When dynamics are slow, we also find that chromatin of PRC2 targets can effectively ignore transient pulses of activation or repression so that fluctuations in levels of *trans* regulators do not lead to loss of *cis* epigenetic memory (Figure 3F). Fundamentally, these results rest on two main features: transcription antagonizing chromatin silencing, and *cis*-acting positive feedbacks maintaining repressive histone modifications. Thus, the

concepts we have highlighted may be widely applicable, e.g., to heterochromatic H3K9 methylation in *S. pombe* (Kowalik et al., 2015).

Many PRC2 target genes are under the control of gene-regulatory networks and would therefore seem to have no need for PRC2 in maintenance of epigenetic memory. This observation has led to questions regarding the function of PRC2 in such cases (Ringrose, 2007). The ability to filter noise may explain why PRC2 is repeatedly employed in gene-regulatory networks, sometimes acting as a short-term rather than long-term memory. Given that many transcription factors are themselves PRC2 targets, such noise filtering at the transcriptional level may endow regulatory networks with greatly increased robustness. The machinery required for chromatin-based noise filtering is generic and can act simultaneously at many different genomic loci, and may therefore be regarded as an example of passive noise filtering (Stoeger et al., 2016).

Previous theoretical models of histone-modification-based epigenetics found that bistability requires modified histones to recruit enzymatic complexes that act beyond neighboring nucleosomes (Dodd et al., 2007). These long-range interactions are attributed to DNA looping, which bring together nucleosomes that are distant in the one-dimensional chromatin fiber. Intuitively, long-range interactions ensure that a set of histones within an individual domain coordinate their modification status, preventing the formation of stable sub-domains of opposing activating and repressive modifications. However, preventing such models from exhibiting uncontrolled spreading to nearby genomic loci is problematic (Dodd and Sneppen, 2011). In contrast to such long-range interactions, our model requires only local interactions between histones and their modifying complexes, where PRC2 recruited to one nucleosome only acts on its immediately neighboring nucleosomes. The reason that bistability is still observed in this model is two-fold. First, the model contains no locally self-reinforcing opposing mark, so the problem of an opposing mark invading a repressed domain does not exist. Second, although histone modifications recruit complexes that act only on neighboring nucleosomes, the opposing state of transcription can act anywhere within the gene. This effectively generates a demethylation rate that is determined by the average chromatin state of the entire gene. In this sense, the process of transcription and the mechanism by which it is regulated by H3K27me2/me3 fulfill the requirement for long-range interactions. Nevertheless, our model has advantages over models with explicit long-range action of histone modifiers. First, the chromatin state of the entire gene is naturally coordinated by the process of transcription. Second, the DNA sequence used to control the initiation and termination of transcription can also be used to naturally define the boundaries of chromatin activation. It is also possible that the rare transcriptional events that occur in the repressed state could help in specifying the boundaries of H3K27me3 domains. Moreover, unlike models with long-range interactions between histone modifiers, spreading of repressive chromatin in our model is strictly one-dimensional; along the chromatin fiber. This means that H3K27me3 could also be prevented from spreading by one-dimensional insulator elements consisting of nucleosome-depleted regions, regions of high histone exchange (such as actively transcribed regions), or histones that are somehow refractory to H3K27-methylation.

The model developed in this work fundamentally integrates bistable *cis*-acting epigenetic memory with *trans*-acting transcriptional control. One key difference between these two regulatory modes is that the chromatin states are digital (on/off), whereas *trans* regulators can act in an analog manner, with transcriptional output depending continuously on the concentrations of the regulators (Giorgetti et al., 2010). The concepts of digital and analog regulation provide an alternative way of thinking about the results of our model: within the *cis* memory window, bistable (digital) chromatin states persist (instructing their own inheritance). However, the expression levels of these digital chromatin states can be fine-tuned in a continuous analog way by the activity of *trans* regulators (Figure 2E). In this way, our model exhibits a fusion of digital and analog transcriptional control.

Experimental Outlook

Our model makes two further specific predictions that are experimentally testable. First, the model predicts that for each PRC2 target there is an upper threshold of *trans* activation above which chromatin-based repression cannot be established; a lower threshold below which chromatin-based repression is guaranteed; and an intermediate range of *trans*-activation strengths over which the chromatin state instructs its own inheritance and contributes to determining gene expression. Understanding how these thresholds depend on various features of PRC2 target-gene sequence and chromatin features will be essential in understanding genome-wide functions of PRC2. Second, the model predicts that slow chromatin dynamics allow PRC2 target genes to filter noise in *trans* regulators.

Monitoring gene expression at the single-cell level while dynamically tethering PRC2 and other chromatin modifiers has recently been used in a synthetic system to reveal that chromatin silencing is generally an all-or-none phenomenon (Bintu et al., 2016), in agreement with results from naturally occurring Polycomb systems (Berry et al., 2015). Using similar synthetic approaches, one could combine dynamic recruitment of chromatin modifiers with simultaneous quantitative modulation of transcription. This would enable detailed mechanistic dissection of the interplay between transcription and PRC2 activity. In such an experimental system, the prediction of noise filtering could also be explicitly tested by providing pulses of *trans* activation of different strengths and durations.

Inducible tethering of transcriptional activators and chromatin modifiers (Gilbert et al., 2014) could also be used at endogenous PRC2 targets, and should enable quantitative comparisons of the memory-storage capabilities of different PRC2 targets, or the same target in different cellular contexts. Similar to our previous experimental work (Berry et al., 2015), assays with single-cell resolution and an ability to trace cell lineages will be essential.

STAR★METHODS

Detailed methods are provided in the online version of this paper and include the following:

- KEY RESOURCES TABLE
- CONTACT FOR REAGENT AND RESOURCE SHARING

- METHOD DETAILS
  - Computational Methods and Simulation Details
  - Two-State Model
  - Processivity in Methylation or Demethylation
  - Transcriptional Bursting
  - Additional Details of the Main Model
  - Fitting Triple-SILAC Mass Spectrometry Data
  - Stochastic Model of a Noisy Transcriptional Regulator

SUPPLEMENTAL INFORMATION

Supplemental Information includes eight figures and five tables and can be found with this article online at http://dx.doi.org/10.1016/j.cels.2017.02.013.

AUTHOR CONTRIBUTIONS

S.B. and M.H. conceived the study and constructed the model. S.B. performed simulations and analyzed results. S.B., C.D., and M.H. wrote the manuscript.

ACKNOWLEDGMENTS

We thank Anja Groth and Axel Imhof for the SILAC data, Leonie Ringrose and Carsten Marr for helpful discussions and comments on the manuscript, and all members of the Howard and Dean groups for discussions. Research was supported by an Advanced Investigator European Research Council grant MEXTIM (to S.B., C.D., and M.H.), by grant BB/J004588/1 from the Biotechnology and Biological Sciences Research Council (to S.B., C.D., and M.H.) and by a John Innes Foundation Rotation PhD Studentship (to S.B).

Received: July 7, 2016
Revised: October 10, 2016
Accepted: February 27, 2017
Published: March 22, 2017

REFERENCES

Agger, K., Cloos, P.A.C., Christensen, J., Pasini, D., Rose, S., Rappsilber, J., Issaeva, I., Canaani, E., Salcini, A.E., and Helin, K. (2007). UTX and JMJD3 are histone H3K27 demethylases involved in HOX gene regulation and development. Nature *449*, 731–734.

Alabert, C., Barth, T.K., Reverón-Gómez, N., Sidoli, S., Schmidt, A., Jensen, O.N., Imhof, A., and Groth, A. (2015). Two distinct modes for propagation of histone PTMs across the cell cycle. Genes Dev. *29*, 585–590.

Angel, A., Song, J., Dean, C., and Howard, M. (2011). A Polycomb-based switch underlying quantitative epigenetic memory. Nature *476*, 105–108.

Annunziato, A.T. (2005). Split decision: what happens to nucleosomes during DNA replication? J. Biol. Chem. *280*, 12065–12068.

Bauer, M., Trupke, J., and Ringrose, L. (2016). The quest for mammalian Polycomb response elements: are we there yet? Chromosoma *125*, 471–496.

Berry, S., Hartley, M., Olsson, T.S.G., Dean, C., and Howard, M. (2015). Local chromatin environment of a Polycomb target gene instructs its own epigenetic inheritance. Elife *4*, e07205.

Bintu, L., Yong, J., Antebi, Y.E., McCue, K., Kazuki, Y., Uno, N., Oshimura, M., and Elowitz, M.B. (2016). Dynamics of epigenetic regulation at the single-cell level. Science *351*, 720–724.

Boettiger, A.N., Bintu, B., Moffitt, J.R., Wang, S., Beliveau, B.J., Fudenberg, G., Imakaev, M., Mirny, L.A., Wu, C.-T., and Zhuang, X. (2016). Super-resolution imaging reveals distinct chromatin folding for different epigenetic states. Nature *529*, 418–422.

Bratsun, D., Volfson, D., Tsimring, L.S., and Hasty, J. (2005). Delay-induced stochastic oscillations in gene regulation. Proc. Natl. Acad. Sci. USA *102*, 14593–14598.

Brookes, E., de Santiago, I., Hebenstreit, D., Morris, K.J., Carroll, T., Xie, S.Q., Stock, J.K., Heidemann, M., Eick, D., Nozaki, N., et al. (2012). Polycomb associates genome-wide with a specific RNA polymerase II variant, and regulates metabolic genes in ESCs. Cell Stem Cell *10*, 157–170.

Buzas, D.M., Robertson, M., Finnegan, E.J., and Helliwell, C.A. (2011). Transcription-dependence of histone H3 lysine 27 trimethylation at the Arabidopsis polycomb target gene FLC. Plant J. *65*, 872–881.

Cao, R., Wang, L., Wang, H., Xia, L., Erdjument-Bromage, H., Tempst, P., Jones, R.S., and Zhang, Y. (2002). Role of histone H3 lysine 27 methylation in Polycomb-group silencing. Science *298*, 1039–1043.

Chen, S., Ma, J., Wu, F., Xiong, L.-J., Ma, H., Xu, W., Lv, R., Li, X., Villén, J., Gygi, S.P., et al. (2012). The histone H3 Lys 27 demethylase JMJD3 regulates gene expression by impacting transcriptional elongation. Genes Dev. *26*, 1364–1375.

Chin, H.G., Patnaik, D., Estève, P.-O., Jacobsen, S.E., and Pradhan, S. (2006). Catalytic properties and kinetic mechanism of human recombinant Lys-9 histone H3 methyltransferase suv39h1: participation of the chromodomain in enzymatic catalysis. Biochemistry *45*, 3272–3284.

Dar, R.D., Razooky, B.S., Singh, A., Trimeloni, T.V., McCollum, J.M., Cox, C.D., Simpson, M.L., and Weinberger, L.S. (2012). Transcriptional burst frequency and burst size are equally modulated across the human genome. Proc. Natl. Acad. Sci. USA *109*, 17454–17459.

Deal, R.B., Henikoff, J.G., and Henikoff, S. (2010). Genome-wide kinetics of nucleosome turnover determined by metabolic labeling of histones. Science *328*, 1161–1164.

Deaton, A.M., Gómez-Rodríguez, M., Mieczkowski, J., Tolstorukov, M.Y., Kundu, S., Sadreyev, R.I., Jansen, L.E., and Kingston, R.E. (2016). Enhancer regions show high histone H3.3 turnover that changes during differentiation. Elife *5*, e15316.

Dion, M.F., Kaplan, T., Kim, M., Buratowski, S., Friedman, N., and Rando, O.J. (2007). Dynamics of replication-independent histone turnover in budding yeast. Science *315*, 1405–1408.

Dodd, I.B., and Sneppen, K. (2011). Barriers and silencers: a theoretical toolkit for control and containment of nucleosome-based epigenetic states. J. Mol. Biol. *414*, 624–637.

Dodd, I.B., Micheelsen, M.A., Sneppen, K., and Thon, G. (2007). Theoretical analysis of epigenetic cell memory by nucleosome modification. Cell *129*, 813–822.

Erokhin, M., Elizar'ev, P., Parshikov, A., Schedl, P., Georgiev, P., and Chetverina, D. (2015). Transcriptional read-through is not sufficient to induce an epigenetic switch in the silencing activity of Polycomb response elements. Proc. Natl. Acad. Sci. USA *112*, 14930–14935.

Eskeland, R., Leeb, M., Grimes, G.R., Kress, C., Boyle, S., Sproul, D., Gilbert, N., Fan, Y., Skoultchi, A.I., Wutz, A., et al. (2010). Ring1B compacts chromatin structure and represses gene expression independent of histone ubiquitination. Mol. Cell *38*, 452–464.

Ferrari, K.J., Scelfo, A., Jammula, S., Cuomo, A., Barozzi, I., Stützer, A., Fischle, W., Bonaldi, T., and Pasini, D. (2014). Polycomb-dependent H3K27me1 and H3K27me2 regulate active transcription and enhancer fidelity. Mol. Cell *53*, 49–62.

Fonseca, J.P., Steffen, P.A., Müller, S., Lu, J., Sawicka, A., Seiser, C., and Ringrose, L. (2012). In vivo Polycomb kinetics and mitotic chromatin binding distinguish stem cells from differentiated cells. Genes Dev. *26*, 857–871.

Francis, N.J., Kingston, R.E., and Woodcock, C.L. (2004). Chromatin compaction by a polycomb group protein complex. Science *306*, 1574–1577.

Gaydos, L.J., Rechtsteiner, A., Egelhofer, T.A., Carroll, C.R., and Strome, S. (2012). Antagonism between MES-4 and Polycomb repressive complex 2 promotes appropriate gene expression in *C. elegans* germ cells. Cell Rep. *2*, 1169–1177.

Gaydos, L.J., Wang, W., and Strome, S. (2014). H3K27me and PRC2 transmit a memory of repression across generations and during development. Science *345*, 1515–1518.

Gilbert, L.A., Horlbeck, M.A., Adamson, B., Villalta, J.E., Chen, Y., Whitehead, E.H., Guimaraes, C., Panning, B., Ploegh, H.L., Bassik, M.C., et al. (2014). Genome-scale CRISPR-mediated control of gene repression and activation. Cell *159*, 647–661.

Gillespie, D.T. (1977). Exact stochastic simulation of coupled chemical reactions. J. Phys. Chem. *81*, 2340–2361.

Gillespie, R.F., and Gudas, L.J. (2007). Retinoid regulated association of transcriptional co-regulators and the polycomb group protein SUZ12 with the retinoic acid response elements of Hoxa1, RARbeta(2), and Cyp26A1 in F9 embryonal carcinoma cells. J. Mol. Biol. *372*, 298–316.

Giorgetti, L., Siggers, T., Tiana, G., Caprara, G., Notarbartolo, S., Corona, T., Pasparakis, M., Milani, P., Bulyk, M.L., and Natoli, G. (2010). Noncooperative interactions between transcription factors and clustered DNA binding sites enable graded transcriptional responses to environmental inputs. Mol. Cell *37*, 418–428.

Hansen, K.H., Bracken, A.P., Pasini, D., Dietrich, N., Gehani, S.S., Monrad, A., Rappsilber, J., Lerdrup, M., and Helin, K. (2008). A model for transmission of the H3K27me3 epigenetic mark. Nat. Cell Biol. *10*, 1291–1300.

Hosogane, M., Funayama, R., Nishida, Y., Nagashima, T., and Nakayama, K. (2013). Ras-induced changes in H3K27me3 occur after those in transcriptional activity. PLoS Genet. *9*, e1003698.

Jamai, A., Imoberdorf, R.M., and Strubin, M. (2007). Continuous histone H2B and transcription-dependent histone H3 exchange in yeast cells outside of replication. Mol. Cell *25*, 345–355.

Jin, C., Zang, C., Wei, G., Cui, K., Peng, W., Zhao, K., and Felsenfeld, G. (2009). H3.3/H2A.Z double variant-containing nucleosomes mark "nucleosome-free regions" of active promoters and other regulatory regions. Nat. Genet. *41*, 941–945.

Kimura, H., and Cook, P.R. (2001). Kinetics of core histones in living human cells: little exchange of H3 and H4 and some rapid exchange of H2B. J. Cell Biol. *153*, 1341–1353.

Klymenko, T., and Müller, J. (2004). The histone methyltransferases Trithorax and Ash1 prevent transcriptional silencing by Polycomb group proteins. EMBO Rep. *5*, 373–377.

Kowalik, K.M., Shimada, Y., Flury, V., Stadler, M.B., Batki, J., and Bühler, M. (2015). The Paf1 complex represses small-RNA-mediated epigenetic gene silencing. Nature *520*, 248–252.

Kraushaar, D.C., Jin, W., Maunakea, A., Abraham, B., Ha, M., and Zhao, K. (2013). Genome-wide incorporation dynamics reveal distinct categories of turnover for the histone variant H3.3. Genome Biol. *14*, R121.

Kulaeva, O.I., Hsieh, F.-K., Chang, H.-W., Luse, D.S., and Studitsky, V.M. (2013). Mechanism of transcription through a nucleosome by RNA polymerase II. Biochim. Biophys. Acta *1829*, 76–83.

Kuzmichev, A., Nishioka, K., Erdjument-Bromage, H., Tempst, P., and Reinberg, D. (2002). Histone methyltransferase activity associated with a human multiprotein complex containing the Enhancer of Zeste protein. Genes Dev. *16*, 2893–2905.

Lee, M.G., Villa, R., Trojer, P., Norman, J., Yan, K.-P., Reinberg, D., Di Croce, L., and Shiekhattar, R. (2007). Demethylation of H3K27 regulates polycomb recruitment and H2A ubiquitination. Science *318*, 447–450.

Margueron, R., Li, G., Sarma, K., Blais, A., Zavadil, J., Woodcock, C.L., Dynlacht, B.D., and Reinberg, D. (2008). Ezh1 and Ezh2 maintain repressive chromatin through different mechanisms. Mol. Cell *32*, 503–518.

Margueron, R., Justin, N., Ohno, K., Sharpe, M.L., Son, J., Drury, W.J.I., Voigt, P., Martin, S.R., Taylor, W.R., De Marco, V., et al. (2009). Role of the polycomb protein EED in the propagation of repressive histone marks. Nature *461*, 762–767.

Matsumoto, M., and Nishimura, T. (1998). Mersenne twister: a 623-dimensionally equidistributed uniform pseudo-random number generator. ACM Trans. Model. Comput. Simul. *8*, 3–30.

McCabe, M.T., Graves, A.P., Ganji, G., Diaz, E., Halsey, W.S., Jiang, Y., Smitheman, K.N., Ott, H.M., Pappalardi, M.B., Allen, K.E., et al. (2012). Mutation of A677 in histone methyltransferase EZH2 in human B-cell lymphoma promotes hypertrimethylation of histone H3 on lysine 27 (H3K27). Proc. Natl. Acad. Sci. USA *109*, 2989–2994.

Mikkelsen, T.S., Ku, M., Jaffe, D.B., Issac, B., Lieberman, E., Giannoukos, G., Alvarez, P., Brockman, W., Kim, T.-K., Koche, R.P., et al. (2007). Genome-wide maps of chromatin state in pluripotent and lineage-committed cells. Nature *448*, 553–560.

Moazed, D. (2011). Mechanisms for the inheritance of chromatin states. Cell *146*, 510–518.

Molina, N., Suter, D.M., Cannavo, R., Zoller, B., Gotic, I., and Naef, F. (2013). Stimulus-induced modulation of transcriptional bursting in a single mammalian gene. Proc. Natl. Acad. Sci. USA *110*, 20563–20568.

Muller-Ott, K., Erdel, F., Matveeva, A., Mallm, J.P., Rademacher, A., Hahn, M., Bauer, C., Zhang, Q., Kaltofen, S., Schotta, G., et al. (2014). Specificity, propagation, and memory of pericentric heterochromatin. Mol. Syst. Biol. *10*, 746.

Ozbudak, E.M., Thattai, M., Kurtser, I., Grossman, A.D., and van Oudenaarden, A. (2002). Regulation of noise in the expression of a single gene. Nat. Genet. *31*, 69–73.

Pasini, D., Cloos, P.A.C., Walfridsson, J., Olsson, L., Bukowski, J.-P., Johansen, J.V., Bak, M., Tommerup, N., Rappsilber, J., and Helin, K. (2010a). JARID2 regulates binding of the Polycomb repressive complex 2 to target genes in ES cells. Nature *464*, 306–310.

Pasini, D., Malatesta, M., Jung, H.R., Walfridsson, J., Willer, A., Olsson, L., Skotte, J., Wutz, A., Porse, B., Jensen, O.N., et al. (2010b). Characterization of an antagonistic switch between histone H3 lysine 27 methylation and acetylation in the transcriptional regulation of Polycomb group target genes. Nucl. Acids Res. *38*, 4958–4969.

Patnaik, D., Chin, H.G., Estève, P.-O., Benner, J., Jacobsen, S.E., and Pradhan, S. (2004). Substrate specificity and kinetic mechanism of mammalian G9a histone H3 methyltransferase. J. Biol. Chem. *279*, 53248–53258.

Paulsson, J. (2005). Models of stochastic gene expression. Phys. Life Rev. *2*, 157–175.

Peccoud, J., and Ycart, B. (1995). Markovian modeling of gene-product synthesis. Theor. Popul. Biol. *48*, 222–234.

Pengelly, A.R., Copur, Ö., Jäckle, H., Herzig, A., and Müller, J. (2013). A histone mutant reproduces the phenotype caused by loss of histone-modifying factor Polycomb. Science *339*, 698–699.

Petruk, S., Sedkov, Y., Smith, S., Tillib, S., Kraevski, V., Nakamura, T., Canaani, E., Croce, C.M., and Mazo, A. (2001). Trithorax and dCBP acting in a complex to maintain expression of a homeotic gene. Science *294*, 1331–1334.

Raj, A., and van Oudenaarden, A. (2008). Nature, nurture, or chance: stochastic gene expression and its consequences. Cell *135*, 216–226.

Raj, A., Peskin, C.S., Tranchina, D., Vargas, D.Y., and Tyagi, S. (2006). Stochastic mRNA synthesis in mammalian cells. PLoS Biol. *4*, e309.

Ray-Gallet, D., Woolfe, A., Vassias, I., Pellentz, C., Lacoste, N., Puri, A., Schultz, D.C., Pchelintsev, N.A., Adams, P.D., Jansen, L.E.T., et al. (2011). Dynamics of histone H3 deposition in vivo reveal a nucleosome gap-filling mechanism for H3.3 to maintain chromatin integrity. Mol. Cell *44*, 928–941.

Riising, E.M., Comet, I., Leblanc, B., Wu, X., Johansen, J.V., and Helin, K. (2014). Gene silencing triggers polycomb repressive complex 2 recruitment to CpG islands genome wide. Mol. Cell *55*, 347–360.

Ringrose, L. (2007). Polycomb comes of age: genome-wide profiling of target sites. Curr. Opin. Cell Biol. *19*, 290–297.

Senecal, A., Munsky, B., Proux, F., Ly, N., Braye, F.E., Zimmer, C., Mueller, F., and Darzacq, X. (2014). Transcription factors modulate c-Fos transcriptional bursts. Cell Rep. *8*, 75–83.

Skinner, S.O., Xu, H., Nagarkar-Jaiswal, S., Freire, P.R., Zwaka, T.P., and Golding, I. (2016). Single-cell analysis of transcription kinetics across the cell cycle. Elife *5*, e59928.

Sneppen, K., and Dodd, I.B. (2012). A simple histone code opens many paths to epigenetics. PLoS Comput. Biol. *8*, e1002643.

Spencer, C.A., Kruhlak, M.J., Jenkins, H.L., Sun, X., and Bazett-Jones, D.P. (2000). Mitotic transcription repression in vivo in the absence of nucleosomal chromatin condensation. J. Cell Biol. *150*, 13–26.

Stoeger, T., Battich, N., and Pelkmans, L. (2016). Passive noise filtering by cellular compartmentalization. Cell *164*, 1151–1161.

Suter, D.M., Molina, N., Gatfield, D., Schneider, K., Schibler, U., and Naef, F. (2011). Mammalian genes are transcribed with widely different bursting kinetics. Science *332*, 472–474.

Tagami, H., Ray-Gallet, D., Almouzni, G., and Nakatani, Y. (2004). Histone H3.1 and H3.3 complexes mediate nucleosome assembly pathways dependent or independent of DNA synthesis. Cell *116*, 51–61.

Tie, F., Banerjee, R., Saiakhova, A.R., Howard, B., Monteith, K.E., Scacheri, P.C., Cosgrove, M.S., and Harte, P.J. (2014). Trithorax monomethylates histone H3K4 and interacts directly with CBP to promote H3K27 acetylation and antagonize Polycomb silencing. Development *141*, 1129–1139.

Venkatesh, S., and Workman, J.L. (2015). Histone exchange, chromatin structure and the regulation of transcription. Nat. Rev. Mol. Cell Biol. *16*, 178–189.

Yuan, W., Xu, M., Huang, C., Liu, N., Chen, S., and Zhu, B. (2011). H3K36 methylation antagonizes PRC2-mediated H3K27 methylation. J. Biol. Chem. *286*, 7983–7989.

Yuan, W., Wu, T., Fu, H., Dai, C., Wu, H., Liu, N., Li, X., Xu, M., Zhang, Z., Niu, T., et al. (2012). Dense chromatin activates Polycomb repressive complex 2 to regulate H3 lysine 27 methylation. Science *337*, 971–975.

Zee, B.M., Britton, L.-M.P., Wolle, D., Haberman, D.M., and Garcia, B.A. (2012). Origins and formation of histone methylation across the human cell cycle. Mol. Cell. Biol. *32*, 2503–2514.

Zentner, G.E., and Henikoff, S. (2013). Regulation of nucleosome dynamics by histone modifications. Nat. Struct. Mol. Biol. *20*, 259–266.

STAR★METHODS

KEY RESOURCES TABLE

REAGENT or RESOURCE	SOURCE	IDENTIFIER
Deposited Data		
SILAC histone mass spectrometry data	(Alabert et al., 2015)	N/A
Software and Algorithms		
Gillespie's stochastic simulation algorithm	(Gillespie, 1977)	N/A

CONTACT FOR REAGENT AND RESOURCE SHARING

Further information and requests for resources and reagents should be directed to and will be fulfilled by the Lead Contact, Martin Howard (martin.howard@jic.ac.uk).

METHOD DETAILS

Computational Methods and Simulation Details

Programming Languages and Computing Resources

All simulations were written in C and compiled using GCC (version 4.4.7). Pseudo-random numbers were generated in the GNU scientific library (GSL, version 1.13) random number environment using the Mersenne Twister 19937 algorithm (Matsumoto and Nishimura, 1998). The seed was either specified manually (for code development and simulating specific trajectories) or set based on the system clock using the time function of the C standard library. Simulations were run on the Howard group cluster, which comprises 4 compute nodes, each equipped with 16-core Xeon E5-2650 processors, running at 2.6 GHz, with 16 GB of system memory. The cluster runs the CentOS 6.6 distribution of the Linux operating system.

Mathematical Modeling of Chromatin

Stochastic simulations of H3K27 methylation, demethylation and transcription were simulated according to the 'direct' Gillespie algorithm (Gillespie, 1977). The algorithm is completely defined by a set of possible state transitions (reactions), and a corresponding propensity for each of the reactions to occur. At each iteration, the time-step Δt and the next reaction are selected probabilistically. The selected reaction is then performed by updating the system state, and system time is incremented by Δt.

In our simulations, we explicitly track the methylation status, S_i of each H3 histone $i \in [1, N]$ within a simulated region of chromatin ($S_i \in \{\text{me0, me1, me2, me3}\}$). Since we are considering methylation of H3K27, in the following we refer to H3 histones simply as histones. Each nucleosome consists of a pair of histones, (k, k+1) for odd numbers k such that $1 \le k \le N-1$, with N even. Methylation and demethylation reactions increase or decrease by one, respectively, the number of methyl groups at histone i. Initiation of transcription is also modelled as a reaction. Therefore, for a system of N histones there are a total of $2N+1$ possible reactions (N histone methylations, N histone demethylations and transcription). However, not all reactions are possible at all times, e.g. methylation of me3 histones, so these reactions have zero propensity. Reaction propensities, r, are re-calculated after each system update.

According to the model shown in Figure 1, the propensity of methylation, r_i^{me} for each histone i depends on the methylation status of each of the histones on neighboring nucleosomes and also the other histone on the same nucleosome. r_i^{me} also depends on the rates of recruited methylation k_{me}, noisy methylation, γ_{me}, and relative local PRC2 activity, β. For $1 \le i \le N$, the methylation reaction propensities are calculated as,

$$r_i^{me} = \beta\left(\delta_{S_i,\text{me0}}\left(\gamma_{\text{me0}-1} + k_{\text{me0}-1}E_i\right) + \delta_{S_i,\text{me1}}\left(\gamma_{\text{me1}-2} + k_{\text{me1}-2}E_i\right) + \delta_{S_i,\text{me2}}\left(\gamma_{\text{me2}-3} + k_{\text{me}}E_i\right)\right), \quad \text{(Equation S1)}$$

where $\delta_{x,y} = \begin{cases} 1, & x = y \\ 0, & x \neq y \end{cases}$, is the Kronecker delta and

$$E_i = \sum_{j \in M_i} \left(\rho_{\text{me2}}\delta_{S_j,\text{me2}} + \delta_{S_j,\text{me3}}\right), \quad \text{(Equation S2)}$$

is summed over 'neighboring' histones, where

$$M_i = \begin{cases} \{i-3, i-2, i-1, i+1, i+2\}, & i \text{ even}, \\ \{i-2, i-1, i+1, i+2, i+3\}, & i \text{ odd}. \end{cases} \quad \text{(Equation S3)}$$

This reflects the fact that each nucleosome consists of one even-numbered and one odd-numbered histone. Histones outside the simulated region are not considered. Consequently, histones on boundary nucleosomes have only one-sided recruitment of methylation. This introduces a slight bias toward the active state, as the boundary histones only have one-sided recruitment. However, since the region of chromatin domain simulated is relatively large (60 histones) relative to the boundaries (4 histones), we expect that this effect will be small.

Each histone i undergoes noisy removal of methyl groups (one methyl group at a time) with propensity,

$$r_i^{\mathrm{dem}} = \gamma_{\mathrm{dem}}\left(\delta_{S_i,\mathrm{me1}} + \delta_{S_i,\mathrm{me2}} + \delta_{S_i,\mathrm{me3}}\right). \quad \text{(Equation S4)}$$

Demethylation is also coupled directly to transcription, which itself has propensity given by Equation 3. Each transcription event can result in removal of methyl groups (one methyl group at a time) at each histone (with probability p_{dem} per histone) and also replacement of each nucleosome (me$x$/me$x \rightarrow$ me0/me0, with probability p_{ex} per histone). Since p_{ex} is a probability per histone and histone exchange actually results in replacement of a pair of H3 histones, the average rate of loss of histones through exchange is $\approx 2fp_{\mathrm{ex}}$.

To replicate DNA, the Gillespie algorithm simulation was interrupted if the projected time for the next reaction exceeded the time at which DNA would have been replicated. In this case, system time was updated to the forecast time of DNA replication. After replication of DNA, reaction propensities were then re-calculated and the Gillespie algorithm was repeated for another cell cycle. A similar approach was previously used to incorporate reactions with delays in Gillespie algorithm simulations (Bratsun et al., 2005).

Quantities Calculated from Simulations

Time-Averaging. For an individual simulation time-course comprising K reactions, the Gillespie algorithm determines the state of the system at K simulation time-points t_i (the trajectory). The time-step $\Delta t = t_{i+1} - t_i$ is not constant. Time-averaging for a quantity x_i (e.g. P_{OFF} or P_{ON}) between t_0 and t_K was performed using the formula,

$$\sum_{i=0}^{K-1} x_i \frac{t_{i+1} - t_i}{t_K - t_0}. \quad \text{(Equation S5)}$$

Bistability Measures. The quantity introduced in (Sneppen and Dodd, 2012) to determine the time-averaged probability of the system being in one of the epigenetic 'states' is equivalent to P_{OFF}, the probability that the number of repressive me2/me3 marks exceeds the number of neutral me0/me1 marks by at least half the total number of histones,

$$P_{\mathrm{OFF}} = \Pr\left(n_{\mathrm{me3}} + n_{\mathrm{me2}} - n_{\mathrm{me1}} - n_{\mathrm{me0}} > \frac{N}{2}\right). \quad \text{(Equation S6)}$$

With $N = n_{\mathrm{me3}}+n_{\mathrm{me2}}+n_{\mathrm{me1}}+n_{\mathrm{me0}}$, this reduces to,

$$P_{\mathrm{OFF}} = \Pr\left(n_{\mathrm{me3}} + n_{\mathrm{me2}} > \frac{3N}{4}\right). \quad \text{(Equation S7)}$$

Similarly,

$$P_{\mathrm{ON}} = \Pr\left(n_{\mathrm{me3}} + n_{\mathrm{me2}} < \frac{N}{4}\right), \quad \text{(Equation S8)}$$

and the bistability measure (Sneppen and Dodd, 2012) is given by,

$$B = 4P_{\mathrm{OFF}}P_{\mathrm{ON}}. \quad \text{(Equation S9)}$$

Since the histone type that is randomly inserted during DNA replication is identified with the high transcription state, it was necessary to allow the system to recover from this perturbation before assessing the stability of the state after DNA replication. For this reason, results were calculated only for the last hour of each cell cycle. This allowed systems with slow recovery times after DNA replication to attain high values of B, consistent with their long-term stability.

After introduction of the threshold, P_T (Equation 3), these definitions of P_{ON} and P_{OFF} no longer accurately reflect the chromatin state in terms of its control on expression. In this case, the gene is defined as being in the OFF-state if the chromatin-based regulation of transcription is in its lower quartile. For $f_{\max} \neq f_{\min}$,

$$P_{\mathrm{OFF}} = \Pr\left(f_{\max} - \frac{n_{\mathrm{me2}} + n_{\mathrm{me3}}}{NP_T}(f_{\max} - f_{\min}) < f_{\min} + \frac{f_{\max} - f_{\min}}{4}\right), \quad \text{(Equation S10)}$$

which can be simplified to,

$$P_{\mathrm{OFF}} = \Pr\left(n_{\mathrm{me3}} + n_{\mathrm{me2}} > \frac{3NP_T}{4}\right), \quad \text{(Equation S11)}$$

and likewise for P_{ON},

$$P_{\mathrm{ON}} = \Pr\left(n_{\mathrm{me3}} + n_{\mathrm{me2}} < \frac{NP_T}{4}\right). \quad \text{(Equation S12)}$$

With $P_T = 1$, Equations S11 and S12 reduce to Equations S7 and S8, respectively. These latter definitions are therefore consistent with earlier usage of the bistability measure B (Sneppen and Dodd, 2012). For all figures (except for the two-state model – Figure S1) Equations S11 and S12 were used to calculate the bistability measure B, according to Equation S9.

First Passage Times. Mean first passage times, $t_{FP(me0)}$ and $t_{FP(me3)}$, are defined as the average time taken for the system to change to the opposite chromatin state, when initialized in the uniform me0 or me3 state, respectively. For example, for an initially active state,

$$t_{FP(me0)} = \min\left(t \;\middle|\; n_{me3} + n_{me2} > \frac{3NP_T}{4}\right). \tag{Equation S13}$$

In the simulations, mean first passage times were bounded above by the total simulation time. This allowed the introduction of a quantity to measure the mutual stability of the two states, the 'combined first passage',

$$FP = \frac{t_{FP(me0)} t_{FP(me3)}}{T^2}, \tag{Equation S14}$$

where T is the total simulation time. Since $t_{FP(me0)}, t_{FP(me3)} \leq T$, then $0 < FP \leq 1$.

Two-State Model

To investigate if a simple two-state model (H3K27me0, H3K27me3) including transcription was capable of generating bistability, we constructed the model shown in Figure S1. In this model, PRC2 places me3 marks and transcription removes me3 marks. In addition, H3K27me3 represses transcription (Figure S1B, equation for f) and participates in positive feedback to recruit more PRC2 (Margueron et al., 2009). Previous studies have shown that bistability is most robust when interactions are 'long-ranged' (Dodd and Sneppen, 2011; Dodd et al., 2007). That is, PRC2 recruited anywhere in the gene can act on any other histone. Since we are interested in the ability of this model to generate bistability, we included such long-range interactions in this model. This was achieved by making the overall methylation rate dependent on the proportion of H3K27me3 marks at the gene (Figure S1B, equation for P_{me3}). The model also includes explicit noisy methylation and implicit noisy demethylation through stochastic transcription in the repressed state.

Simulations were performed in a similar manner to that described for the main model. Explicitly, for a system of N histones, the following reaction propensities r were calculated at every step of the Gillespie algorithm simulation:

$$r_i^{me} = \delta_{S_i,me0}\left(\gamma_{me} + \frac{k_{me}}{N}\sum_{j=1}^{N}\delta_{S_j,me3}\right), \tag{Equation S15}$$

$$r^{transcription} = f_{max} - \frac{1}{N}(f_{max} - f_{min})\sum_{j=1}^{N}\delta_{S_j,me3}, \tag{Equation S16}$$

where $1 \leq i \leq N$ and $S_j \in \{me0, me3\}$. Methylation reactions selected for histone i resulted in me0 to me3 conversion, whereas transcription events resulted in demethylation of each histone with probability, p_{dem} per histone.

We simulated this model over a large region of parameter space at high resolution, either in the presence or absence of DNA replication. Bistability was calculated using Equation S9, with

$$P_{OFF} = \Pr\left(n_{me3} > \frac{3N}{4}\right), \tag{Equation S17}$$

and

$$P_{ON} = \Pr\left(n_{me3} < \frac{N}{4}\right). \tag{Equation S18}$$

When included, DNA replication was modeled as a discrete event that occurred every 22 hr.

We were unable to find parameter sets that gave stability for both the active and repressed expression states (Figure S1D). Figures S1E and S1F show example trajectories of biased and balanced models without DNA replication. Note that even when methylation and demethylation processes are relatively balanced, neither state is stable over long periods of time (Figures S1E and S1F central panels).

Our results are in agreement with previous work showing that bistability is not obtained without nonlinearity in the histone modification conversion reactions (Dodd et al., 2007). Rather than adding such nonlinearity arbitrarily to generate the main model considered in this work, we find that nonlinearity arises parsimoniously from the non-processivity of H3K27-methylation by PRC2.

Processivity in Methylation or Demethylation

SET-domain histone methyltransferases, such as the catalytic subunit of PRC2, can be either processive or non-processive (Chin et al., 2006; Patnaik et al., 2004). However, as discussed in the main text there is in vitro and in vivo evidence that PRC2 acts non-processively when methylating H3K27. Moreover, the two-state model considered above, which did not generate bistability, corresponds approximately to a model with processive methylation and demethylation. We argued that the failure of the two-state

model was due to a lack of nonlinearity in the reactions converting between H3K27me0 and H3K27me3. It is therefore interesting to consider the ability of the full model to maintain both the active and repressed expression states when either methylation or demethylation (but not both) occur processively (Figure S2).

Processive Methylation

To investigate if bistability in our full model is dependent on non-processivity of the methyltransferase, we modified the model structure so that PRC2 catalyses the conversions me0 → me3, me1 → me3 and me2 → me3 instead of adding methyl groups one at a time (Figure S2B). All reaction propensity calculations remain unchanged. The model retains the relative catalytic activity of PRC2 on H3K27me0, me1 and me2 substrates of 9:6:1, respectively, because these quantities were calculated from experiments without reference to the reaction product produced (McCabe et al., 2012). Both noisy and recruited methylations are considered as processive.

In agreement with the results of our two-state model, we observed very limited bistability (Figure S2B), suggesting that non-processivity in methylation is an important feature for our model to provide cis epigenetic memory.

Processive Demethylation

In the model, processive demethylation plays a similar role to histone exchange – with the exception that processive demethylation results in conversion of one histone (me*x* → me0) while histone exchange results in removal of both histones on a nucleosome (me*x*/me*x* → me0/me0). Since the full model can generate bistability at reasonably high levels of histone exchange (Figure S5), we expected that including processive demethylation would not have a dramatic effect on bistability. We modified the model structure so that K27-demethylases (including noisy demethylation) performed the conversions me3 → me0, me2 → me0 and me1 → me0, rather than removing one methyl group at a time (Figure S2C). Again, all reaction propensity calculations remain unchanged. As expected, we found that the model was still able to generate bistability – albeit over a smaller region in parameter space (Figure S2C).

Transcriptional Bursting

In the main model developed in this work, transcription events occur stochastically with constant probability per unit time f at all times – where f depends on the current chromatin state and trans-activation level. That is, transcription is modeled as a Poisson process. However, it is known from studies in both prokaryotes and eukaryotes, that transcription often occurs in episodic 'bursts', interspersed with intervals of transcriptional inactivity (reviewed in (Raj and van Oudenaarden, 2008)). Models that explain this 'transcriptional bursting' typically consist of two or more promoter states, each with different characteristic transcriptional activities (Paulsson, 2005; Peccoud and Ycart, 1995; Raj et al., 2006). To verify that the conclusions presented in this work are valid even when transcription occurs in bursts, we now consider incorporating a more complex 'promoter-switching' description of transcription into our integrated chromatin/transcription model.

The model is shown in Figure S3, with additional parameters defined in Figures 1D and S6M. Following (Peccoud and Ycart, 1995), we assume that the promoter can exist in either an 'open' or 'closed' state. Transitions between these states occur with probabilities per unit time, k_{on} and k_{off}. When in the open-promoter state, transcription occurs with constant rate f_0, independent of the chromatin state and *trans*-activation level. For a given gene that displays transcriptional bursting, experiments suggest that transcriptional output can be regulated either by modulating burst size (transcripts per burst) or by modulating burst frequency, or a combination of both (Dar et al., 2012; Raj et al., 2006; Senecal et al., 2014). In our model, we consider the case in which regulation by chromatin and *trans*-factors alters the probability of transition from a closed to an open promoter state, k_{on}, while k_{off} is kept constant. That is, transcriptional regulation occurs through changes to burst frequency, with both the transcription rate of the open promoter state and the burst duration remaining, on average, fixed. However, since we consider large ranges of values for f_0, and k_{off}, a range of burst sizes and durations are also considered (in different simulations). Other than the changes to the regulation of transcription, the model remains unmodified from that considered in the main text.

The probability of being in the open promoter state when the gene is fully repressed is $P_{open(min)} = k_{on(min)}/(k_{on(min)} + k_{off})$, while when the gene is maximally active the corresponding probability is $P_{open(max)} = k_{on(max)}/(k_{on(max)} + k_{off})$. The maximal fold-change in transcription rate between the active and repressed chromatin states is therefore given by

$$F = \frac{f_0 P_{open(max)}}{f_0 P_{open(min)}} = \frac{k_{on(max)}\left(k_{on(min)} + k_{off}\right)}{k_{on(min)}\left(k_{on(max)} + k_{off}\right)}. \quad \text{(Equation S19)}$$

To ensure that this transcriptional fold-change is the same in the promoter-switching model as the main model ($F = f_{max}/f_{min} = 40$), we must therefore set

$$k_{on(min)} = \frac{k_{on(max)} k_{off}}{39 k_{on(max)} + 40 k_{off}} \quad \text{(Equation S20)}$$

Furthermore, to ensure that average transcription rates in the active and repressed states are the same as those of the main model, we also set

$$f_{min} = f_0 P_{open(min)}. \quad \text{(Equation S21)}$$

With f_{min} = 10^{-4} s^{-1} (Figure 1D), we therefore obtain,

$$f_0 = \frac{10^{-4}\left(k_{on(min)} + k_{off}\right)}{k_{on(min)}}. \quad \text{(Equation S22)}$$

With this formulation, the promoter is 40 times more likely to be open in the active than the repressed chromatin state; average burst duration is constant (determined by k_{off}); and the average rate of transcription from an open promoter is scaled to maintain the same mean transcription rate in the fully repressed state as in the main model. It is important to note, however, that for $k_{on(min)} \gg k_{off}$, $P_{open(min)} \approx 1$, and $f_0 \approx 10^{-4}$ s^{-1}. That is, the promoter is always 'open', even in the repressed chromatin state. In this regime, the model breaks down because transcription cannot be up-regulated by increasing k_{on}, and neither the chromatin state nor trans-factors can exert an activating effect on transcription. To ensure that the required transcriptional regulation can be achieved through modulation of k_{on} alone, we restrict our analysis to the region of parameter space where $k_{on(max)} \leq k_{off}$. This ensures that the average time between bursts is always longer than the average burst duration, which is consistent with experimental observations in mammalian cells (Dar et al., 2012; Molina et al., 2013; Skinner et al., 2016; Suter et al., 2011).

With this model formulation there are two free parameters that control the extent to which transcription occurs constitutively or in episodic bursts: $k_{on(max)}$ and k_{off}. Parameter values for chromatin dynamics obtained from fitting the main model remain unchanged in this model (P_T = 1/3, k_{me} = 8 × 10^{-6} histone^{-1}s^{-1}, p_{dem} = 4 × 10^{-3} histone^{-1}transcription^{-1}, p_{ex} = 10^{-3} histone^{-1}transcription^{-1}).

We simulated the promoter switching model over a range of values of $k_{on(max)}$ and k_{off} (Figure S4) and calculated P_{ON}, P_{OFF}, B, and FP from simulations. B was determined using Equation S9, with P_{ON}, P_{OFF} as in Equations S11 and S12. FP was calculated using Equation S14. Parameter ranges chosen include (but are not limited to) promoter on- and off-rates estimated from experiments (Dar et al., 2012; Molina et al., 2013; Skinner et al., 2016; Suter et al., 2011). Figures S4G–S4N show example simulations for selected parameters indicated in Figure S4A. Over this parameter range, average promoter-closed durations in the active expression state vary from much shorter than a cell cycle (e.g Figures S4G and S4H), to much longer than a cell cycle (e.g. Figures S4M and S4N). When $k_{on(max)}$ and k_{off} are both fast (short open and closed durations), burst size is $\leq$ 1, and transcription becomes approximately Poissonian. As expected, the model generates bistability in such cases (Figure S4E). However, as $k_{on(max)}$ is reduced, burst frequency is reduced (Figure S4B) and the transcription rate in the 'open' state increases (Figure S4A). For small enough values of $k_{on(max)}$, this causes instability of the active state because transcription does not occur frequently enough to prevent the accumulation of H3K27me2/me3 (as shown by the increase in P_{OFF} and reduction in B and FP as $k_{on(max)}$ is reduced in Figures S4D–S4F). However, this loss of bistability only occurs for very low values of $k_{on(max)} \approx 5 \times 10^{-5}$ s^{-1}, which corresponds to average promoter-closed durations of approximately 5 hr in the active state. Typical literature estimates for k_{on} in mammalian cells range from 10^{-4} to 10^{-3} s^{-1} (Dar et al., 2012; Molina et al., 2013; Skinner et al., 2016; Suter et al., 2011). Over this range, both the active and repressed states remain quite stable over a wide range of burst sizes and durations (Figures S4E and S4F), demonstrating that our model is capable of maintaining cis epigenetic memory even when transcription occurs in bursts.

Next, we determined the consequences of bursty transcription on robustness by examining its effect on the cis memory window. We first selected parameter values that gave bursty transcription within the range observed experimentally: $k_{on(max)}$ = 5 × 10^{-4} s^{-1} and k_{off} = 5 × 10^{-3} s^{-1} (corresponding to open-promoter durations of 3 minutes and closed durations of 30 minutes for the active state). Example simulations are shown in Figures S4O and S4P. Like the chromatin state, α influences k_{on} rather than f_0 in this model (see equation for k_{on} in Figure S3B). The main model considered in this work included a limit on the maximum probability per unit time of transcription initiation, $f \leq 1/60$ s^{-1}. In the promoter state-switching model, the rate of transcription initiation in the open promoter state, f_0 is determined by Equation S22. To maintain correspondence with the average transcription rates of the main model when $f_0 >$ 1/60 s^{-1}, we introduce a restriction on k_{on} by requiring that

$$f_0 P_{open} \leq 1/60\, s^{-1}. \quad \text{(Equation S23)}$$

Substituting $P_{open} = k_{on}/(k_{on} + k_{off})$ gives the condition,

$$k_{on} \leq \frac{k_{off}}{(60\, s)f_0 - 1}. \quad \text{(Equation S24)}$$

With these selected values of $k_{on(max)}$ and k_{off}, and the limitation on k_{on} imposed by Equation S24, we then performed simulations of the promoter-switching model at different fixed values of the trans-activation strength, α (similar to Figures 2C and 2D). Similar to the main model, we observed a robust window of trans-activation strengths within which the initial chromatin state tends to be maintained and therefore contributes to transcriptional output. Outside this window the H3K27 methylation state is determined entirely by the trans-activation strength (Figure S4Q). The transcriptional output increases more slowly as a function of α for the promoter-switching model than the non-bursty transcription model in the main text. This is because in the promoter-switching model, transcription is no longer a linear function of α, but rather it is a linear function of $P_{open} = k_{on}(\alpha)/(k_{on}(\alpha)+k_{off})$. We also calculated the mean first passage times for the active and repressed initial states as a function of α (Figure S4R). For both states, lifetimes are very slightly reduced for bursty versus non-bursty transcription, however average lifetimes greater than 200 cell cycles were still achieved when α = 1, again underlining the robustness of these states.

Overall, we have shown that our integrated model of transcription and chromatin is able to provide robust cis epigenetic memory over a wide range of transcriptional burst sizes and durations.

Additional Details of the Main Model

In the main text, we presented an overview and brief justification for features included in the model. For the sake of brevity, some details and additional considerations were omitted from the main text. We now discuss these points in more detail.

Noisy Demethylation

Transcription-coupled demethylation occurs on average with rate fp_{dem}. In the model, noisy demethylation occurs through both transcription-dependent and transcription-independent mechanisms. For simplicity, the rate of transcription-independent noisy demethylation, γ_{dem} is set equal to the rate of transcription-dependent noisy demethylation $f_{\min}p_{\mathrm{dem}}$. This ensures that in the maximally repressed state, demethylation occurs through both transcription-dependent and transcription-independent mechanisms with equal probability. With $f_{\max} = 40f_{\min}$, transcription-coupled demethylation in the repressed state ($f_{\min}p_{\mathrm{dem}}$) is equal to 2.5% of the rate of transcription-coupled demethylation in the active state ($f_{\max}p_{\mathrm{dem}}$). Together with transcription-independent noisy demethylation, $\gamma_{dem} = f_{\min}p_{\mathrm{dem}}$, the total rate of (noisy) demethylation in the repressed state is 5% of the maximum rate of transcription-coupled demethylation in the active state. This 'signal-to-noise' level in demethylation is therefore equivalent to that of noisy methylation (5%), which is captured by the parameters $\gamma_{me0-1} = k_{me0-1}/20$, $\gamma_{me1-2} = k_{me1-2}/20$, $\gamma_{me2-3} = k_{me}/20$, as described in the main text.

Mitosis

Throughout this work, the effect of chromosome condensation during mitosis on chromatin states has been ignored. During mitosis, histones are retained at similar locations and their H3K27-methylation status is maintained (Alabert et al., 2015; Annunziato, 2005; Gaydos et al., 2012). It is also known experimentally that transcription is actively repressed (Spencer et al., 2000) and that the majority of Polycomb group proteins dissociate from chromatin (Fonseca et al., 2012). This suggests that both transcription and H3K27-methylation occur with lower probability on condensed chromatin during mitosis. Based on these data, it is assumed that chromatin states are not substantially biased toward activation or repression during mitosis. With this assumption, mitosis effectively represents a 'pause' in the state of the system and is therefore not included in the model.

Active Chromatin Marks

In our main model, we showed that transcription-coupled histone demethylation and histone exchange constitute sufficient antagonism of PRC2 silencing to ensure robust stability of the active state. However, considerable molecular and genetic evidence indicates that Polycomb repression is also antagonized by the Trithorax group of proteins (Klymenko and Müller, 2004; Petruk et al., 2001). This is thought to be mediated in part by H3K4 and H3K36 methylation, which are commonly associated with highly transcribed genes and are refractory to PRC2-mediated H3K27 methylation (Tie et al., 2014; Yuan et al., 2011). However, it is currently unclear if any of these 'active marks' are capable of positive feedback independent from transcription. Without such *direct* positive feedback, these 'active marks' are not sufficient to instruct their own maintenance and were therefore omitted from our model. One possibility to explain the requirement for Trithorax group proteins in antagonism of PRC2 (Klymenko and Müller, 2004; Tie et al., 2014) is that these active histone marks are laid down by transcription-coupled processes in order to antagonize PRC2-silencing. In addition, these marks could increase the probability of transcription initiation by promoting histone acetylation, including that of H3K27 (Tie et al., 2014). Together, these two effects would generate an *indirect* positive feedback for active marks mediated by transcription. This could easily be included as an extension to our model and would constitute another mechanism by which transcription antagonizes PRC2. By stabilizing the active state, this would increase the width of the cis-memory window. However, there may still be cases where transcription is less involved in the antagonism of Polycomb silencing, a potential example being the *bxd* Polycomb Response Element (PRE) in Drosophila (Erokhin et al., 2015).

Histone Exchange

Many experimental studies have attempted to quantify rates of histone exchange. Metabolic labelling experiments in *Saccharomyces cerevisiae* indicated that H2B is exchanged more often than H3, and that H3 exchange is correlated with gene expression level (Dion et al., 2007; Jamai et al., 2007). These studies found that up to 50% of H3 over the coding region could be replaced within one hour, but failed to detect H3 exchange at inactive genes. Similarly, pulse-chase experiments in Drosophila cell culture estimated mean histone residence times of a few hours at actively transcribed genes (Deal et al., 2010). These measurements were, however, limited to a short labelling duration, preventing accurate determination of slow rates of exchange.

Histone exchange rates have also been measured by microscopy, using Fluorescence Recovery After Photobleaching (FRAP) of fluorescently-labelled histones (Kimura and Cook, 2001). In HeLa cells, this suggested a wide range of histone exchange rates across the genome, with a substantial portion of H3 and H4 histones remaining in place over the entire experiment, lasting 8.5 hr.

Relative rates of histone exchange across the genome have also been inferred from the patterns of accumulation of H3 variants H3.1 and H3.3 (Jin et al., 2009). Histone H3.3 is incorporated in chromatin independently of DNA replication, while H3.1 incorporation is coupled to replication (Tagami et al., 2004). In human and mouse cells, H3.3 levels are positively correlated with transcriptional activity (Ray-Gallet et al., 2011), and both H3.3 and histone exchange are reduced at repressed Polycomb targets (Deaton et al., 2016; Kraushaar et al., 2013). These data are consistent with histone exchange being slow at repressed PRC2 target genes, but occurring on time-scales similar to (or faster than) the cell cycle when these same genes are highly transcribed.

The mechanistic basis of the transcription-dependence of histone exchange is unknown (reviewed in (Venkatesh and Workman, 2015)). This effect may be due to a more compact chromatin structure and lower levels of histone acetylation at repressed genes, which tends to promote retention of histones (reviewed in (Zentner and Henikoff, 2013)). Alternatively, transcription may be physically coupled to the exchange machinery (Ray-Gallet et al., 2011), or histones may sometimes be lost as Pol II traverses the nucleosome (Kulaeva et al., 2013). All of these possibilities result in removal of modified histones with low probability at each transcription event. In

the model, we therefore chose to couple histone exchange to transcription. That is, each passage of Pol II in the model has the capacity to remove an H3/H4 tetramer. Actual histone exchange rates in the model depend on both the probability of histone exchange per transcription event, and the transcription initiation rate, f. Because histone exchange is directly coupled to transcription, the maximum fold-change in the transcription initiation rate, $F = f_{max}/f_{min}$ provides an upper bound on the fold-change in histone residence times between the active and repressed states. To break this linear coupling would require a more complicated function relating transcription and histone exchange. Without additional information about how histone exchange changes as a function of transcriptional activation, there is little rationale for such a change. Therefore, we chose the simplest function that yields the conserved correlation between histone lifetime and transcription level.

Transcription-Dependent H3.3 Accumulation Constrains the Histone Exchange Probability

Having adopted this functional relationship between transcription and histone exchange in our model, it is necessary to set a parameter value for the histone exchange probability, p_{ex}. The value adopted in Box 1, and used throughout the remainder of the manuscript was $p_{ex} = 10^{-3}$ histone^{-1} transcription^{-1}. We now show that with this parameter value, our model can reproduce the experimental observations of transcription-dependent H3.3 accumulation, and low histone exchange in the repressed state (Deaton et al., 2016; Kraushaar et al., 2013; Ray-Gallet et al., 2011).

To quantify H3.3 accumulation in our model, we performed simulations in which histones incorporated during transcription-dependent histone exchange were labelled as ‘H3.3’, while those incorporated at DNA replication were labelled as ‘H3.1’ (Figures S6A and S6B). We then calculated the difference in H3.3 levels between simulations initialized in the active state and those initialized in the repressed state ($H = |\langle H3.3\rangle_{ON} - \langle H3.3\rangle_{OFF}|$, where $\langle\rangle$ indicates a time-average). In the bistable regime, high H values indicate strong transcription-dependence of H3.3 abundance, as experimentally observed. Although histone exchange is directly coupled to transcription in our model, transcription-dependent H3.3 accumulation is not automatically obtained for all bistable parameter sets (Figures S6C and S6D). For example, if p_{ex} is too low, H3.3 does not accumulate even in the active state (Figure S6F), and if p_{ex} is too high, H3.3 accumulates even in the repressed state (Figure S6K). However, with $p_{ex} = 10^{-3}$ histone^{-1} transcription^{-1}, and the fitted parameter values in Figure S6M, our model reproduces two semi-quantitative experimental results: low histone exchange in the repressed state and transcription-dependent H3.3 accumulation.

Fitting Triple-SILAC Mass Spectrometry Data

Published SILAC data (Alabert et al., 2015) were generated in the laboratories of Anja Groth (Biotech Research and Innovation Center, Copenhagen) and Axel Imhof (Ludwig-Maximilians Universität, Munich), and were obtained as processed data from Carsten Marr (Institute of Computational Biology, Helmholtz Zentrum, Munich), with permission.

As described in (Alabert et al., 2015), data were normalised to yield H3K27me3 levels on ‘old’ and ‘new’ histones as a proportion of the total old and new labeled peptides measured at each time point. Simulation results for H3K27me3 levels on old and new histones were initially also expressed as a proportion of the levels of old and new histones, respectively. However, because mass spectrometry data represent a genome-wide average, and simulations represent a single PRC2-target gene, simulation data must be scaled in order to make a quantitative comparison with experiments. To do so, simulation data were further normalised so that the average simulated cell-cycle-end value of H3K27me3 on total histones, $P_{me3\ end}$ was equal to the proportion of H3K27me3 on old histones at $t = 0$ (0.301), obtained experimentally. That is, each simulation time point was multiplied by the factor $0.301/P_{me3\ end}$. This is valid because all histones are labeled as old at $t = 0$, so the value 0.301 also represents the relative amount of H3K27me3 on total histones at the end of each cell cycle.

After this normalisation, the $t = 0$, 10, 24, 48 hr experimental time points for old and new histones were compared with equivalent model time-points using the sum of squared errors. Three biological replicates were available for each time point (Alabert et al., 2015).

The normalisation procedure requires that the model is epigenetically stable over many cell cycles in the repressed state in order that the extracted $P_{me3\ end}$ correctly normalises the simulated data at the start of the cell cycle in which ‘new’ histones are added. In Figures S7A and S7B, it can be seen that the normalisation fails for some of the unstable models for low values of k_{me}. This is because the repressed (high-me3) state is generally not maintained through the equilibration cycles before new histones are added.

We did not attempt to fit our model to time-dependent data for H3K27me1 because not all H3K27me1 in the genome is dependent on PRC2 (Ferrari et al., 2014). Nor did we fit H3K27me2, because this modification forms large intergenic and intragenic domains beyond the scope of our current model (Ferrari et al., 2014). Nevertheless, since our model incorporates non-processive H3K27 methylation with rates $k_{me0-1} > k_{me1-2} > k_{me2-3} = k_{me}$, it is qualitatively consistent with slower accumulation of H3K27me3 than H3K27me1 and H3K27me2, as observed experimentally (Alabert et al., 2015).

It is also important to remember that the SILAC data represent genome-wide averages. It is therefore not guaranteed that the timescale extracted through the analysis reflects that of a gene whose repression actually depends on H3K27me2/me3. For this reason, faster H3K27 methylation dynamics (similar to Figures B1B and B1D) cannot be excluded in all cases.

Stochastic Model of a Noisy Transcriptional Regulator

The following model was used in (Ozbudak et al., 2002) to investigate how rates of transcription and translation affect variability in protein abundance over time. In the present work it is used as an arbitrary 'noisy' input function representing the expression of a *trans*-regulator:

$$DNA \overset{s_R}{\rightarrow} mRNA \overset{d_R}{\rightarrow} \phi \quad \text{(Equation S25)}$$

$$mRNA \overset{s_P}{\rightarrow} Protein \overset{d_P}{\rightarrow} \phi. \quad \text{(Equation S26)}$$

In steady state, $\langle \text{mRNA} \rangle = s_R/d_R$ and $\langle \text{Protein} \rangle = s_R b/d_P$, where $\langle \rangle$ indicates an average over time and $b = s_P/d_R$ is the average number of proteins synthesised per mRNA transcript (Ozbudak et al., 2002). The 'noise' in protein abundance is controlled by the value of b, with larger b giving a more variable output.

To simulate a transcriptional regulatory protein with variable concentration $r(t)$, the following parameter values were used, d_R = 1/2 hr^{-1}, d_P = 1/12 hr^{-1}, $S_R = d_p \langle r(t) \rangle / b$ $hour^{-1}$, $s_P = d_R b$ $hour^{-1}$. Specifying the mean number of regulatory proteins as $\langle r(t) \rangle = 1000$, the noise can then be varied using the single parameter (B) Higher values of b indicate greater noise. The variable gene activation function $\alpha(t)$ is then given by $\alpha(t) = r(t)/\langle r(t) \rangle$.

The number of protein and RNA molecules were explicitly simulated using the Gillespie algorithm according to the model specified in Equations S25 and S26. These simulations to generate $\alpha(t)$ were performed concurrently with simulations of the chromatin state.

To generate Figure 3A, stochastic simulations of $\alpha(t)$ used $b \in$ {1, 2, 9, 23, 43, 71, 106, 149, 200, 259, 327, 404, 489, 583, 687, 799, 922, 1053, 1195}. This generated stochastic inputs $\alpha(t)$ with noise ranging from CV $\approx$ 0–1. Figures 3B–3E and S8B–S8E used b = 1 for 'low noise' (CV $\approx$ 0) and b = 1,000 for 'high noise' (CV $\approx$ 1).

Cell Systems, Volume *4*

Supplemental Information

Slow Chromatin Dynamics Allow Polycomb Target Genes to Filter Fluctuations in Transcription Factor Activity

Scott Berry, Caroline Dean, and Martin Howard

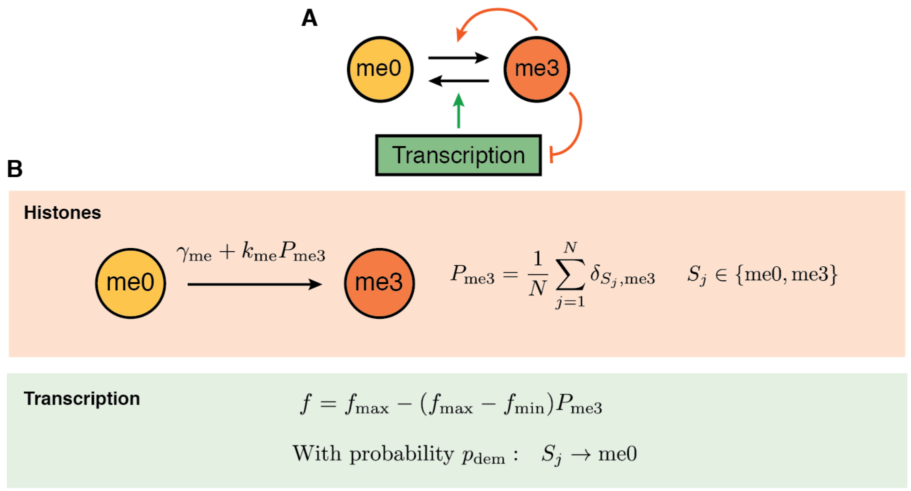

C

Parameter	Description	Value
k_{me}	PRC2-mediated methylation rate (histone^{-1}s^{-1})	Free
$f_{\max}$	Maximum transcription initiation rate (s^{-1})	Free
p_{dem}	Demethylation probability (histone^{-1}transcription^{-1})	Free
γ_{me}	Noisy methylation rate (histone^{-1}s^{-1})	$k_{\text{me}}/20$
$f_{\min}$	Minimum transcription initiation rate (s^{-1})	10^{-4}
N	Number of histones	60

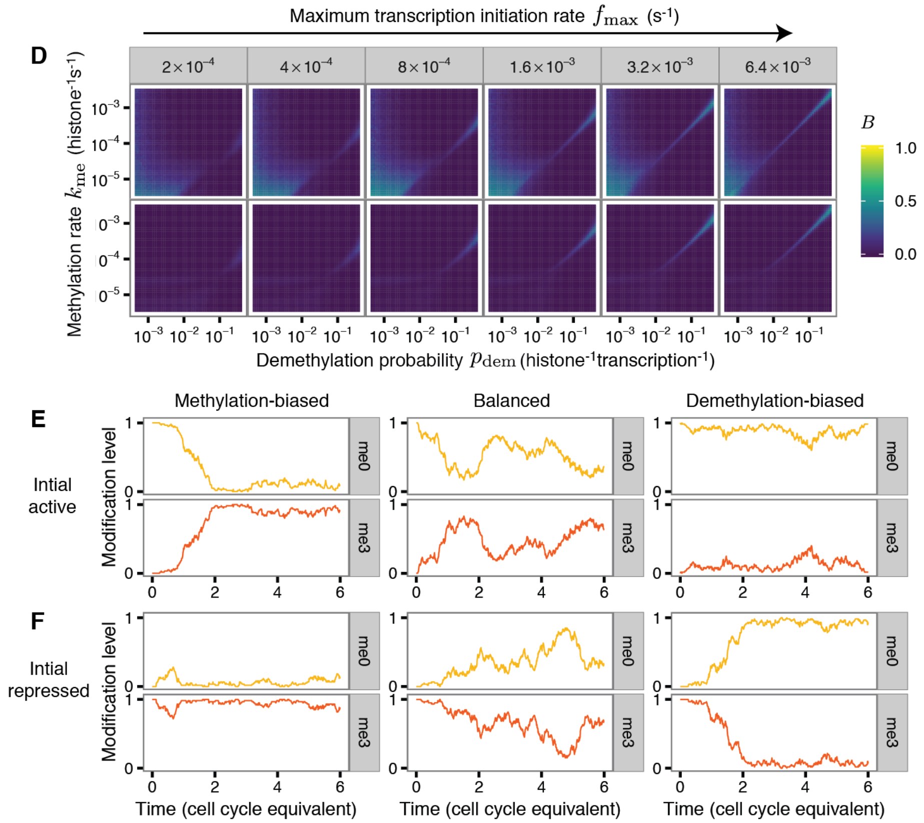

Figure S1, related to Figure 1: Two-state model. (A) Diagrammatic representation of feedbacks in the two-state mathematical model. States me0 and me3 refer to the methylation state of H3K27. Black arrows represent state transitions, while coloured arrows represent feedback interactions. (B) Mathematical

description of the model. (C) Two-state model parameters. (D) Heat-map indicating the bistability measure, B, for simulations performed with k_{me} (histone^{-1}s^{-1}), p_{dem} (histone^{-1} transcription^{-1}), and $f_{\max}$ (s^{-1}) values indicated on axes and panel labels. For each parameter set, 100 simulations were initialized in each of the uniform me0 or me3 states and simulated for 50 cell cycles. Bistability measure B calculated as described in STAR Methods. DNA replication is not included in the top panels but is included in the bottom panels, with a timescale of 22 hours (Posakony et al., 1977). (E) Example simulations of a single genomic locus initialized in the active (me0) state, for methylation-biased ($p_{\mathrm{dem}} = 6.3\times10^{-3}$ histone^{-1} transcription^{-1}), balanced ($p_{\mathrm{dem}} = 10^{-2}$ histone^{-1} transcription^{-1}), or demethylation-biased ($p_{\mathrm{dem}} = 1.5\times10^{-2}$ histone^{-1} transcription^{-1}) parameter sets. (F) As in E, except with initially repressed (me3) state. In all examples, $k_{me} = 1.25\times10^{-4}$ histone^{-1}s^{-1} and $f_{\max} = 0.0128$ s^{-1}.

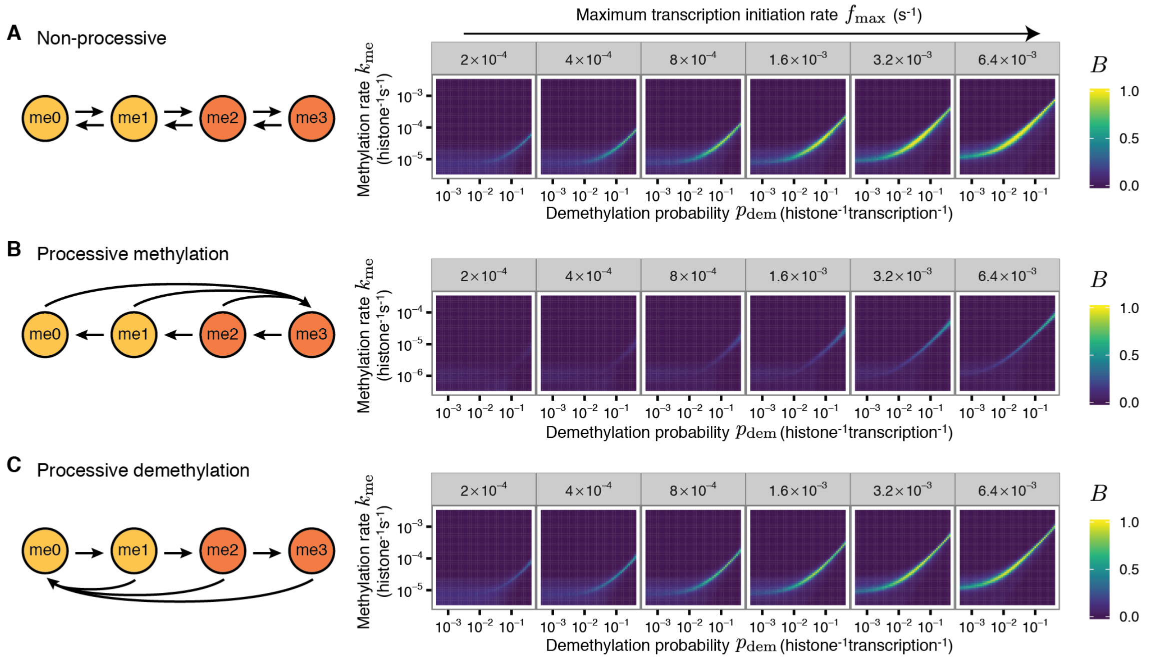

Figure S2, related to Figure 1: Effect of processivity in enzyme activity on bistability. Left panels show model schematics with black lines indicating possible state transitions. Right panels show heat maps of the bistability measure, B, calculated from simulations performed over a range of values for the parameters k_{me} (histone^{-1}s^{-1}), p_{dem} (histone^{-1} transcription^{-1}) and $f_{\max}$ (s^{-1}). Each panel shows B as a function of k_{me} and p_{dem}, for the value of $f_{\max}$ shown in the panel label. For each parameter set, 100 simulations were initialized in each of the uniform me0 or me3 states and simulated for 50 cell cycles. Results averaged over all simulations. All simulations have $p_{\mathrm{ex}} = 10^{-3}$ (histone^{-1} transcription^{-1}). Except for changes to processivity, model and other parameters as in Figure 1C and D. (A) Non-processive methylation and demethylation, reproduced from Figure 1G for comparison. (B) Model with only processive methylation, and non-processive demethylation. (C) Model with only processive demethylation, and non-processive methylation.

A

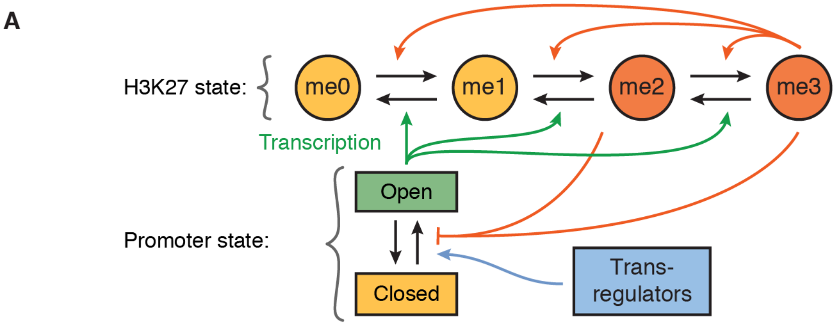

B

Histones

$$\text{me0} \underset{\gamma_{\text{dem}}}{\overset{\gamma_{\text{me0-1}} + k_{\text{me0-1}} E_i}{\rightleftharpoons}} \text{me1} \underset{\gamma_{\text{dem}}}{\overset{\gamma_{\text{me1-2}} + k_{\text{me1-2}} E_i}{\rightleftharpoons}} \text{me2} \underset{\gamma_{\text{dem}}}{\overset{\gamma_{\text{me2-3}} + k_{\text{me}} E_i}{\rightleftharpoons}} \text{me3}$$

$$E_i = \sum_{j \in \text{neighbors of } i} \left(\rho_{\text{me2}} \delta_{S_j,\text{me2}} + \delta_{S_j,\text{me3}} \right) \qquad S_j \in \{\text{me0}, \text{me1}, \text{me2}, \text{me3}\}$$

Promoter switching

$$\text{Closed} \underset{k_{\text{off}}}{\overset{k_{\text{on}}}{\rightleftharpoons}} \text{Open}$$

$$P_{\text{me2/me3}} = \frac{1}{N} \sum_{j=1}^{N} \left(\delta_{S_j,me2} + \delta_{S_j,me3} \right)$$

$$k_{\text{on}} = \begin{cases} \alpha \left(k_{\text{on(max)}} - \frac{P_{\text{me2/me3}}}{P_T} \left(k_{\text{on(max)}} - k_{\text{on(min)}} \right) \right) & , P_{\text{me2/me3}} < P_T \\ \alpha k_{\text{on(min)}} & , P_{\text{me2/me3}} \geq P_T \end{cases}$$

Transcription

$$f = \begin{cases} f_0 & \text{, if promoter is "Open"} \\ 0 & \text{, if promoter is "Closed"} \end{cases}$$

$$\text{With probability } p_{\text{dem}} \text{ per histone } j: \quad S_j \to \begin{cases} \text{me0} & \text{if } S_j = \text{me1} \\ \text{me1} & \text{if } S_j = \text{me2} \\ \text{me2} & \text{if } S_j = \text{me3} \end{cases}$$

$$\text{With probability } p_{\text{ex}} \text{ per histone } j: \quad \begin{cases} S_j S_{j+1} \to \text{me0}\,\text{me0}, & \text{if } j \in \{1, 3, 5, \ldots, N-1\} \\ S_{j-1} S_j \to \text{me0}\,\text{me0}, & \text{if } j \in \{2, 4, 6, \ldots, N\} \end{cases}$$

DNA replication

$$\text{With probability } \frac{1}{2}: \quad S_j S_{j+1} \to \text{me0}\,\text{me0}, \text{ for } j = 1, 3, 5, \ldots, N-1$$

C

Parameter	Description	Value
$k_{\text{on(max)}}$	Maximum transition rate between 'closed' and 'open' promoter state (s^{-1})	Free
k_{off}	Transition rate between 'open' and 'closed' promoter state (s^{-1})	Free
$k_{\text{on(min)}}$	Minimum transition rate between 'closed' and 'open' promoter state (s^{-1})	$k_{\text{on(max)}} k_{\text{off}} / \left(39 k_{\text{on(max)}} + 40 k_{\text{off}} \right)$
f_0	Transcription initiation rate ('open' promoter) (s^{-1})	$10^{-4} \left(k_{\text{off}} + k_{\text{on(min)}} \right) / k_{\text{on(min)}}$

Figure S3 related to Figure 1: Promoter-switching model. (A) Diagrammatic representation of feedbacks in mathematical model. States me0 to me3 refer to methylation state of H3K27. Neutral marks me0/me1 indicated in yellow, repressive marks me2/me3 in orange. Black arrows represent state transitions; coloured arrows represent feedback interactions. For clarity, histone exchange and H3K27me2-mediated recruitment of PRC2 are omitted. Promoter states represented as 'open' or 'closed'. Transcription is possible only in the open state. (B) Mathematical description of model. Sum over 'neighbours' in E_i

includes the other histone on same nucleosome, and four histones on neighbouring nucleosomes. $\delta_{i,j}$: Kronecker delta, equal to 1 if $i = j$ and 0 otherwise. $P_{\mathrm{me2/me3}}$ is the fraction of H3 histones carrying K27me2 or K27me3. (C) Extra parameters added to the minimal model to incorporate promoter switching. Other parameters defined in Figures 1D and S6M.

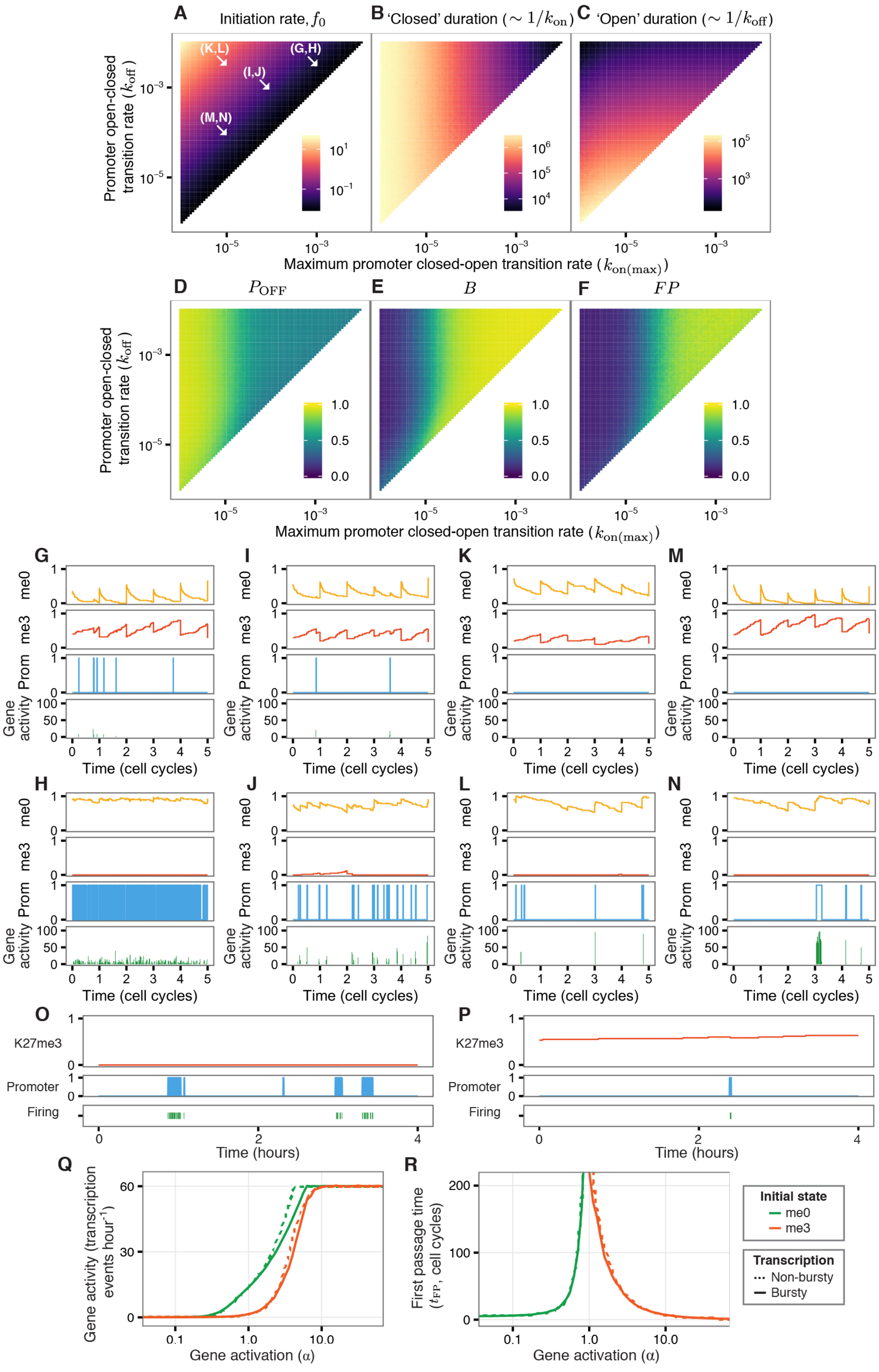

Figure S4 related to Figures 1 and 2: Promoter-switching model results. Heat maps showing (A) transcription initiation rate, f_0, (B) average duration of a 'closed' state, (C) average duration of an 'open'

state, (D) probability of being in the repressed transcriptional state, (E) bistability measure, B and (F) combined first passage time measure, FP. In A, f_0 specified as an input parameter while B-F show average results over 500 simulations from each of the uniform me0 and uniform me3 states. Each panel shows results plotted as a function of $k_{\text{on(max)}}$ and k_{off} (10^{-2} s^{-1} to 10^{-6} s^{-1}) with simulations restricted to $k_{\text{on(max)}} \leq k_{\text{off}}$. Model defined in Figure S3 with additional parameters in Figures 1D and S6M, ($\alpha = \beta = 1$). (G-N) Example simulations of promoter switching model with variable transcriptional bursting kinetics. Upper row simulations initialised in the repressed (uniform me3) chromatin state, while lower row simulations initialised in the active (uniform me0) chromatin state. Simulations equilibrated for 5 cell cycles before plotting a further 5 cell cycles. Prom (promoter) state represented as 1 for 'open' and 0 for 'closed'. Gene activity represented as the number of transcription events per 30-minute interval. As indicated in A, parameter values are (G, H) $k_{\text{on(max)}} = 10^{-3}$s^{-1} , $k_{\text{off}} = 3 \times 10^{-3}$ s^{-1}. (I, J) $k_{\text{on(max)}} = 10^{-4}$s^{-1} , $k_{\text{off}} = 10^{-3}$ s^{-1}. (K, L) $k_{\text{on(max)}} = 10^{-5}$s^{-1} , $k_{\text{off}} = 3 \times 10^{-3}$ s^{-1}. (M, N) $k_{\text{on(max)}} = 10^{-5}$s^{-1} , $k_{\text{off}} = 10^{-4}$ s^{-1}. Other parameters in Figures 1D and S6M. (O) Example time-course simulation over a short time-scale showing H3K27me3 levels, promoter state and transcription initiation events for the promoter-switching model with $k_{\text{on(max)}} = 5 \times 10^{-4}$s^{-1} , $k_{\text{off}} = 5 \times 10^{-3}$ s^{-1}. Other parameters in Figures 1D and S6M. Simulation first equilibrated for 5.5 cell cycles from active (uniform me0) initial chromatin state ($\alpha = \beta = 1$). (P) As in O, except initialised in repressed (uniform me3) state. (Q) Gene activity in the promoter-switching model ('Bursty', solid lines) measured as average number of transcription events (gene^{-1}hour^{-1}) in 20th cell cycle after activation or repression, averaged over 2000 simulations for each value of α. Green lines indicate initially active gene; orange lines indicate initially repressed gene. $\alpha = 1$ during 5 cell-cycle equilibration starting from uniform me0 or me3 state, then α as indicated on *x*-axis for further 20 cell cycles. (R) Mean first passage time in the promoter-switching model ('Bursty', solid lines), t_{FP} (STAR methods) as function of α, averaged over 1000 simulations each of 1500 cell cycles, from initially active or repressed state. $k_{\text{on(max)}} = 5 \times 10^{-4}$s^{-1} , $k_{\text{off}} = 5 \times 10^{-3}$ s^{-1} and $\beta = 1$ throughout Q and R, with other parameters in Figures 1D and S6M. Results from the main model with non-bursty transcription are shown with dashed lines for comparison (replotted from Figure 2C,D –upper panels).

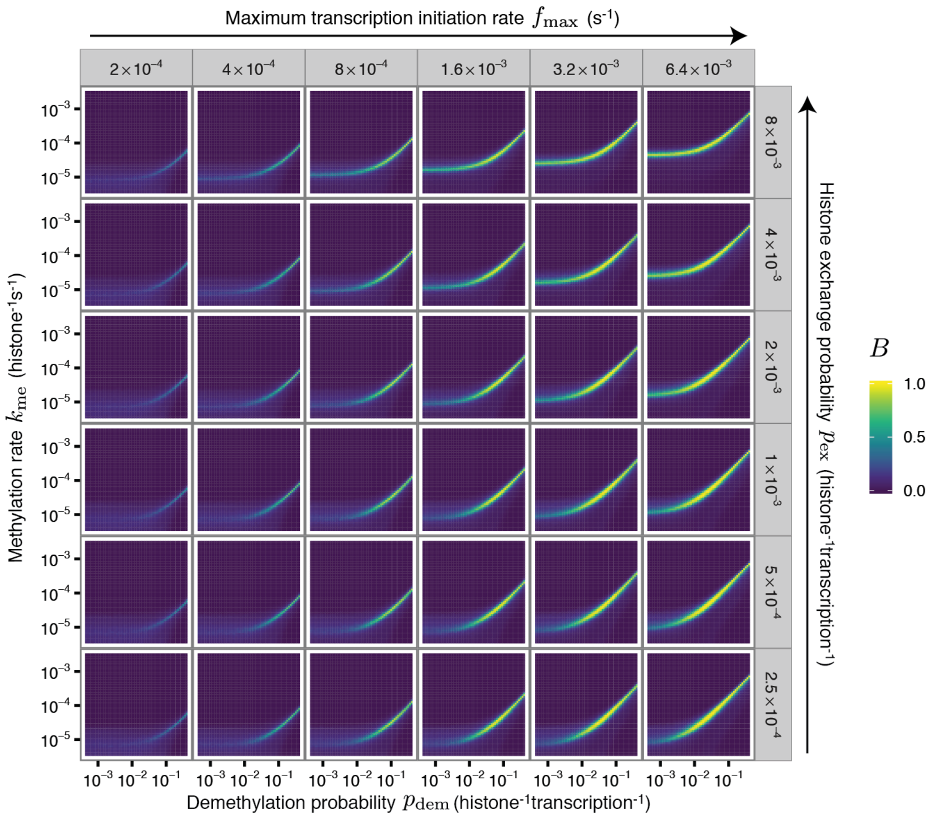

Figure S5, related to Figure 1: Model bistability for various histone exchange probabilities. Heat map showing bistability measure B, calculated from simulations as described in Figure 1 legend. Each panel shows B as function of k_{me} and p_{dem}, for f_{max} and p_{ex} values shown in panel labels (top and right, respectively).

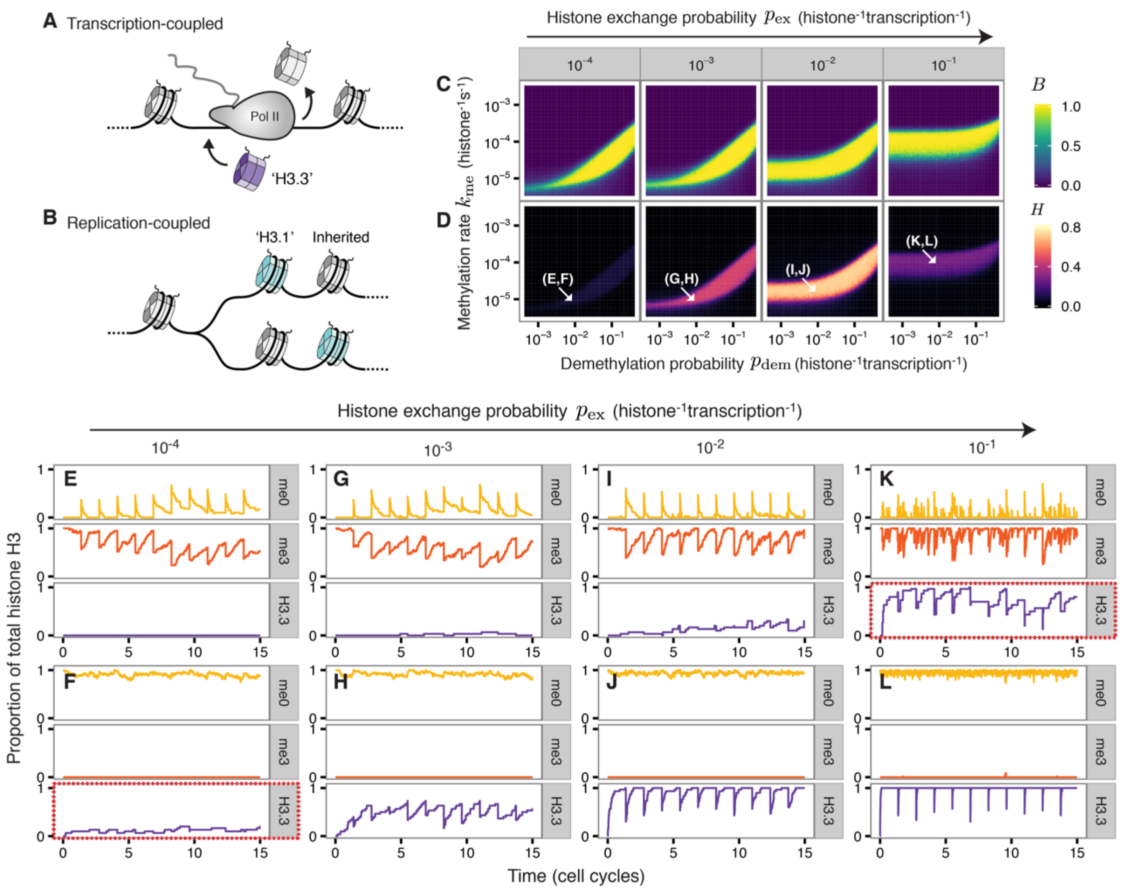

M

Parameter	Description	Value
k_{me}	PRC2-mediated methylation rate (me2 to me3) (histone^{-1}s^{-1})	8×10^{-6}
f_{max}	Maximum transcription initiation rate (s^{-1})	$4 \times 10^{-3}(40 f_{\mathrm{min}})$
p_{dem}	Demethylation probability (histone^{-1}transcription^{-1})	4×10^{-3}
p_{ex}	Histone exchange probability (histone^{-1}transcription^{-1})	10^{-3}
P_T	Threshold proportion of me2/me3 marks for maximal repression	$1/3$

Figure S6, related to Figure B1: Fitting the histone exchange rate to reproduce transcription-dependent H3.3 accumulation. (A) Schematic of transcription-coupled histone exchange, resulting in H3.3 incorporation (purple nucleosome). (B) Schematic of replication-coupled deposition of H3.1 (cyan nucleosomes). (C) Bistability measure, B, and (D) difference in average H3.3 levels between simulations initialized in the active state and those initialized in the repressed state ($H = |\langle \mathrm{H3.3} \rangle_{\mathrm{ON}} - \langle \mathrm{H3.3} \rangle_{\mathrm{OFF}}|$, where $\langle\ \rangle$ indicates an average over time). In C,D, 100 simulations were initialized in each of the uniform me0/H3.1 or me3/H3.1 states and simulated for 50 cell cycles for each parameter set. Each panel shows B or H as function of k_{me} and p_{dem}, for p_{ex} shown in panel label. Model as in Figure 1 (as modified by Equation 3). $P_T = 1/3$, $f_{\mathrm{max}} = 40 f_{\mathrm{min}}$, with other parameters in Figure 1D. (E-L) Example simulations for parameters indicated in D. Top row initialized in the uniform me3/H3.1 state. Bottom row initialized in the uniform me0/H3.1 state. (E,F) $k_{\mathrm{me}} = 10^{-5}$ histone^{-1}s^{-1}, $p_{\mathrm{ex}} = 10^{-4}$ histone^{-1} transcription^{-1}. (G,H) $k_{\mathrm{me}} = 10^{-5}$ histone^{-1}s^{-1}, $p_{\mathrm{ex}} = 10^{-3}$ histone^{-1} transcription^{-1}. (I,J) $k_{\mathrm{me}} = 2 \times 10^{-5}$ histone^{-1}s^{-1}, $p_{\mathrm{ex}} = 10^{-2}$ histone^{-1} transcription^{-1}. (K,L) $k_{\mathrm{me}} = 10^{-4}$ histone^{-1}s^{-1}, $p_{\mathrm{ex}} = 10^{-1}$ histone^{-1} transcription^{-1}. Red boxes in F

and K indicate lack of H3.3 in the active state, and H3.3 accumulation in the repressed state, respectively. For E-L, $p_{\mathrm{dem}} = 10^{-2}$ histone^{-1} transcription^{-1}. (M) Values of parameters after fitting the model to SILAC data and H3.3 accumulation, and optimizing for bistability.

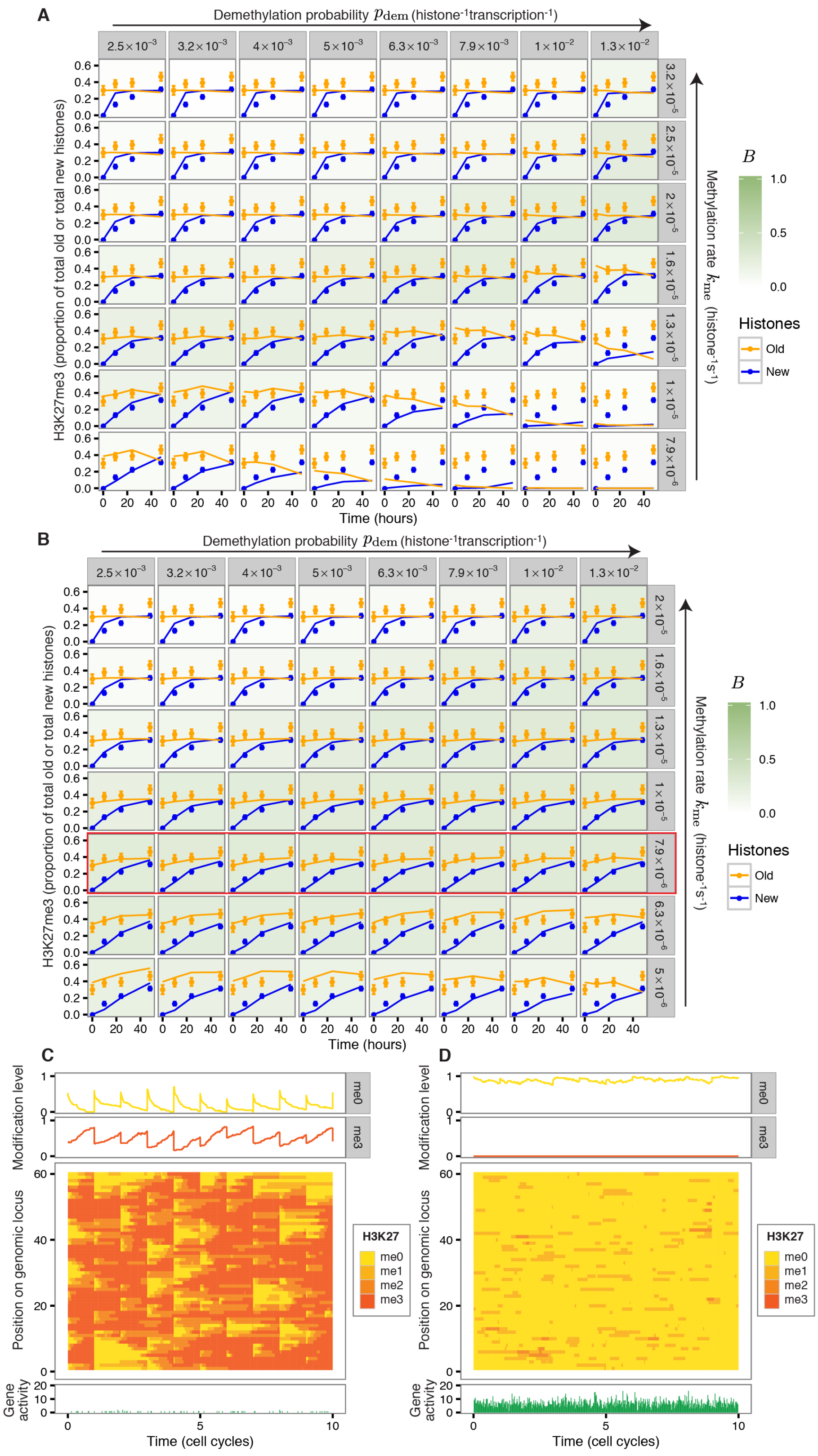

Figure S7, related to Figure B1: Fitting quantitative SILAC data. (A) Detailed fit to data over parameter space with $P_T = 1$. Each panel shows the experimentally determined H3K27me3 level on old

and new histones 0, 10, 24 and 48 hours after the first DNA replication in the SILAC experiment. Data are represented as the fraction of H3K27me3 on old and new histones, respectively. Solid lines indicate the model prediction, linearly interpolated between 0, 10, 24, and 48 hour time-points, which are each averages over 1000 SILAC simulations, normalised as described in Figure B1 legend (STAR methods). Each panel shows the results of simulations for a single pair of k_{me} and p_{dem} values. p_{dem} increases from left to right, while k_{me} increases from bottom to top. Model as in Figure 1 (as modified by Equation 3). $P_T = 1$, $f_{\mathrm{max}} = 40 f_{\mathrm{min}}$, and $p_{\mathrm{ex}} = 10^{-3}$ histone^{-1} transcription^{-1}, with other parameters in Figure 1D. The background shading of each panel (green) represents the bistability parameter, B, calculated using the same parameters. (B) Same as A, except with $P_T = 1/3$. Red box indicates methylation rate that gave the best fit from values shown (quantitative fit over parameter space shown in Figure B1F). (C,D) Spatially resolved simulations with fitted methylation rate. Example stochastic simulations of the fitted model ($P_T = 1/3$, $k_{\mathrm{me}} = 8 \times 10^{-6}$ histone^{-1}s^{-1}, $p_{\mathrm{dem}} = 4 \times 10^{-3}$ histone^{-1} transcription^{-1}, $f_{\mathrm{max}} = 40 f_{\mathrm{min}}$, $p_{\mathrm{ex}} = 10^{-3}$ histone^{-1} transcription^{-1}), from repressed and active initial states, respectively. Other parameters in Figure 1D. All panels show 10 cell cycles of simulation data, obtained after 5-cell cycles of equilibration. Upper panels show the levels of me0 and me3 over time, averaged over all histones in the locus. Middle panels show kymographs of H3K27 methylation status over time for each histone in the simulated region. Lower panels show gene activity measured as number of transcription events per 30 min interval.

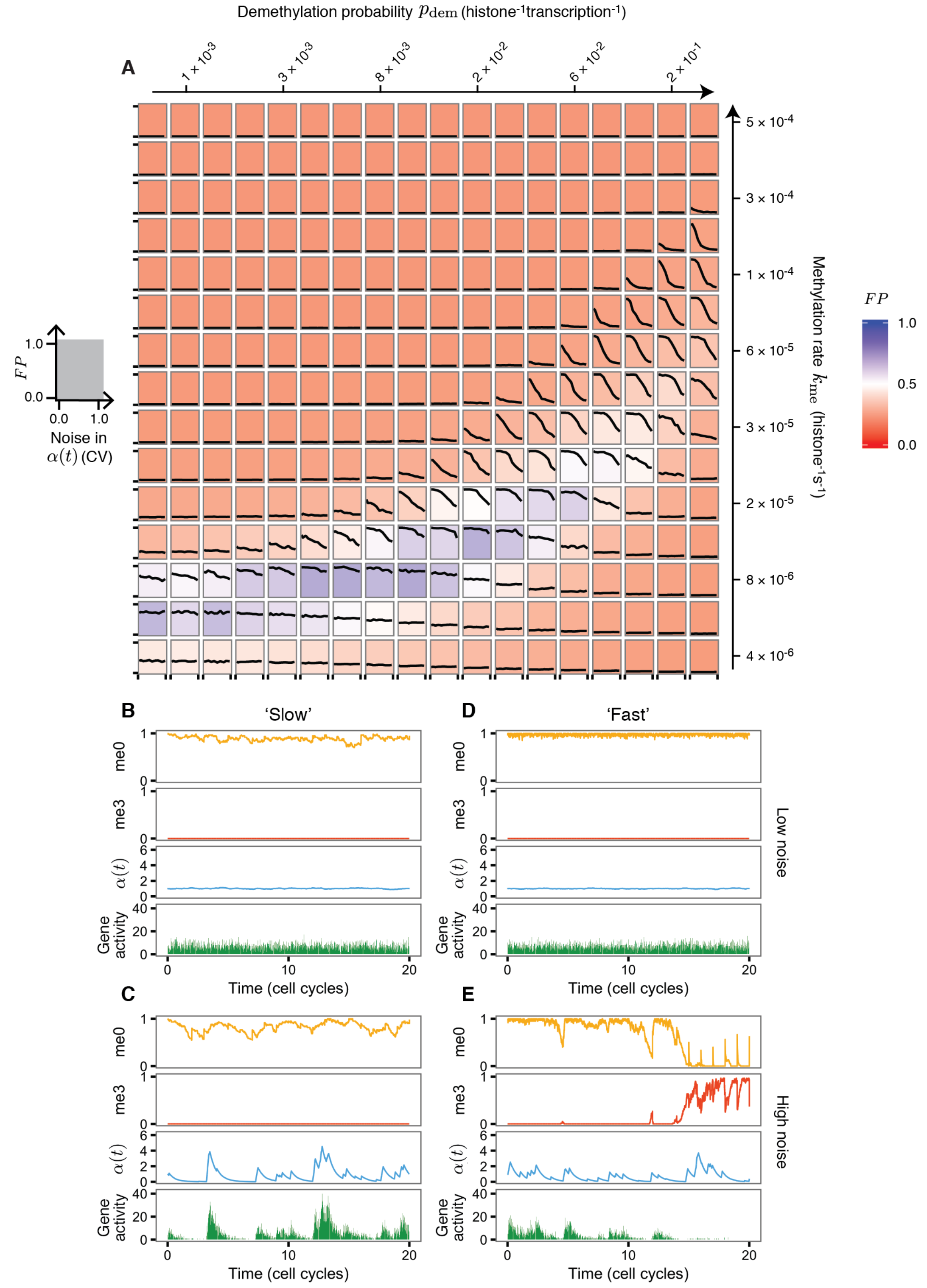

Figure S8, related to Figure 3: Effect of noisy input signal on memory-storage capability. (A) Each postage-stamp panel shows the combined first passage time measure, FP as a function of the noise in the

gene activation input signal $\alpha(t)$, plotted as a black line. Noise is measured as the coefficient of variation (CV) in input signal $\alpha(t)$. In all cases, the time-average $\langle\alpha(t)\rangle = 1$. For each parameter set, 100 simulations were initialized in each of the uniform me0 or me3 states and simulated for 25 cell cycles. The schematic postage-stamp panel shown in grey to the left indicates the values of FP and noise that are represented by the axis ticks in each panel. Each panel represents a model with different values of k_{me} and p_{dem}. k_{me} increases from bottom to top (log scale) while p_{dem} increases from left to right (log scale) – as indicated right and above, respectively. The background colour of each panel represents FP for a noisy input signal ($b = 1195$, CV ≈ 1) for the k_{me}, p_{dem} values of that panel, where b is defined in STAR methods. Blue (red) represents stable (unstable) chromatin states at high noise levels. Model and other parameters defined in Figure 1 (as modified by Equation 3) and Figures 1D and S6M ($\beta = 1$). (B-E) Example simulations with active initial state. Same as Figure 3B-E, except with active (uniform me0) initial states.

Table S1, related to Figure 1: Additional references for model formulation. Evidence is preferentially provided for mammalian Polycomb systems. Further supporting evidence from other biological systems is provided where the mammalian evidence is missing or incomplete. SUZ12, EZH2, EED, and JARID2 are core subunits of mammalian PRC2.

Model diagram with features labelled

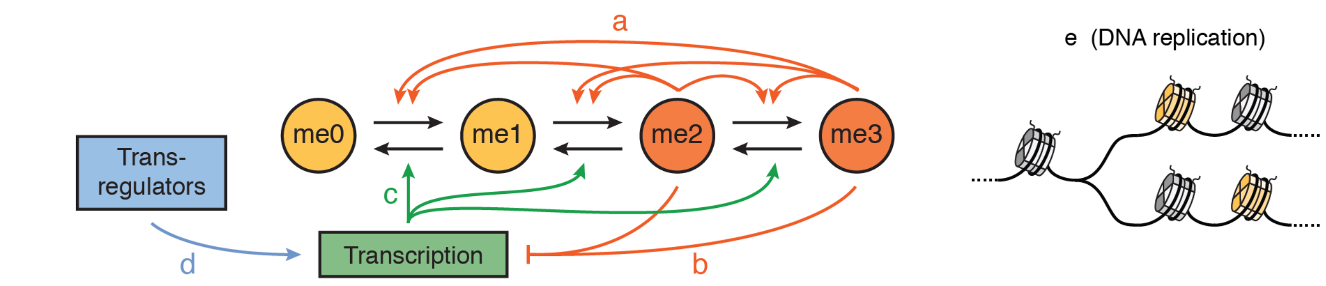

**Model feature**	**Model assumption**	**Comments**	**Biological system**	**Evidence**
a	PRC2 methylates H3K27	Firmly established	Mammalian	**Biochemical:**<br>• Purified human PRC2 methylates H3K27 *in vitro* (Cao et al., 2002; Kuzmichev et al., 2002).<br>• Catalytic efficiency of non-processive methylation of H3K27, H3K27me1, H3K27me2 substrates by human PRC2 determined *in vitro* (McCabe et al., 2012).<br>**Genetic:**<br>• PRC2 is required for all H3K27me2 and H3K27me3 *in vivo* (Ferrari et al., 2014; Jung et al., 2010; Pasini et al., 2007; Schoeftner et al., 2006), and intragenic H3K27me1 (Ferrari et al., 2014) in mouse embryonic stem (ES) cells.

				**Correlation:**<br>• SUZ12 and EED binding are correlated with H3K27me3 in human ES cells (Lee et al., 2006) and mouse embryonic fibroblasts (Boyer et al., 2006).<br>**Specific cases:**<br>• Tethering EZH2 (Hansen et al., 2008), EED (Hansen et al., 2008; van der Vlag and Otte, 1999) or JARID2 (Pasini et al., 2010a) to a reporter gene can initiate H3K27me3 accumulation and gene repression.
a	PRC2 is activated by binding H3K27me2 and H3K27me3	Well characterized *in vitro*, also with genetic evidence.	Mammalian	**Biochemical:**<br>• Binding of EED to H3K27me2 and H3K27me3 increases catalytic activity of human PRC2 *in vitro* (Margueron et al., 2009).<br>**Genetic:**<br>• Disruption of H3K27me3-binding by EED decreases H3K27me3 levels in human and mouse cells, and leads to embryonic lethality in mice (Ueda et al., 2016).
			Drosophila	**Genetic:**<br>• H3K27me2/me3 recognition by ESC (EED homologue) is required for PRC2 function *in vivo* (Margueron et al., 2009).

Table S2, related to Figure 1: Additional references for model formulation. Labeled model diagram provided in Table S1.

**Model feature**	**Model assumption**	**Comments**	**Biological system**	**Evidence**
b	H3K27me2/me3 and PRC2 repress transcription	Functionally well established yet poorly understood mechanistically.	Mammalian	**Biochemical:**<br>• Mouse PRC1 components (Grau et al., 2011) and human PRC2 (Margueron et al., 2008) can compact chromatin *in vitro*.<br>• Human PRC2 can repress transcription of chromatinized templates *in vitro* (Margueron et al., 2008).<br>**Genetic:**<br>• Mutation of SUZ12 or EED results in increased acetylation (Pasini et al., 2010b) and expression (Boyer et al., 2006; Pasini et al., 2007; Shen et al., 2008) of PRC2 target genes in mouse ES cells. However, it should also be noted that a more recent study has reported that PRC2 is dispensable for repression of PRC2 targets in certain culture conditions (but is still required during differentiation) (Riising et al., 2014).<br>• Treatment of human cells with a small molecule inhibitor of Ezh2 leads to a loss of H3K27me2/me3 and activation of PRC2 targets (Qi et al., 2012).<br>• Mutation of PRC1 or PRC2 subunits results in loss of chromatin compaction and changes in chromatin topology at Hox loci in mouse ES cells (Eskeland et al., 2010; Williamson et al., 2014).<br>**Correlation:**<br>• H3K27me3 and PRC2 occupancy are inversely correlated with markers of productive transcription, such as accumulation of mRNA (Brookes et al., 2012; Lee et al., 2006), histone acetylation (Pasini et al., 2010b), and RNA polymerase II phosphorylated on Ser-2 of the C-terminal domain (CTD) (Brookes et al., 2012) in ES cells.

				• H3K27me3 is correlated with reduced chromatin accessibility, as measured by DNAse I mapping in diverse human cells (Roadmap Epigenomics Consortium et al., 2015), or MNase accessibility (MACC) in mouse ES cells (Deaton et al., 2016).<br>**Specific case:**<br>• Tethering EZH2 (Hansen et al., 2008), EED (Hansen et al., 2008; van der Vlag and Otte, 1999) or JARID2 (Pasini et al., 2010a) to a reporter gene can initiate H3K27me3 accumulation and gene repression.
			Drosophila	**Biochemical:**<br>• PRC1 components compact chromatin (Francis et al., 2004), and inhibit chromatin remodeling *in vitro* (Francis et al., 2001).<br>• PRC1 can repress transcription *in vitro* (King et al., 2002).<br>**Genetic:**<br>• Lys-27 of H3 is required for PRC2-mediated gene repression (Pengelly et al., 2013).<br>• Mutation of ESC (EED homologue) leads to increased occupancy of RNA polymerase II and decreases in H3K27me3 at PRC2 target-gene promoters (Chopra et al., 2011).<br>**Correlation:**<br>• Polycomb silencing is associated with chromatin compaction and the formation of “long-range” intra-chromosomal contacts (Boettiger et al., 2016; Sexton et al., 2012).

Table S3, related to Figure 1: Additional references for model formulation. Labeled model diagram provided in Table S1.

**Model feature**	**Model assumption**	**Comments**	**Biological system**	**Evidence**
c	Transcription antagonizes H3K27me3 accumulation	Core assumption of our model. We cite experimental results in support of our proposal that the mechanistic basis of this antagonism is through transcription-coupled H3K27-demethylation and histone exchange.	Mammalian	**Specific cases:**<br>• Global transcriptional inhibition using small molecules results in PRC2 recruitment to new targets genome-wide in mouse ES cells (Riising et al., 2014).<br>• Transgenic reporter gene studies indicate that the transcription start site of the c-Jun locus is required for displacement of PRC2 during differentiation (Riising et al., 2014).<br>• Transcriptional induction by retinoic acid (RA) of *CYP26a1* (mouse ES cells) or *CYP26a1*, *Hoxa1*, *RARβ2* (mouse P9 embryonic carcinoma cells) results in H3K27me3 reduction (Gillespie and Gudas, 2007; Yuan et al., 2012). Conversely, H3K27me3 accumulates slowly at these genes after removal of RA.<br>• In human NIH 3T3 cells, changes in expression of PRC2 targets induced by Ras signalling precede changes in gene-body H3K27me3 levels (Hosogane et al., 2013).<br><br><u>**Histone demethylation**</u><br><br>**Biochemical:**<br>• Human JMJD3 and UTX demethylate H3K27me3 non-processively *in vitro* (Agger et al., 2007; Hong et al., 2007; Lee et al., 2007). JMJD3 associates with transcription elongation factors in human cells (Chen et al., 2012).<br><br>**Genetic:**<br>• Over-expression of human UTX reduces H3K27me2/me3 *in vivo* (Hong et al., 2007).

				**Specific cases:**<br>• UTX is bound to several *HOX* promoters (Agger et al., 2007; Lan et al., 2007) and levels increase at the *Hoxb1* locus during gene induction, resulting in H3K27me3-demethyation and loss of PRC2 (Agger et al., 2007).<br>• UTX required to maintain expression and low H3K27me2/me3 levels at *Hoxa13* and *Hoxc4* (Lee et al., 2007).<br>**<u>Histone exchange</u>**<br>**Correlations:**<br>• H3.3 histones are incorporated independently of replication in human cells and relative H3.3 levels have been regarded as a marker of histone exchange (Ray-Gallet et al., 2011; Tagami et al., 2004). H3.3 accumulation is positively correlated with transcriptional activity in human cells (Pchelintsev et al., 2013; Ray-Gallet et al., 2011).<br>• Histone exchange and H3.3 accumulation is positively correlated with transcriptional activity in mouse ES cells and neural stem cells (Deaton et al., 2016), and mouse embryonic fibroblasts (MEFs) (Kraushaar et al., 2013).<br>• Both H3.3 levels and histone exchange are negatively correlated with H3K27me3 (Kraushaar et al., 2013) in MEFs and with Polycomb complex binding in mouse ES cells (Deaton et al., 2016).
			Arabidopsis	**Specific case:**<br>• Exogenously driven transcriptional induction can remove H3K27me3 at *FLC*. Conversely, transcriptional shutdown from a high-expression state results in accumulation of H3K27me3 (Buzas et al., 2011)**.**
			S cerevisiae	**<u>Histone exchange</u>**

				**Correlation:**<br>• Histone exchange rates correlate with gene expression (Dion et al., 2007; Jamai et al., 2007).
			Drosophila	**<u>Histone demethylation</u>**<br>**Biochemical:**<br>• Drosophila UTX colocalises with elongating RNA polymerase II (Smith et al., 2008).<br>**<u>Histone exchange</u>**<br>**Correlation:**<br>• Histone exchange rates correlate with gene expression (Deal et al., 2010)

Table S4, related to Figure 1: Additional references for model formulation. Labeled model diagram provided in Table S1.

**Model feature**	**Model assumption**	**Comments**	**Biological system**	**Evidence**
d	Trans-regulators directly modulate transcription	Firmly established	Various	• In prokaryotes and eukaryotes, transcription factors can directly drive recruitment of pre-initiation complexes (reviewed in (Ptashne and Gann, 1997)) or, in eukaryotes, they can act through distal regulatory elements (reviewed in (Heintzman and Ren, 2009)).<br>• Trans-regulation can be graded in an 'analog' fashion according to dosage of a single regulator in both mammals (Giorgetti et al., 2010) and yeast (Stewart-Ornstein et al., 2013).

Table S5, related to Figure 1: Additional references for model formulation. Labeled model diagram provided in Table S1.

Model feature	Model assumption	Comments	Biological system	Evidence
e	H3/H4 tetramers are inherited at DNA replication, and are distributed with equal probability to the two daughter strands. New H3/H4 tetramers without pre-existing H3K27-methylation are inserted to fill the gaps.	Firmly established at the level of bulk chromatin. Local inheritance of H3/H4 tetramers (at individual genomic locations) is understudied.	Mammalian	• H3/H4 tetramers do not dissociate during DNA replication and segregate between DNA strands (Jackson, 1987; 1990; Jackson and Chalkley, 1985; Yamasu and Senshu, 1990) (reviewed in (Annunziato, 2005))<br>• H3K27me3 levels on parental histones are diluted by one-half immediately after DNA replication in HeLa cells, and accumulate slowly over the cell cycle (Alabert et al., 2015).<br>• H3K27-methylation is not detected before histones are incorporated into chromatin (Jasencakova et al., 2010; Loyola et al., 2006).
			C elegans	• H3K27-methylated histones are passed on and shared equally between daughter chromosomes during embryogenesis in the absence of PRC2 (Gaydos et al., 2014).
			S cerevisiae	• Parental histones are inherited relatively close to their original location (within around 400 base-pairs) (Radman-Livaja et al., 2011).

Supplemental References

Agger, K., Cloos, P.A.C., Christensen, J., Pasini, D., Rose, S., Rappsilber, J., Issaeva, I., Canaani, E., Salcini, A.E., and Helin, K. (2007). UTX and JMJD3 are histone H3K27 demethylases involved in HOX gene regulation and development. Nature *449*, 731–734.

Alabert, C., Barth, T.K., Reverón-Gómez, N., Sidoli, S., Schmidt, A., Jensen, O.N., Imhof, A., and Groth, A. (2015). Two distinct modes for propagation of histone PTMs across the cell cycle. Genes Dev. *29*, 585–590.

Annunziato, A.T. (2005). Split decision: what happens to nucleosomes during DNA replication? J. Biol. Chem. *280*, 12065–12068.

Boettiger, A.N., Bintu, B., Moffitt, J.R., Wang, S., Beliveau, B.J., Fudenberg, G., Imakaev, M., Mirny, L.A., Wu, C.-T., and Zhuang, X. (2016). Super-resolution imaging reveals distinct chromatin folding for different epigenetic states. Nature *529*, 418–422.

Boyer, L.A., Plath, K., Zeitlinger, J., Brambrink, T., Medeiros, L.A., Lee, T.I., Levine, S.S., Wernig, M., Tajonar, A., Ray, M.K., et al. (2006). Polycomb complexes repress developmental regulators in murine embryonic stem cells. Nature *441*, 349–353.

Brookes, E., de Santiago, I., Hebenstreit, D., Morris, K.J., Carroll, T., Xie, S.Q., Stock, J.K., Heidemann, M., Eick, D., Nozaki, N., et al. (2012). Polycomb associates genome-wide with a specific RNA polymerase II variant, and regulates metabolic genes in ESCs. Cell Stem Cell *10*, 157–170.

Buzas, D.M., Robertson, M., Finnegan, E.J., and Helliwell, C.A. (2011). Transcription-dependence of histone H3 lysine 27 trimethylation at the Arabidopsis polycomb target gene FLC. Plant J. *65*, 872–881.

Cao, R., Wang, L., Wang, H., Xia, L., Erdjument-Bromage, H., Tempst, P., Jones, R.S., and Zhang, Y. (2002). Role of histone H3 lysine 27 methylation in Polycomb-group silencing. Science *298*, 1039–1043.

Chen, S., Ma, J., Wu, F., Xiong, L.-J., Ma, H., Xu, W., Lv, R., Li, X., Villén, J., Gygi, S.P., et al. (2012). The histone H3 Lys 27 demethylase JMJD3 regulates gene expression by impacting transcriptional elongation. Genes Dev. *26*, 1364–1375.

Chopra, V.S., Hendrix, D.A., Core, L.J., Tsui, C., Lis, J.T., and Levine, M. (2011). The polycomb group mutant esc leads to augmented levels of paused Pol II in the Drosophila embryo. Mol. Cell *42*, 837–844.

Deal, R.B., Henikoff, J.G., and Henikoff, S. (2010). Genome-wide kinetics of nucleosome turnover determined by metabolic labeling of histones. Science *328*, 1161–1164.

Deaton, A.M., Gómez-Rodríguez, M., Mieczkowski, J., Tolstorukov, M.Y., Kundu, S., Sadreyev, R.I., Jansen, L.E., and Kingston, R.E. (2016). Enhancer regions show high histone H3.3 turnover that changes during differentiation. eLife *5*, e15316.

Dion, M.F., Kaplan, T., Kim, M., Buratowski, S., Friedman, N., and Rando, O.J. (2007). Dynamics of replication-independent histone turnover in budding yeast. Science *315*, 1405–1408.

Eskeland, R., Leeb, M., Grimes, G.R., Kress, C., Boyle, S., Sproul, D., Gilbert, N., Fan, Y., Skoultchi, A.I., Wutz, A., et al. (2010). Ring1B compacts chromatin structure and represses gene expression independent of histone ubiquitination. Mol. Cell *38*, 452–464.

Ferrari, K.J., Scelfo, A., Jammula, S., Cuomo, A., Barozzi, I., Stützer, A., Fischle, W., Bonaldi, T., and Pasini, D. (2014). Polycomb-dependent H3K27me1 and H3K27me2 regulate active transcription and

enhancer fidelity. Mol. Cell *53*, 49–62.

Francis, N.J., Saurin, A.J., Shao, Z., and Kingston, R.E. (2001). Reconstitution of a functional core polycomb repressive complex. Mol. Cell *8*, 545–556.

Francis, N.J., Kingston, R.E., and Woodcock, C.L. (2004). Chromatin compaction by a polycomb group protein complex. Science *306*, 1574–1577.

Gaydos, L.J., Wang, W., and Strome, S. (2014). H3K27me and PRC2 transmit a memory of repression across generations and during development. Science *345*, 1515–1518.

Gillespie, R.F., and Gudas, L.J. (2007). Retinoid regulated association of transcriptional co-regulators and the polycomb group protein SUZ12 with the retinoic acid response elements of Hoxa1, RARbeta(2), and Cyp26A1 in F9 embryonal carcinoma cells. J. Mol. Biol. *372*, 298–316.

Giorgetti, L., Siggers, T., Tiana, G., Caprara, G., Notarbartolo, S., Corona, T., Pasparakis, M., Milani, P., Bulyk, M.L., and Natoli, G. (2010). Noncooperative Interactions between Transcription Factors and Clustered DNA Binding Sites Enable Graded Transcriptional Responses to Environmental Inputs. Mol. Cell *37*, 418–428.

Grau, D.J., Chapman, B.A., Garlick, J.D., Borowsky, M., Francis, N.J., and Kingston, R.E. (2011). Compaction of chromatin by diverse Polycomb group proteins requires localized regions of high charge. Genes Dev. *25*, 2210–2221.

Hansen, K.H., Bracken, A.P., Pasini, D., Dietrich, N., Gehani, S.S., Monrad, A., Rappsilber, J., Lerdrup, M., and Helin, K. (2008). A model for transmission of the H3K27me3 epigenetic mark. Nat. Cell Biol. *10*, 1291–1300.

Heintzman, N.D., and Ren, B. (2009). Finding distal regulatory elements in the human genome. Curr. Opin. Genet. Dev. *19*, 541–549.

Hong, S., Cho, Y.-W., Yu, L.-R., Yu, H., Veenstra, T.D., and Ge, K. (2007). Identification of JmjC domain-containing UTX and JMJD3 as histone H3 lysine 27 demethylases. Proc. Natl. Acad. Sci. USA *104*, 18439–18444.

Hosogane, M., Funayama, R., Nishida, Y., Nagashima, T., and Nakayama, K. (2013). Ras-Induced Changes in H3K27me3 Occur after Those in Transcriptional Activity. PLoS Genet. *9*, e1003698–16.

Jackson, V. (1987). Deposition of newly synthesized histones: new histones H2A and H2B do not deposit in the same nucleosome with new histones H3 and H4. Biochemistry *26*, 2315–2325.

Jackson, V. (1990). In vivo studies on the dynamics of histone-DNA interaction: evidence for nucleosome dissolution during replication and transcription and a low level of dissolution independent of both. Biochemistry *29*, 719–731.

Jackson, V., and Chalkley, R. (1985). Histone segregation on replicating chromatin. Biochemistry *24*, 6930–6938.

Jamai, A., Imoberdorf, R.M., and Strubin, M. (2007). Continuous Histone H2B and Transcription-Dependent Histone H3 Exchange in Yeast Cells outside of Replication. Mol. Cell *25*, 345–355.

Jasencakova, Z., Scharf, A.N.D., Ask, K., Corpet, A., Imhof, A., Almouzni, G., and Groth, A. (2010). Replication stress interferes with histone recycling and predeposition marking of new histones. Mol. Cell *37*, 736–743.

Jung, H.R., Pasini, D., Helin, K., and Jensen, O.N. (2010). Quantitative mass spectrometry of histones H3.2 and H3.3 in Suz12-deficient mouse embryonic stem cells reveals distinct, dynamic post-translational modifications at Lys-27 and Lys-36. Mol. Cell. Proteomics *9*, 838–850.

King, I.F.G., Francis, N.J., and Kingston, R.E. (2002). Native and Recombinant Polycomb Group Complexes Establish a Selective Block to Template Accessibility To Repress Transcription In Vitro. Mol. Cell. Biol. *22*, 7919–7928.

Kraushaar, D.C., Jin, W., Maunakea, A., Abraham, B., Ha, M., and Zhao, K. (2013). Genome-wide incorporation dynamics reveal distinct categories of turnover for the histone variant H3.3. Genome Biol. *14*, R121.

Kuzmichev, A., Nishioka, K., Erdjument-Bromage, H., Tempst, P., and Reinberg, D. (2002). Histone methyltransferase activity associated with a human multiprotein complex containing the Enhancer of Zeste protein. Genes Dev. *16*, 2893–2905.

Lan, F., Bayliss, P.E., Rinn, J.L., Whetstine, J.R., Wang, J.K., Chen, S., Iwase, S., Alpatov, R., Issaeva, I., Canaani, E., et al. (2007). A histone H3 lysine 27 demethylase regulates animal posterior development. Nature *449*, 689–694.

Lee, M.G., Villa, R., Trojer, P., Norman, J., Yan, K.-P., Reinberg, D., Di Croce, L., and Shiekhattar, R. (2007). Demethylation of H3K27 regulates polycomb recruitment and H2A ubiquitination. Science *318*, 447–450.

Lee, T.I., Jenner, R.G., Boyer, L.A., Guenther, M.G., Levine, S.S., Kumar, R.M., Chevalier, B., Johnstone, S.E., Cole, M.F., Isono, K.-I., et al. (2006). Control of developmental regulators by Polycomb in human embryonic stem cells. Cell *125*, 301–313.

Loyola, A., Bonaldi, T., Roche, D., Imhof, A., and Almouzni, G. (2006). PTMs on H3 variants before chromatin assembly potentiate their final epigenetic state. Mol. Cell *24*, 309–316.

Margueron, R., Justin, N., Ohno, K., Sharpe, M.L., Son, J., Drury, W.J.I., Voigt, P., Martin, S.R., Taylor, W.R., De Marco, V., et al. (2009). Role of the polycomb protein EED in the propagation of repressive histone marks. Nature *461*, 762–767.

Margueron, R., Li, G., Sarma, K., Blais, A., Zavadil, J., Woodcock, C.L., Dynlacht, B.D., and Reinberg, D. (2008). Ezh1 and Ezh2 maintain repressive chromatin through different mechanisms. Mol. Cell *32*, 503–518.

McCabe, M.T., Graves, A.P., Ganji, G., Diaz, E., Halsey, W.S., Jiang, Y., Smitheman, K.N., Ott, H.M., Pappalardi, M.B., Allen, K.E., et al. (2012). Mutation of A677 in histone methyltransferase EZH2 in human B-cell lymphoma promotes hypertrimethylation of histone H3 on lysine 27 (H3K27). Proc. Natl. Acad. Sci. USA *109*, 2989–2994.

Pasini, D., Bracken, A.P., Hansen, J.B., Capillo, M., and Helin, K. (2007). The polycomb group protein Suz12 is required for embryonic stem cell differentiation. Mol. Cell. Biol. *27*, 3769–3779.

Pasini, D., Cloos, P.A.C., Walfridsson, J., Olsson, L., Bukowski, J.-P., Johansen, J.V., Bak, M., Tommerup, N., Rappsilber, J., and Helin, K. (2010a). JARID2 regulates binding of the Polycomb repressive complex 2 to target genes in ES cells. Nature *464*, 306–310.

Pasini, D., Malatesta, M., Jung, H.R., Walfridsson, J., Willer, A., Olsson, L., Skotte, J., Wutz, A., Porse, B., Jensen, O.N., et al. (2010b). Characterization of an antagonistic switch between histone H3 lysine 27 methylation and acetylation in the transcriptional regulation of Polycomb group target genes. Nucl. Acids Res. *38*, 4958–4969.

Pchelintsev, N.A., McBryan, T., Rai, T.S., van Tuyn, J., Ray-Gallet, D., Almouzni, G., and Adams, P.D. (2013). Placing the HIRA Histone Chaperone Complex in the Chromatin Landscape. Cell Rep. *3*, 1012–1019.

Pengelly, A.R., Copur, Ö., Jäckle, H., Herzig, A., and Müller, J. (2013). A histone mutant reproduces the phenotype caused by loss of histone-modifying factor Polycomb. Science *339*, 698–699.

Posakony, J.W., England, J.M., and Attardi, G. (1977). Mitochondrial growth and division during the cell cycle in HeLa cells. J. Cell Biol. *74*, 468–491.

Ptashne, M., and Gann, A. (1997). Transcriptional activation by recruitment. Nature *386*, 569–577.

Qi, W., Chan, H., Teng, L., Li, L., Chuai, S., Zhang, R., Zeng, J., Li, M., Fan, H., Lin, Y., et al. (2012). Selective inhibition of Ezh2 by a small molecule inhibitor blocks tumor cells proliferation. Proc. Natl. Acad. Sci. USA *109*, 21360–21365.

Radman-Livaja, M., Verzijlbergen, K.F., Weiner, A., van Welsem, T., Friedman, N., Rando, O.J., and van Leeuwen, F. (2011). Patterns and Mechanisms of Ancestral Histone Protein Inheritance in Budding Yeast. PLoS Biol. *9*, e1001075.

Ray-Gallet, D., Woolfe, A., Vassias, I., Pellentz, C., Lacoste, N., Puri, A., Schultz, D.C., Pchelintsev, N.A., Adams, P.D., Jansen, L.E.T., et al. (2011). Dynamics of histone H3 deposition in vivo reveal a nucleosome gap-filling mechanism for H3.3 to maintain chromatin integrity. Mol. Cell *44*, 928–941.

Riising, E.M., Comet, I., Leblanc, B., Wu, X., Johansen, J.V., and Helin, K. (2014). Gene silencing triggers polycomb repressive complex 2 recruitment to CpG islands genome wide. Mol. Cell *55*, 347–360.

Roadmap Epigenomics Consortium, Kundaje, A., Bilenky, M., Yen, A., Heravi-Moussavi, A., Zhang, Z., Wang, J., Ziller, M.J., Amin, V., Sarkar, A., et al. (2015). Integrative analysis of 111 reference human epigenomes. Nature *518*, 317–330.

Schoeftner, S., Sengupta, A.K., Kubicek, S., Mechtler, K., Spahn, L., Koseki, H., Jenuwein, T., and Wutz, A. (2006). Recruitment of PRC1 function at the initiation of X inactivation independent of PRC2 and silencing. Embo J. *25*, 3110–3122.

Sexton, T., Yaffe, E., Kenigsberg, E., Bantignies, F., Leblanc, B., Hoichman, M., Parrinello, H., Tanay, A., and Cavalli, G. (2012). Three-Dimensional Folding and Functional Organization Principles of the Drosophila Genome. Cell *148*, 458–472.

Shen, X., Liu, Y., Hsu, Y.-J., Fujiwara, Y., Kim, J., Mao, X., Yuan, G.-C., and Orkin, S.H. (2008). EZH1 mediates methylation on histone H3 lysine 27 and complements EZH2 in maintaining stem cell identity and executing pluripotency. Mol. Cell *32*, 491–502.

Smith, E.R., Lee, M.G., Winter, B., Droz, N.M., Eissenberg, J.C., Shiekhattar, R., and Shilatifard, A. (2008). Drosophila UTX is a histone H3 Lys27 demethylase that colocalizes with the elongating form of RNA polymerase II. Mol. Cell. Biol. *28*, 1041–1046.

Stewart-Ornstein, J., Nelson, C., DeRisi, J., Weissman, J.S., and El-Samad, H. (2013). Msn2 coordinates a stoichiometric gene expression program. Curr. Biol. *23*, 2336–2345.

Tagami, H., Ray-Gallet, D., Almouzni, G., and Nakatani, Y. (2004). Histone H3.1 and H3.3 complexes mediate nucleosome assembly pathways dependent or independent of DNA synthesis. Cell *116*, 51–61.

Ueda, T., Nakata, Y., Nagamachi, A., Yamasaki, N., Kanai, A., Sera, Y., Sasaki, M., Matsui, H., Honda, Z.-I., Oda, H., et al. (2016). Propagation of trimethylated H3K27 regulated by polycomb protein EED is

required for embryogenesis, hematopoietic maintenance, and tumor suppression. Proc. Natl. Acad. Sci. USA *113*, 10370–10375.

van der Vlag, J., and Otte, A.P. (1999). Transcriptional repression mediated by the human polycomb-group protein EED involves histone deacetylation. Nat. Genet. *23*, 474–478.

Williamson, I., Berlivet, S., Eskeland, R., Boyle, S., Illingworth, R.S., Paquette, D., Dostie, J., and Bickmore, W.A. (2014). Spatial genome organization: contrasting views from chromosome conformation capture and fluorescence in situ hybridization. Genes Dev. *28*, 2778–2791.

Yamasu, K., and Senshu, T. (1990). Conservative segregation of tetrameric units of H3 and H4 histones during nucleosome replication. J. Biochem. *107*, 15–20.

Yuan, W., Wu, T., Fu, H., Dai, C., Wu, H., Liu, N., Li, X., Xu, M., Zhang, Z., Niu, T., et al. (2012). Dense chromatin activates Polycomb repressive complex 2 to regulate H3 lysine 27 methylation. Science *337*, 971–975.
